# The Effect of Substitution Pattern on Binding Ability in Regioisomeric Ion Pair Receptors Based on an Aminobenzoic Platform

**DOI:** 10.3390/molecules24162990

**Published:** 2019-08-18

**Authors:** Damian Jagleniec, Krzysztof Ziach, Kajetan Dąbrowa, Jan Romański

**Affiliations:** 1 Faculty of Chemistry, University of Warsaw, Pasteura 1, 02-093 Warsaw, Poland; 2 Institute of Organic Chemistry, Polish Academy of Sciences, Kasprzaka 44/52, 01-224 Warsaw, Poland

**Keywords:** ion pair receptors, salt binding, molecular recognition, substitution effects, NMR spectroscopy, UV-Vis spectroscopy

## Abstract

A series of ditopic ion pair receptors equipped with 4-nitrophenylurea and 1-aza-18-crown-6-ether linked by ortho-(**1**), meta-(**2**), and para-(**3**) substituted benzoic acid were readily synthesized in three steps from commercially available materials. The binding properties of these regioisomeric receptors were determined using UV-vis and ^1^H NMR spectroscopy in MeCN and in the solid state by single-crystal X-ray diffraction crystallography. The solution studies revealed that, apart from carboxylates, all the anions tested formed stronger complexes in the presence of sodium cations. Receptors **2** and **3** were found to interact with ion pairs with remarkably higher affinity than *ortho*-substituted **1**. ^1^H NMR titration experiments showed that both urea NH protons interacted with anions with comparable strength in the case of receptors **2** and **3**, but only one of the NHs was effective in anion binding in the case of receptor **1**. X-ray analysis of the crystal structure of receptor **1** and **1**·NaPF_6_ complex showed that binding was hampered due to the formation of an intramolecular hydrogen bond. Analysis of the crystal structures of **2**·NaBr and **3**·NaBr complexes revealed that proper mutual orientation of binding domains was responsible for the improved binding of the sodium salts.

## 1. Introduction

The design and synthesis of heteroditopic receptors capable of simultaneously binding both counterions of an ion pair has recently emerged as an important area of supramolecular chemistry [1,2,3]. The proper embedding of distinct cation and anion recognition motifs in a single receptor entity might allow for the cooperative binding of ion pairs due to electrostatic attraction and/or allosteric effects [4,5,6,7,8,9]. In contrast, homotopic receptors possess a recognition motif exclusively for a cation or anion, hence they must compete with the counterion for binding of the corresponding ion [10]. The binding of a whole ion pair by ditopic receptor improves the salt’s lipophilicity, thus facilitating its solubilization, extraction, and membrane transport [11,12,13,14,15,16,17,18,19]. For these reasons, such systems are extremely important in the context of biology, environmental protection, and medicine [20,21,22]. Some of the most effective and selective receptors developed so far have a multi-macrocyclic (cryptand-like) structure allowing for the binding of a salt as a contact ion pair [23,24,25,26,27,28]. Application of such receptors, however, has been limited due to the complicated and usually low-yielding synthesis specific to the high dilution technique. In addition, the closed structure of these systems precludes further modification. On the other hand, the more accessible open-chain receptors can also effectively bind ion pairs, and more importantly, they are susceptible to further structural modifications. The latter feature can be used for specific purposes, such as: (i) Changing the receptor selectivity resulting from the introduction of new binding domains; (ii) recognition in an aqueous environment resulting from addition of functional groups that facilitate solubility; (iii) salt sensing achieved by introducing reporter groups to the receptor structure or immobilizing receptor molecules on the polymeric support [29,30,31,32].

Recently, we and others have reported that functionalized α–amino acids are a suitable platform for the construction of salt receptors since their framework allows for easy introduction of specific binding domains that might lead to cooperative binding of ion pairs in some cases [33,34,35,36]. Very recently, we have demonstrated that 3-aminobenzoic acid functionalized with arylthiourea and 18-aza-crown-6-ether acting as anion and cation binding units, respectively, also offer a promising platform for obtaining effective ion pair receptors [30,37]. The binding properties of the *meta* isomer were thoroughly investigated and it was found that the thiourea moiety resulted in the unfavorable deprotonation of the NH protons by even weakly basic anions.

**Figure 1:**
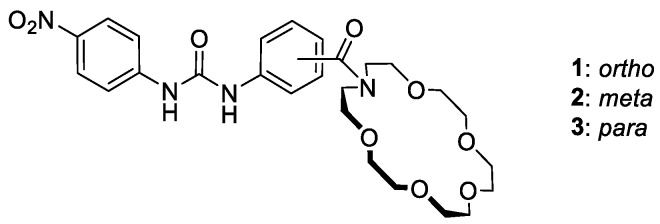
Structures of studied regioisomeric heteroditopic receptors **1**–**3**.

Our main goal in this present work was to evaluate how the spatial arrangement of ion binding domains affects anion and ion pair binding in urea-based heteroditopic receptors built on an aminobenzoic scaffold. We sought to verify if the close vicinity of binding domains located on the phenyl ring, as in the case of *ortho* regioisomer, could lead to the highest reinforcement of ion pair binding. We envisioned that in this case, formation of complexes of receptors with salts as contact ion pairs rather than separated ones should favor their binding strength. To address this question, three regioisomeric receptors consisting of a nitrophenyl urea function as an anion binding site and acylated 18-aza-crown-6-ether as a cation binding domain were investigated in depth (Figure 1).

## 2. Results and Discussion

**Scheme 1:**
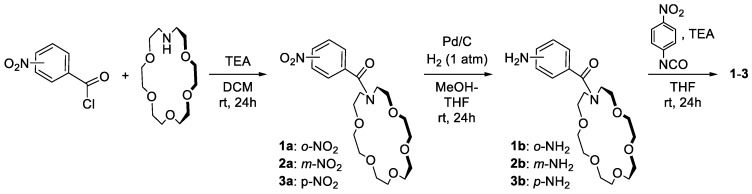
Three step synthesis of target ditopic ion pair receptors **1**–**3**.

Receptors **1**–**3** were designed so as to reinforce the anion binding by linking the crown ether unit to the receptor’s platform as an electro-withdrawing substituent. All receptors were readily prepared in three steps with good overall yields (65–68%), as outlined in Scheme 1.

Briefly, the reaction of commercially available 1-aza-18-crown ether with the *orto*-, *meta*- or *para*-nitrobenozoyl chlorides in the presence of TEA allows for the near quantitative introduction of a cation binding domain (92–97%). Subsequently, the nitro group of intermediate amides **1a**–**3a** were reduced using hydrogen gas (1 atm) and Pd/C catalyst, furnishing the corresponding amines in a near quantitative yield (95–99%). Finally, the crude amines **1b**–**3b** were reacted with 4-nitrophenylisocyanate to give receptors **1**–**3** in good yields (69–75%).

**Figure 2:**
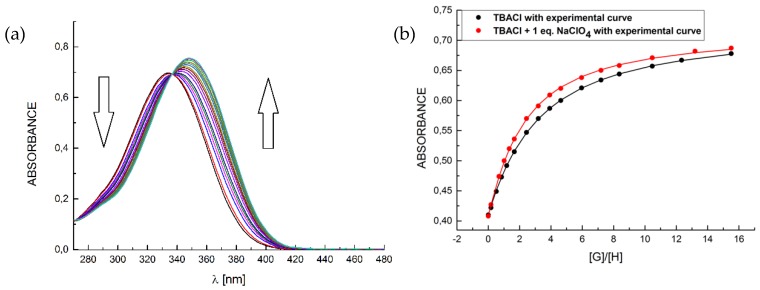
A representative binding behavior of receptor **2** upon gradual addition of TBACl: (**a**) UV-Vis titration spectra in the presence of 1 eq. of NaClO_4_ and (**b**) corresponding experimental and theoretical binding isotherms for TBACl alone (black curve) and with the presence of 1 eq. NaClO_4_ (red curve).

With receptors **1**–**3** in hand, a quantitative evaluation of their anion and salt binding abilities was carried out by means of UV-vis titrations in MeCN at 303 K. Addition of selected anions as tetrabutylammonium (TBA) salts to the receptor solutions resulted in bathochromic shifts of the absorption maxima of receptors **1**–**3** demonstrating complexes formation (Figure 2). On the other hand, test experiments with tetrabutylammonium perchlorate, consisting solely of the two common counterions for all the “active” ions in salts used in this work, ruled out the interaction of receptors with neither TBA nor ClO_4_^−^. The association constants calculated by nonlinear regression analysis of the experimental binding isotherms are listed in Table 1.

Inspection of the data in Table 1 reveals some interesting trends. Initially, we assumed that the close proximity of the cation and anion binding domains in receptor 1 should enable an electrostatic attraction between the bound ions, which should be reflected in a particularly good binding efficiency of **1** as compared with **2** and **3**. Receptor **1** was found, however, to recognize anions with stability constants at least one magnitude of order lower than for the *meta*- and *para*- substituted receptors **2** and **3**, respectively.

In addition, among the anions tested, carboxylates were bound most strongly by the receptors **1**–**3**, with a slight preference of acetate over benzoate. This is fully consistent with the known geometrical complementarity of the Y-shaped carboxylates to the urea group, as well as with the basicity of anions (MeCO_2_^−^ > PhCO_2_^−^). Then, the performance of receptors **1**–**3** towards sodium salts was examined. Taking into consideration low solubility of these salts in acetonitrile, they were prepared in situ by the prior addition of one equivalent of sodium perchlorate. In the case of receptor 1, enhancement in anion binding was evidenced for all salts tested. Despite this enhancement, however, receptor **1** was still the least powerful host for ionic guests, even in the presence of sodium cations. With the exception of carboxylates, receptors **2** and **3** were also capable of recognizing the sodium salts more strongly than the corresponding tetrabutylammonium salts. Thus we concluded that receptors **1**–**3** recognize ion pairs more effectively than anions, but in the case of receptor 1 some other interaction has a disturbing impact on the binding event.

To shed more light on the recognition behavior of receptors **1**–**3** and to explain the unexpected poor binding ability of receptor **1**, additional ^1^H NMR titration experiments were carried out in MeCN-*d*_3_ using TBABr and NaBr as representative guests. Analogously to the UV-vis experiments concerning the binding ability of receptors toward Br^−^ and NaBr, we repeated using TBABr and in situ generated sodium salt, respectively.

**Figure 3:**
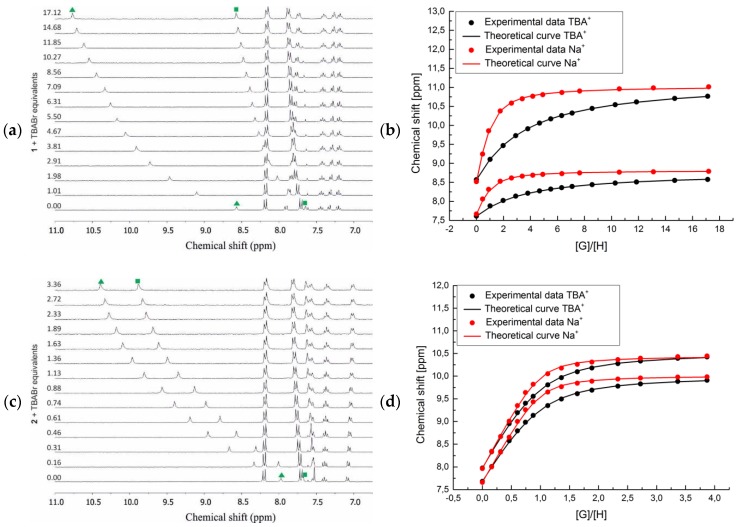

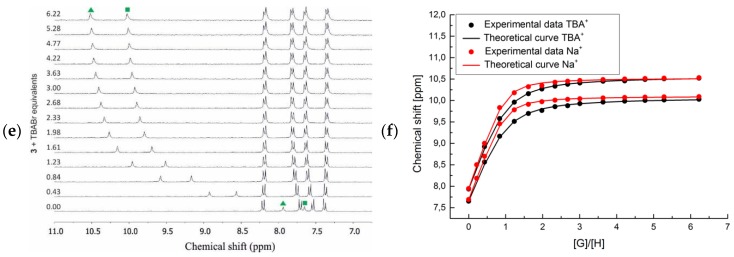
Titration of receptors **1**–**3** (from top-to-bottom) with TBABr in MeCN-*d*_3_: (**a**,**c**,**e**) Stacked ^1^H NMR spectra in the urea and aromatic C-H protons range and (**b**,**d**,**f**) corresponding binding isotherms in the absence (black curves) and presence of 1 eq. NaClO_4_ (red curves). Profiles based on the chemical shifts (δ, ppm) of the urea protons.

The first evidence for the very different behavior of receptor **1** as compared with its *meta* (**2**) and *para* (**3**) regioisomers came from the analysis of ^1^H NMR spectra without the guests added (Figure 3).

Namely, the signals corresponding to the NH urea protons in receptor **2** resonated at 7.97 and 7.68 ppm (Figure 3c, green triangle and square, respectively), and in receptor **3** at 7.94 and 7.65 ppm, respectively (Figure 3e, green triangle and square, respectively). On the other hand, one of the NH urea protons in receptor **1** was shifted downfield to 8.57 ppm, while the second one resonated at 7.65 ppm (Figure 3a, green triangle and square, respectively), i.e., comparable to the corresponding proton signals from receptors **2** and **3**. This observation suggests the occurrence of a hydrogen bonding interaction that weakens the binding affinity of **1**, since this interaction must be broken prior to binding of an anionic guest. The dilution ^1^H NMR experiments demonstrate no influence of the concentration of receptor **1** on the shifts of NH urea protons, suggesting that this interaction is intra- rather than intermolecular in nature. Furthermore, incremental addition of TBABr to the solution of receptors **2** and **3** caused a considerable downfield shift (up to 2.58 ppm) of the signals corresponding to both NH urea protons, suggesting a comparable participation of both NH’s in the anion binding event. In contrast, when receptor **1** was titrated with TBABr a very different behavior of these protons was observed (see green symbols on Figure 3a). Namely, the NH proton that initially resonated at 8.57 ppm was shifted downfield 2.28 ppm after addition of excess of bromide anions, which is comparable to the binding behavior observed for receptors **2** and **3**. Oppositely, at the same time the second NH proton of the urea group of **1**, which initially resonated at 7.65 ppm, was shifted only to 8.60 ppm.

This supports the hypothesis that one of the protons is engaged in the intramolecular hydrogen bond, presumably with the carbonyl group linking the crown ether unit with the receptor scaffold. However, there is ambiguity in assigning which proton is directly involved in this interaction. Initially, we assumed that this N*_out_*-H proton, which resonated at 8.67 ppm, is neighboring the phenyl ring possessing crown ether unit and the downfield shift is a symptom of the formation of hydrogen bonding. However, the second proton of the urea group (N*_in_*-H) shifted much less upon addition of bromide anions, which suggests its participation in an interaction other than anion binding. To address this question, 2D NMR spectra were carried out, confirming the latter case. In particular, in HMBC spectrum in MeCN-*d*_3_ (see Figures S27 and S28 in ESI for details), the N*_in_*-H urea proton (*δ* = 7.68 ppm) correlated uniquely with the carbons of the adjacent aromatic ring of aminobenzoic acid (*δ* = 124.6, 130.0 ppm), while N*_out_*-H (*δ* = 8.67 ppm) showed strong correlations with carbons of the nitrophenyl ring (*δ* = 118.4 ppm).

Interestingly, in all cases, perturbation in the position of aromatic protons was observed upon titrations but the changes were less distinct. This is likely due to the internal C−H···O hydrogen bonding between aromatic protons and carbonyl oxygens of the urea groups. Based on the analysis of proton shifts induced upon addition of TBABr, the corresponding stability constants were determined and their values were found to be consistent with those obtained by UV-vis measurements (Table 2). 

Interestingly, we found that for all receptors **1**–**3** the analogous titrations with bromide in the presence of one equivalent of sodium cation caused a higher enhancement in bromide binding as compared with experiments carried out under UV-vis control on more diluted samples. This demonstrates that proper design of ion pair receptors can provide enhancement in anion binding in the presence of sodium cation, which is especially pronounced at higher concentrations (^1^H NMR titrations) where the fraction of receptors occupied by cation is much higher (Table 3). 

**Figure 4:**
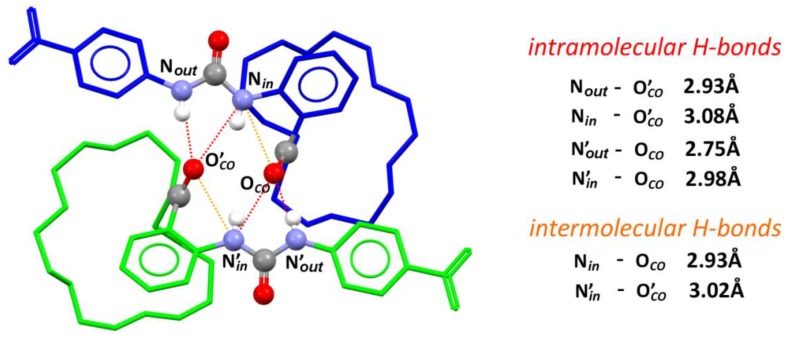
The crystal structure of dimer formed by receptors **1** in the solid state: Conformers A and B are colored in green and blue, respectively; urea and carbonyl groups are presented as balls in common colors; solvent molecules, most of hydrogen atoms, and disorder are omitted for clarity; selected hydrogen bonds are presented as dashed lines connecting corresponding donor and acceptor groups.

**Figure 5:**
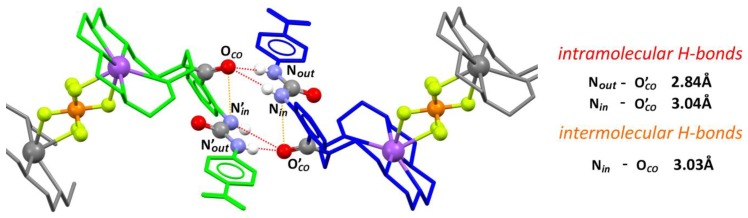
The crystal structure of dimer formed by complexes of **1**⋅NaPF_6_ in the solid state: Green, blue, and gray color are used to distinguish between identical receptor molecules involved in the urea-carbonyl dimer formation and, further, a supramolecular polymer; urea and carbonyl groups are presented as balls in common colors; solvent molecules, most of hydrogen atoms, and disorder were omitted for clarity; selected hydrogen bonds are presented as red lines connecting corresponding donor and acceptor groups.

**Figure 6:**
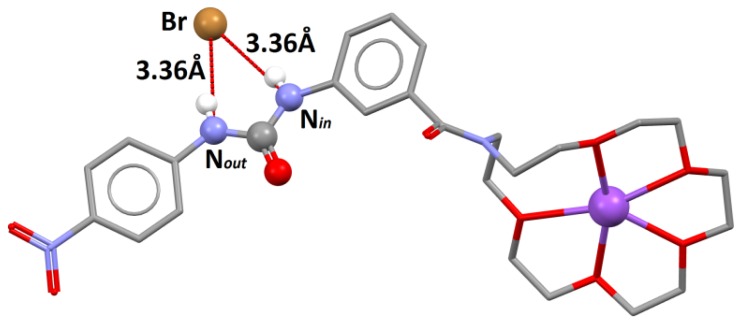
The crystal structure of **2**⋅NaBr: Urea and carbonyl groups are presented as balls; solvent molecules, most of the hydrogen atoms, and disorder were omitted for clarity; selected hydrogen bonds are presented as red lines connecting corresponding donor and acceptor groups.

**Figure 7:**
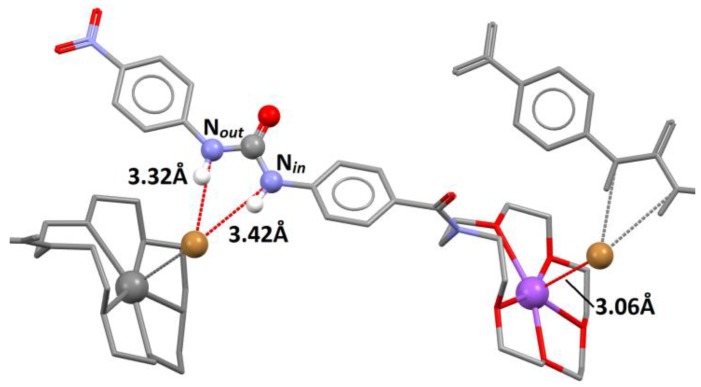
The crystal structure of **3**⋅NaBr: Urea and carbonyl groups are presented as balls; solvent molecules, most of the hydrogen atoms, and disorder were omitted for clarity; selected hydrogen bonds are presented as red lines connecting corresponding donor and acceptor groups.

Further rationalization for the influence of the substitution pattern on the complexation ability of aminobenzoic acid-derived receptors comes from the analysis of the X-ray crystal structures of free ligand **1** (Figure 4), its NaPF_6_ complex (Figure 5), and NaBr complexes with receptors **2** (Figure 6) and **3** (Figure 7). 

In the crystal lattice, receptor **1** exists in two similar, yet not identical, conformations, A and B (see ESI for details). Conformers A and B together form a dimer through the strong NH_urea_ – CO_amide_ hydrogen bonds, and the corresponding inter atomic distances are: N*_out_*A-O*_CO_*B = 2.75 Å, N*_in_*A-O*_CO_*B = 2.98 Å, N*_out_*B-O*_CO_*A = 2.93 Å, and N*_in_*B-O*_CO_*A = 3.08 Å (Figure 4). More pronouncedly, one of the urea NH protons, named N*_in_*, is intramolecularly hydrogen-bonded to the amide oxygen atom and the corresponding inter atomic distances are N*_in_*A-O*_CO_*A = 3.02 Å and N*_in_*B-O*_CO_*B = 2.93 Å. As a consequence, the bonding propensity of the N*_in_* urea proton is presumably weakened, which is reflected by the substantial lengthening of the intermolecular hydrogen bonds, as compared to the N*_out_* urea proton, by 0.23 Å and 0.15 Å for conformers A and B, respectively. These solid state data fully correspond to the significantly lower anion binding affinity of *ortho*-substituted receptor **1** as compared with its *meta* (**2**) and *para* (**3**) counterparts, in which such an intramolecular hydrogen bond cannot be formed. The crown ether moiety of receptor **1**, in the absence of cationic guest, is empty and folded, and interacts with the central ring of aminobenzoic acid residue through ring CH-O*_crown_* interactions (corresponding C*_2Arom_*-O*_crown_* distances are 3.55 Å and 3.35 Å for conformers A and B, respectively; see: Figure S22 in ESI). There are also CH-Π interactions formed by the crown ether C-H and aromatic ring, which correspond to interatomic distances: C*_crown_*-Π*_arom_* = 3.70 Å for conformer A and 3.76 Å and 3.92 Å for conformer B.

Despite substantial efforts, we were not able to obtain monocrystals of complexes of **1** with sodium salts of the anions in question. However, some further information regarding the complexation properties of **1** come from X-ray crystal structure analysis of **1**·NaPF_6_ complex (Figure 5). 

Most profoundly, in this complex the intramolecular hydrogen bond between the urea N*_in_*H and carbonyl oxygen group was still present (N*_in_*-O*_CO_* = 3.03 Å). Similarly to the crystal structure of free receptor **1** (Figure 4), the organic ligand also formed a dimer in which the hydrogen bonds occur between the urea NH protons and an amide oxygen of the second molecule, and bonding involving the N*_out_* was substantially stronger than for N*_in_* (2.84 Å vs. 3.04 Å, respectively). Sodium cation occupied the cavity of the crown ether and the geometry and symmetry of the macrocyclic cavity resembled the 15-crown-5 ether more than the 18-crown-6 ether, which has been already observed in similar receptor systems [30]. The presence of cation in the crown cavity strongly disrupted the C-H based interaction between the macrocycle and aminobenzoic acid scaffold, which was observed for an unoccupied receptor **1**. In a crystal lattice, a supramolecular polymer of “head-to-head” and “tail-to-tail” type is formed based on the above-described urea-amide interactions and bridging of crown ether sodium complexes of two ligands by one of the PF_6_^−^ anions. 

Analysis of the crystal structures of **2**·NaBr (Figure 6) and 3·NaBr (Figure 7) complexes fully reflects the strong affinity of these regioisomeric receptors to NaBr observed in the solution. 

Namely, in the former crystal structure the bromide anion was strongly bonded by both urea NH protons with comparable strength, as reflected by virtually the same N-Br distances (3.36 Å). As expected, sodium cation occupied the cavity of the crown ether, which again adopted a 15-crown-5-like geometry. 

In the unit cell of **3**·NaBr there are four conformers of receptor **3**, distinguished as A, B, C, and D (Figure 7).

Conformer A was arbitrarily chosen for further analysis, but given the very high level of resemblance among them (see ESI for details) all conclusions are valid for each of the conformers. Similarly as in the case of **2**·NaBr, the cation and anion guests occupied the designed binding domains. Bromide anion was hydrogen-bonded by urea protons, but in this case the interatomic distances differed for N*_in_*H and N*_out_*H by 0.1 Å in favor of the latter (N*_in_*-Br = 3.32 Å and N*_out_*Br = 3.42 Å). Bromide was also bonded to sodium cations residing in another receptor molecule. The NaBr distance equaled 3.06 Å, which is substantially longer than in the previously reported NaBr complexes of furan derived macrocycles (*d*_NaBr_ = 2.75 Å and 2.78 Å), which had no anion domain [38]. Bridging between sodium and bromide ions from the neighboring receptor molecules formed a supramolecular polymer of the “head-to-tail” type in the solid state.

## 3. Materials and Methods

### 3.1. General Methods

All reagents and chemicals were of reagent-grade quality and purchased commercially. Solvents were dried using common laboratory techniques. ^1^H and ^13^C NMR spectra were recorded on a Bruker 300 MHz spectrometer (Bruker Corporation, Billerica, MA, USA). Reaction monitoring and initial substrates purity assessment was done using Magritek SpinSolve 43 MHz NMR system (Magritek, Aachen, Germany). ^1^H NMR chemical shifts δ are reported in parts per million, referenced to residual solvent signal (deuterated dimethyl sulfoxide (DMSO-*d*_6_), MeCN-*d*_3_, or CDCl_3_). High-resolution mass spectra (HRMS) were measured on a LCT (TOF) Micromass unit (Waters Corporation, Milford, CT, USA) using electrospray ionization (ESI) technique. Compounds **2a**–**3a** and **2b**–**3b** were prepared according to procedures reported previously [39].

### 3.2. General Procedure A—For Preparation of Compounds ***1a**–**3a***

Firstly, 1-aza-18-crown-6 (0.72 g, 2.69 mmol) at 0 °C (ice bath) was added to a solution of corresponding nitrobenzoyl chloride (0.36 mL, 2.72 mmol) and TEA (0.38 mL, 2.72 mmol) in DCM (20 mL). The reaction mixture was stirred for 30 min and then left at room temperature (r.t.) overnight. The organic phase was then washed with distilled water (2 × 30 mL), 1 M HCl (30 mL), saturated aqueous solution of NaHCO_3_ (30 mL), and dried over MgSO_4_. After evaporation of DCM, the residue was purified by silica gel column chromatography using a mixture of MeOH in CHCl_3_ (5:95 *v*/*v*) to give the title product as a colorless oil.

**Compound 1a**. According to general procedure A and using 2-nitrobenzoylchloride (0.36 g, 2.72 mmol), TEA (0.38 mL, 2.72 mmol), and 1-aza-18-crown-6 (0.72 g, 2.69 mmol) in DCM (20 mL), the title compound was prepared as a colorless oil (1.02 g, 92%). ^1^H NMR (300 MHz, CDCl_3_) *δ* 8.13–8.22 (m, 1H), 7.63–7.72 (m, 1H), 7.48–7.58 (m, 1H), 7.38–7.46 (m, 1H), 3.83 (s, 4H), 3.38–3.72 (m, 20H).^13^C NMR (75 MHz, CDCl_3_) *δ* 168.2, 145.1, 134.3, 133.4, 129.6, 128.6, 124.7, 77.5, 77.1, 76.6, 70.9, 70.7 70.6, 70.5, 70.4, 70.3, 69.3, 68.8, 49.6, 45.6.HRMS (ESI): Calculated for C_19_H_28_N_2_O_8_Na [M + Na]^+^: 435.1743, found: 435.1751.
**Compound 2a**. According to general procedure A and using 3-nitrobenzoylchloride (1.00 g, 5.39 mmol), TEA (0.90 mL, 6.45 mmol), and 1-aza-18-crown-6 (1.42 g, 5.40 mmol) in DCM (50 mL), the title compound was prepared as a colorless oil (2.15 g, 97%). ^1^H NMR (300 MHz, DMSO–*d_6_*) *δ* 8.20–8.35 (m, 2H), 7.80–7.89 (m, 1H), 7.71–7.77 (m, 1H), 3.40–3.80 (m, 24H). ^13^C NMR (75 MHz, DMSO–*d_6_*) *δ*169.2, 148.0, 138.9, 133.8, 130.6, 124.3, 122.2, 70.6, 70.4, 70.2, 68.7, 50.1, 45.7. HRMS (ESI): Calculated for C_19_H_28_N_2_O_8_Na [M + Na]^+^: 435.1743, found: 435.1747.
**Compound 3a.** According to general procedure A and using 4-nitrobenzoylchloride (1.00 g, 5.40 mmol), TEA (0.90 mL, 6.40 mmol), and 1-aza-18-crown-6 (1.42 g, 5.40 mmol) in DCM (50 mL), the title compound was prepared as a colorless oil (2.10 g, 95%). ^1^H NMR (300 MHz, CDCl_3_) *δ* 8.22–8.35 (m, 2H),7.29–7.37(m, 2H),3.66–3.81 (m, 24H). ^13^C NMR (75 MHz, CDCl_3_) *δ* 156.3, 155.3, 144.7, 125.0, 122.3, 70.8, 70.7, 70.6,70.5, 70.4, 70.2, 69.5, 69.4 48.8, 48.5.HRMS (ESI): Calculated for C_19_H_28_N_2_O_8_Na [M + Na]^+^: 435.1743, found: 435.1750.

### 3.3. General Procedure B—For Preparation of Compounds ***1b**–**3b***

A 10% palladium on carbon catalyst (50 mg, 5% wt) was added to a degassed solution of corresponding nitro compound **1a**–**3a** (1.00 g, 2.42 mmol) in a THF/MeOH mixture (150 mL, 1:4 *v*/*v*). The reaction mixture was stirred under H_2_ atmosphere (1 atm.) at r.t. overnight. The catalyst was removed by filtration through a pad of Celite^®^ and washed with MeOH (50 mL). The filtrate was concentrated under reduced pressure to give the crude product in a quantitative yield (0.92 g). The corresponding amine was used in next step without further purification.

**Compound 1b**: HRMS (ESI): Calculated for C_19_H_30_N_2_O_6_Na [M + Na]^+^: 405.2002, found: 405.2011.
**Compound 2b**: HRMS (ESI): Calculated for C_19_H_30_N_2_O_6_Na [M + Na]^+^: 405.2002, found: 405.2008.
**Compound 3b**: HRMS (ESI): Calculated for C_19_H_30_N_2_O_6_Na [M + Na]^+^: 405.2002, found: 405.2006.

### 3.4. General Procedure C—For Preparation of Receptors ***1**–**3***

TEA (0.12 mL, 1.10 mmol) was added to the solution of corresponding amine (0.34 g, 0.89 mmol) and nitrophenylisocyanate (0.15 g, 0.89 mmol) in THF (20 mL). The reaction was stirred overnight at r.t. and the reaction mixture was concentrated under reduced pressure giving a solid residue, which was purified by silica gel column chromatography using a mixture of MeOH in CHCl_3_ (2:98 *v*/*v*) to give the target receptor as a yellow oil.

**Receptor 1.** According to general procedure C and using amine **1b** (0.34 g, 0.89 mmol), 2-nitrophenylisocyanate (0.15 g, 0.89 mmol), and TEA (0.12 mL, 1.10 mmol) in THF (20 mL), the title compound was prepared as a yellow oil (0.37 g, 75%). ^1^H NMR (300 MHz, DMSO–*d*_6_) *δ* 10.06 (s, 1H), 8.25–8.17 (m, 3H), 7.86–7.80 (m, 1H), 7.71–7.64 (m, 2H), 7.45–7.33 (m, 1H), 7.32–7.25 (m,1H), 7.20–7.10 (m, 1H), 3.70 (s, 4H), 3.32–3.59 (m, 20H). ^13^C NMR (75 MHz, CDCl_3_) *δ* 171.4, 152.4, 145.8, 142.0, 134.9, 130.2, 128.1, 126.6, 125.0, 123.9, 123.6, 117.6, 70.5, 70.0, 69.0, 68.5, 50.2, 45.4. HRMS (ESI): Calculated for C_26_H_34_N_4_O_9_Na [M + Na]^+^: 569.2224, found: 569.2229.
**Receptor 2.** The compound was prepared using modified procedure described previously **[18]**. According to general procedure C and using amine **2b** (0.40 g, 1.05 mmol), 3-nitrophenylisocyanate (0.18 g, 1.10 mmol), and TEA (0.15 mL, 1.10 mmol) in THF (30 mL), the title compound was prepared as a yellow oil (0.40 g, 70%). ^1^H NMR (300 MHz, CDCl_3_) *δ* 8.83 (s, 1H), 8.21 (s, 1H), 8.15–8.08 (m, 2H), 7.58–7.50 (m, 2H), 7.40–7.29 (m, 2H), 7.26–7.18 (m, 1H), 7.03–6.96 (m, 1H), 3.70–3.60 (m, 24H). ^13^C NMR (75 MHz, CDCl_3_) *δ* 173.2, 152.1, 145.8, 142.0, 139.2, 136.5, 129.4, 125.1, 120.6, 120.5 117.6, 117.1, 70.7, 70.6, 70.5, 70.3. 69.2 HRMS (ESI): calcd for C_26_H_34_N_4_O_9_Na [M + Na]^+^: 569.2224, found: 569.2229. HRMS (ESI): Calculated for C_26_H_34_N_4_O_9_Na [M + Na]^+^: 569.2224, found: 569.2202.
**Receptor 3.** According to general procedure C and using amine **3b** (0.56 g, 1.45 mmol), 4-nitrophenylisocyanate (0.24 g, 1.40 mmol), and TEA (0.15 mL, 1.10 mmol) in THF (30 mL), the title compound was prepared as a yellow oil (0.55 g, 69%). ^1^H NMR (300 MHz, CDCl_3_) *δ* 8.98 (s, 1H), 8.37 (s, 1H), 8.25–8.15 (m, 2H), 7.70–7.61 (m, 2H), 7.26–7.20 (m, 2H), 7.14–7.07 (m, 2H), 3.86–3.62 (m, 24H). ^13^CNMR (75 MHz,CDCl_3_) *δ* 173.5, 152.2, 145.7, 142.1, 140.4, 127.5, 125.1, 119.4, 117.7, 70.9, 70.7, 70.6, 70.4, 69.1.HRMS (ESI): Calculated for C_26_H_34_N_4_O_9_Na [M + Na]^+^: 569.2224, found: 569.2210.

### 3.5. UV-Vis Titration Procedure

UV–vis titrations were performed in acetonitrile using a Thermo Spectronic Unicam UV500 Spectrophotometer (Thermo Fisher Scientific, Waltham, USA). In a typical experiment, 2.5 mL of a freshly prepared 3.52 × 10^−5^ M solution of corresponding receptor **1**–**3** was added to a cuvette. Then, small aliquots of tetrabutylammonium (TBA) salts of corresponding anions (Cl^−^, Br^−^, NO_2_^−^, PhCOO^−^, MeCOO^−^), containing a constant concentration of the receptor, were added, and a spectrum was acquired after each addition. Where applicable, the solution also contained 1 molar equivalent of NaClO_4_. The resulting titration data were analyzed using the BindFit (version 0.5) package, available online [40].

### 3.6. ^1^H NMR Titration Procedure

NMR titration experiments were conducted in MeCN-*d*_3_. In a typical experiment, 0.5 mL of a freshly prepared 2.92 mM solution of corresponding receptor **1**–**3** was added to a 5 mm NMR tube. In the case of ion pair titration, the solution also contained 1 molar equivalent of NaClO_4_. Then, small aliquots of a solution of TBABr, containing corresponding receptor **1**–**3** at a constant concentration, were added, and a spectrum was acquired after each addition. The resulting titration data were analyzed using the BindFit (version 0.5) package, available online [40].

### 3.7. Crystallographic Measurements

The X-ray measurements of **1**, **1**·NaPF_6_, **2**·NaBr, and **3**·NaBr crystals were performed on a Bruker D8 Venture Photon100 diffractometer (Bruker Corporation, Billerica, USA) equipped with a TRIUMPH monochromator and a MoKα fine focus sealed tube (λ = 0.71073 Å). Data collection, reduction, and analysis were carried out with Bruker programs [41,42]. Data were corrected for absorption effects using the multiscan method (SADABS) [43]. The structures were solved and refined using SHELXTL Software Package (Bruker Corporation, Billerica, USA) [44,45]. Unless stated otherwise, the non-hydrogen atoms were refined anisotropically and hydrogen atoms were placed in calculated positions and refined within the riding model. The temperature factors of these hydrogen atoms were not refined and were set to be equal to either 1.2 or 1.5 times larger than Ueq of the corresponding heavy atom. The atomic scattering factors were taken from the International Tables [46]. Selected crystal data and structure refinement parameters for obtained crystals are summarized below. More details regarding data collection, structure refinement, and final crystal data can be found in Supplementary Information.

X-ray **1**. Monocrystal was obtained by a slow vapour–vapour diffusion of *n*-pentane into a solution of **1** (5.5 mg, 10 µmol) in CHCl_3_ (0.5 mL) at 4 °C. C_53.83_H_69.83_Cl_5.49_N_8_O_18_, M = 1311.58, triclinic, P-1, a = 13.9190(9) Å, b = 15.5110(10) Å, c = 16.3591(10) Å, α = 71.369(2)°, β = 73.035(2)°, γ = 67.080(2)°, V = 3025.4(3) Å^3^, T = 100(2) K, Z = 2, μ(Mo-Kα) = 0.339 mm^−1^, 48072 collected reflexes, 10732 unique (R_int_ = 0.0223). Final R indices [I > 2σ(I)]: R1(F^2^) = 0.0529 and wR2(F^2^) = 0.1236, R indices (all data) R1(F^2^) = 0.0662 and wR2(F^2^) = 0.1349. CCDC deposition number: 1923901.

X-ray **1**·NaPF_6_. Monocrystal was obtained by a slow vapour–vapour diffusion of Et_2_O into a solution of **1** (4.1 mg, 7.5 µmol) and NaPF_6_ (1 equiv., 1.3 mg, 7.5 µmol) in MeCN (0.4 mL) at r.t. C_59_H_82.50_F_12_N_9.50_Na_2_O_19_P_2_, M = 1564.76, triclinic, P-1, a = 8.4135(4) Å, b = 13.0050(6) Å, c = 16.7431(8) Å, α = 104.0055(12)°, β = 92.4228(13)°, γ = 92.5966(13)°, V = 1773.02(15) Å^3^, T = 100(2) K, Z = 1, μ(Mo-Kα) = 0.181 mm^−1^, 39203 collected reflexes, 6246 unique (R_int_ = 0.0298). Final R indices [I > 2σ(I)]: R1(F^2^) = 0.0509 and wR2(F^2^) = 0.1227, R indices (all data) R1(F^2^) = 0.0647 and wR2(F^2^) = 0.1328. CCDC deposition number: 1923898.

X-ray **2**·NaBr. Monocrystal was obtained by a slow vapour–vapour diffusion of Et_2_O into a solution of **2** (5.5 mg, 10 µmmol) and NaBr (1 equiv., 1.0 mg, 10 µmol) in CHCl_3_ (1.0 mL) and DMSO (0.3 mL) mixture at r.t. C_33.36_H_54.73_BrN_4_NaO_12_S_2.32_, M = 881.04, triclinic, P-1, a = 12.7282(10) Å, b = 13.6326(11) Å, c = 14.5482(12) Å, α = 106.806(2)°, β = 113.890(2)°, γ = 102.082(2)°, V = 2050.1(3) Å^3^, T = 100(2) K, Z = 2, μ(Mo-Kα) = 1.193 mm^−1^, 47583 collected reflexes, 7246 unique (R_int_ = 0.0249). Final R indices [I > 2σ(I)]: R1(F^2^) = 0.0286 and wR2(F^2^) = 0.0654, R indices (all data) R1(F^2^) = 0.0343 and wR2(F^2^) = 0.0688. CCDC deposition number: 1923900.

X-ray **3**·NaBr. Monocrystal was obtained by a slow vapour–vapour diffusion of diethyl ether into a solution of **3** (4.1 mg, 7.5 µmol) and NaBr (1 equiv., 0.8 mg, 7.5 µmol) in MeOH (0.4 mL) at r.t. C_27_H_38_BrN_4_NaO_10_, M = 681.51, monoclinic, P21/n, a = 15.488(2) Å, b = 14.836(2) Å, c = 55.956(9) Å, α = 90°, β = 96.5751(19)°, γ = 90°, V = 12773.0(3) Å^3^, T = 100(2) K, Z = 16, μ(Mo-Kα) = 1.360 mm^−1^, 212011 collected reflexes, 22597 unique (R_int_ = 0.0787). Final R indices [I > 2σ(I)]: R1(F^2^) = 0.0680 and wR2(F^2^) = 0.1343, R indices (all data) R1(F^2^) = 0.0886 and wR2(F^2^) = 0.1408. CCDC deposition number: 1923899.

## 4. Conclusions

In conclusion, we have presented the synthesis of three regioisomeric urea-based heteroditopic receptors **1**–**3** possessing a crown ether unit and studied their anion and ion pair recognition characteristics in solution and in the solid state. Detailed solution studies with anions and ion pairs showed enhancement in anion binding by receptors **1**–**3** upon simultaneous complexation of sodium cation. Receptors **2** and **3**, possessing binding domains in *meta* and *para* position, were found to interact with anions and ion pairs with a remarkably higher affinity than the *ortho*-substituted receptor **1**. The weakness in anion and ion pair recognition by receptor **1** was attributed to the formation of an intramolecular hydrogen bond between one of the urea protons and the carbonyl group of amide function. This was evidenced by ^1^H NMR measurements, which showed that upon titration of receptor **1** with anions and ion pairs the ability of one of the NHs to interact with anions was hampered. Solid state analyses supported this finding and showed presence of this intramolecular interaction in the crystal structures of both free receptor **1** and its NaPF_6_ complex. On the other hand, the orientation of the binding domains, as in the *meta* or *para* receptors **2** and **3**, resulted in effective interaction of ion pairs with binding domains, as it was evidenced by X-ray analyses of **2·**NaBr and **3**·NaBr complexes.

## Figures and Tables

**Table 1:** Association constants (log*K*_a_) for the interactions between receptors **1**–**3** and selected anions in the absence or in the presence of one equivalent of NaClO_4_^[a]^.

Anion	**1**	1 + Na^+^	*K*_Na_/*K*_TBA_
Cl^−^	2.18	2.62	2.74
Br^−^	2.27	2.60	2.16
NO_2_^−^	2.37	2.52	1.44
PhCO_2_^−^	4.38	4.44	1.14
MeCO_2_^−^	4.39	4.52	1.34
	**2**	**2** + Na^+^	*K*_Na_/*K*_TBA_
Cl^−^	4.05	4.21	1.45
Br^−^	3.21	3.38	1.49
NO_2_^−^	3.92	4.08	1.44
PhCO_2_^−^	4.82	4.76	0.87
MeCO_2_^−^	5.77	5.74	0.92
	**3**	**3** + Na^+^	*K*_Na_/*K*_TBA_
Cl^−^	4.07	4.11	1.10
Br^−^	3.29	3.38	1.25
NO_2_^−^	3.92	3.98	1.15
PhCO_2_^−^	5.26	5.20	0.88
MeCO_2_^−^	5.89	5.79	0.79

^[a]^ Determined using UV-Vis titration experiments in MeCN at 293 K; [1] = 3.52 × 10^−5^ M; [2] = 2.98 × 10^−5^ M; [3] = 3.45 × 10^−5^ M; anions used as TBA salts; [TBAX] ~ 10^−3^ M; 1:1 binding model was used for nonlinear regression fitting of the experimental data; estimated errors <10%.

**Table 2:** Association constants (log*K_a_*) for the interaction between receptors **1**–**3** with TBABr in the absence or presence of one equivalent of NaClO_4_^[a]^.

Guest	1	2	3
TBABr	2.04	3.36	3.41
NaBr ^[b]^	3.02	3.88	3.84
*K*_Na_/*K*_TBA_	9.54	3.26	2.67

^[a]^ Determined using ^1^H NMR titration experiments in MeCN-*d*_3_ at 293 K; [1] = 2.92 mM; [2] = 2.73 mM; [3] = 2.66 mM; salts; [TBAX] <20 mM; 1:1 binding model was used for nonlinear regression fitting of the experimental data; estimated errors <10%. ^[b]^ One equivalent of NaClO_4_ was added prior to titration with TBABr.

**Table 3:** Association constants (log*K_a_*) for the interaction between receptors **1**–**3** and NaClO_4_ and calculated % fraction of receptor occupied by the sodium cation under UV-vis and ^1^H NMR titration conditions.

	1	2	3
logK_Na+_	3.85	4.08	4.06
C_UV-Vis_ [mM]	0.035	0.029	0.035
X_UV-Vis_^[a]^ [%]	17	21.9	23.3
C_NMR_ [mM]	2.92	2.73	2.66
X_NMR_^[a]^ [%]	80.2	84	83.5

^[a]^ The coefficient of the receptor occupation by Na^+^ was determined by equation: X = 100% [Receptor-Na^+^] [Receptor]^−1^.

## Supplementary Materials

The following are available online: ^1^H and ^13^C NMR spectra, UV–vis and ^1^H NMR titration spectra, UV–vis and NMR binding isotherms, crystal data of [1], [1·NaPF_6_], [2·NaBr], and [3·NaBr] complexes.
