# Supplementary material for: The Effect of Substitution Pattern on Binding Ability in Regioisomeric Ion Pair Receptors Based on an Aminobenzoic Platform
Source: Molecules. 2019 Aug 18;24(16):2990. doi: 10.3390/molecules24162990 (PMC6720599; doi:10.3390/molecules24162990)

checkCIF/PLATON report

You have not supplied any structure factors. As a result the full set of tests cannot be run.

THIS REPORT IS FOR GUIDANCE ONLY. IF USED AS PART OF A REVIEW PROCEDURE FOR PUBLICATION, IT SHOULD NOT REPLACE THE EXPERTISE OF AN EXPERIENCED CRYSTALLOGRAPHIC REFEREE.

No syntax errors found. CIF dictionary Interpreting this report

Datablock: complex_1_NaPF6

```
Bond precision:  C-C = 0.0037 A          Wavelength=0.71073

Cell:         a=8.4135(4)         b=13.0050(6)     c=16.7431(8)
              alpha=104.0055(12)  beta=92.4228(13) gamma=92.5966(13)
Temperature:  100 K
```

	Calculated	Reported
Volume	1773.02(15)	1773.02(15)
Space group	P -1	P -1
Hall group	-P 1	-P 1
Moiety formula	C52 H68 F6 N8 Na2 O18 P, F6 P, C4 H10 O, 1.5(C2 H3 N)	?
Sum formula	C59 H82.50 F12 N9.50 Na2 O19 P2	C59 H82.50 F12 N9.50 Na2 O19 P2
Mr	1564.76	1564.76
Dx,g cm-3	1.465	1.465
Z	1	1
Mu (mm-1)	0.181	0.181
F000	815.0	815.0
F000’	815.80	
h,k,lmax	10,15,19	10,15,19
Nref	6256	6246
Tmin,Tmax	0.980,0.984	0.910,0.980
Tmin’	0.950	

```
Correction method= # Reported T Limits: Tmin=0.910 Tmax=0.980
AbsCorr = MULTI-SCAN

Data completeness= 0.998          Theta(max)= 25.050

R(reflections)= 0.0509( 5083)     wR2(reflections)= 0.1328( 6246)

S = 1.046                  Npar= 610
```

The following ALERTS were generated. Each ALERT has the format
test-name_ALERT_alert-type_alert-level.
Click on the hyperlinks for more details of the test.

Alert level C

```
PLAT077_ALERT_4_C Unitcell Contains Non-integer Number of Atoms ..     Please Check
PLAT213_ALERT_2_C Atom O1              has ADP max/min Ratio .....        3.1 prolat
PLAT214_ALERT_2_C Atom F9A       (Anion/Solvent) ADP max/min Ratio        4.4 prolat
PLAT220_ALERT_2_C Non-Solvent  Resd 1  N   Ueq(max)/Ueq(min) Range        3.6 Ratio
PLAT220_ALERT_2_C Non-Solvent  Resd 1  O   Ueq(max)/Ueq(min) Range        4.8 Ratio
PLAT221_ALERT_2_C Solv./Anion  Resd 2  F   Ueq(max)/Ueq(min) Range        4.4 Ratio
PLAT250_ALERT_2_C Large U3/U1 Ratio for Average U(i,j) Tensor ....        2.2 Note
PLAT413_ALERT_2_C Short Inter XH3 .. XHn     H1E1     ..H13      .       2.13 Ang.
                                                -x,1-y,-z  =      2_565 Check
```

Alert level G

```
PLAT002_ALERT_2_G Number of Distance or Angle Restraints on AtSite         58 Note
PLAT003_ALERT_2_G Number of Uiso or Uij Restrained non-H Atoms ...          8 Report
PLAT005_ALERT_5_G No Embedded Refinement Details Found  in the CIF     Please Do !
PLAT242_ALERT_2_G Low    'MainMol' Ueq as Compared to Neighbors of         P1 Check
PLAT300_ALERT_4_G Atom Site Occupancy of P2A       Constrained at        0.3 Check
PLAT300_ALERT_4_G Atom Site Occupancy of F4A       Constrained at        0.3 Check
PLAT300_ALERT_4_G Atom Site Occupancy of F5A       Constrained at        0.3 Check
PLAT300_ALERT_4_G Atom Site Occupancy of F6A       Constrained at        0.3 Check
PLAT300_ALERT_4_G Atom Site Occupancy of F7A       Constrained at        0.3 Check
PLAT300_ALERT_4_G Atom Site Occupancy of F8A       Constrained at        0.3 Check
PLAT300_ALERT_4_G Atom Site Occupancy of F9A       Constrained at        0.3 Check
PLAT300_ALERT_4_G Atom Site Occupancy of P2B       Constrained at        0.2 Check
PLAT300_ALERT_4_G Atom Site Occupancy of F4B       Constrained at        0.2 Check
PLAT300_ALERT_4_G Atom Site Occupancy of F5B       Constrained at        0.2 Check
PLAT300_ALERT_4_G Atom Site Occupancy of F6B       Constrained at        0.2 Check
PLAT300_ALERT_4_G Atom Site Occupancy of F7B       Constrained at        0.2 Check
PLAT300_ALERT_4_G Atom Site Occupancy of F8B       Constrained at        0.2 Check
PLAT300_ALERT_4_G Atom Site Occupancy of F9B       Constrained at        0.2 Check
PLAT300_ALERT_4_G Atom Site Occupancy of O1E       Constrained at        0.5 Check
PLAT300_ALERT_4_G Atom Site Occupancy of C1E       Constrained at        0.5 Check
PLAT300_ALERT_4_G Atom Site Occupancy of C2E       Constrained at        0.5 Check
PLAT300_ALERT_4_G Atom Site Occupancy of C3E       Constrained at        0.5 Check
PLAT300_ALERT_4_G Atom Site Occupancy of C4E       Constrained at        0.5 Check
PLAT300_ALERT_4_G Atom Site Occupancy of H1E1      Constrained at        0.5 Check
PLAT300_ALERT_4_G Atom Site Occupancy of H1E2      Constrained at        0.5 Check
PLAT300_ALERT_4_G Atom Site Occupancy of H1E3      Constrained at        0.5 Check
PLAT300_ALERT_4_G Atom Site Occupancy of H2E1      Constrained at        0.5 Check
PLAT300_ALERT_4_G Atom Site Occupancy of H2E2      Constrained at        0.5 Check
PLAT300_ALERT_4_G Atom Site Occupancy of H3E1      Constrained at        0.5 Check
PLAT300_ALERT_4_G Atom Site Occupancy of H3E2      Constrained at        0.5 Check
PLAT300_ALERT_4_G Atom Site Occupancy of H4E1      Constrained at        0.5 Check
PLAT300_ALERT_4_G Atom Site Occupancy of H4E2      Constrained at        0.5 Check
PLAT300_ALERT_4_G Atom Site Occupancy of H4E3      Constrained at        0.5 Check
PLAT300_ALERT_4_G Atom Site Occupancy of N1S       Constrained at        0.5 Check
PLAT300_ALERT_4_G Atom Site Occupancy of C1S       Constrained at        0.5 Check
PLAT300_ALERT_4_G Atom Site Occupancy of C2S       Constrained at        0.5 Check
PLAT300_ALERT_4_G Atom Site Occupancy of H2S1      Constrained at        0.5 Check
PLAT300_ALERT_4_G Atom Site Occupancy of H2S2      Constrained at        0.5 Check
PLAT300_ALERT_4_G Atom Site Occupancy of H2S3      Constrained at        0.5 Check
PLAT300_ALERT_4_G Atom Site Occupancy of N1M       Constrained at       0.25 Check
PLAT300_ALERT_4_G Atom Site Occupancy of C1M       Constrained at       0.25 Check
PLAT300_ALERT_4_G Atom Site Occupancy of C2M       Constrained at       0.25 Check
```

```
PLAT300_ALERT_4_G Atom Site Occupancy of H1M1       Constrained at       0.25 Check
PLAT300_ALERT_4_G Atom Site Occupancy of H1M2       Constrained at       0.25 Check
PLAT300_ALERT_4_G Atom Site Occupancy of H1M3       Constrained at       0.25 Check
PLAT301_ALERT_3_G Main Residue  Disorder ..............(Resd  1  )        37% Note
PLAT302_ALERT_4_G Anion/Solvent/Minor-Residue Disorder (Resd  2  )       100% Note
PLAT302_ALERT_4_G Anion/Solvent/Minor-Residue Disorder (Resd  3  )       100% Note
PLAT302_ALERT_4_G Anion/Solvent/Minor-Residue Disorder (Resd  4  )       100% Note
PLAT302_ALERT_4_G Anion/Solvent/Minor-Residue Disorder (Resd  5  )       100% Note
PLAT302_ALERT_4_G Anion/Solvent/Minor-Residue Disorder (Resd  6  )       100% Note
PLAT304_ALERT_4_G Non-Integer Number of Atoms in ...... Resd  2          2.10 Check
PLAT304_ALERT_4_G Non-Integer Number of Atoms in ...... Resd  3          1.40 Check
PLAT304_ALERT_4_G Non-Integer Number of Atoms in ...... Resd  4          7.50 Check
PLAT304_ALERT_4_G Non-Integer Number of Atoms in ...... Resd  6          1.50 Check
PLAT720_ALERT_4_G Number of Unusual/Non-Standard Labels ..........         16 Note
PLAT779_ALERT_4_G Suspect or Irrelevant (Bond) Angle in CIF .... #         53 Check
              F1   -P1   -NA1     1.555   1.555   1.555             44.70 Deg.
PLAT779_ALERT_4_G Suspect or Irrelevant (Bond) Angle in CIF .... #         60 Check
              F1   -P1   -NA1     2.666   1.555   2.666             44.70 Deg.
PLAT790_ALERT_4_G Centre of Gravity not Within Unit Cell: Resd.  #          5 Note
              C2 H3 N
PLAT811_ALERT_5_G No ADDSYM Analysis: Too Many Excluded Atoms ....          ! Info
PLAT860_ALERT_3_G Number of Least-Squares Restraints .............        175 Note
PLAT883_ALERT_1_G No Info/Value for _atom_sites_solution_primary .     Please Do !
```

```
   0 ALERT level A = Most likely a serious problem - resolve or explain
   0 ALERT level B = A potentially serious problem, consider carefully
   8 ALERT level C = Check. Ensure it is not caused by an omission or oversight
  62 ALERT level G = General information/check it is not something unexpected

   1 ALERT type 1 CIF construction/syntax error, inconsistent or missing data
  10 ALERT type 2 Indicator that the structure model may be wrong or deficient
   2 ALERT type 3 Indicator that the structure quality may be low
  55 ALERT type 4 Improvement, methodology, query or suggestion
   2 ALERT type 5 Informative message, check
```

It is advisable to attempt to resolve as many as possible of the alerts in all categories. Often the minor alerts point to easily fixed oversights, errors and omissions in your CIF or refinement strategy, so attention to these fine details can be worthwhile. In order to resolve some of the more serious problems it may be necessary to carry out additional measurements or structure refinements. However, the purpose of your study may justify the reported deviations and the more serious of these should normally be commented upon in the discussion or experimental section of a paper or in the "special_details" fields of the CIF. checkCIF was carefully designed to identify outliers and unusual parameters, but every test has its limitations and alerts that are not important in a particular case may appear. Conversely, the absence of alerts does not guarantee there are no aspects of the results needing attention. It is up to the individual to critically assess their own results and, if necessary, seek expert advice.

Publication of your CIF in IUCr journals

A basic structural check has been run on your CIF. These basic checks will be run on all CIFs submitted for publication in IUCr journals (*Acta Crystallographica*, *Journal of Applied Crystallography*, *Journal of Synchrotron Radiation*); however, if you intend to submit to *Acta Crystallographica Section C* or *E* or *IUCrData*, you should make sure that full publication checks are run on the final version of your CIF prior to submission.

Publication of your CIF in other journals

Please refer to the *Notes for Authors* of the relevant journal for any special instructions relating to CIF submission.

PLATON version of 03/05/2019; check.def file version of 29/04/2019

Datablock complex_1_NaPF6 - ellipsoid plot

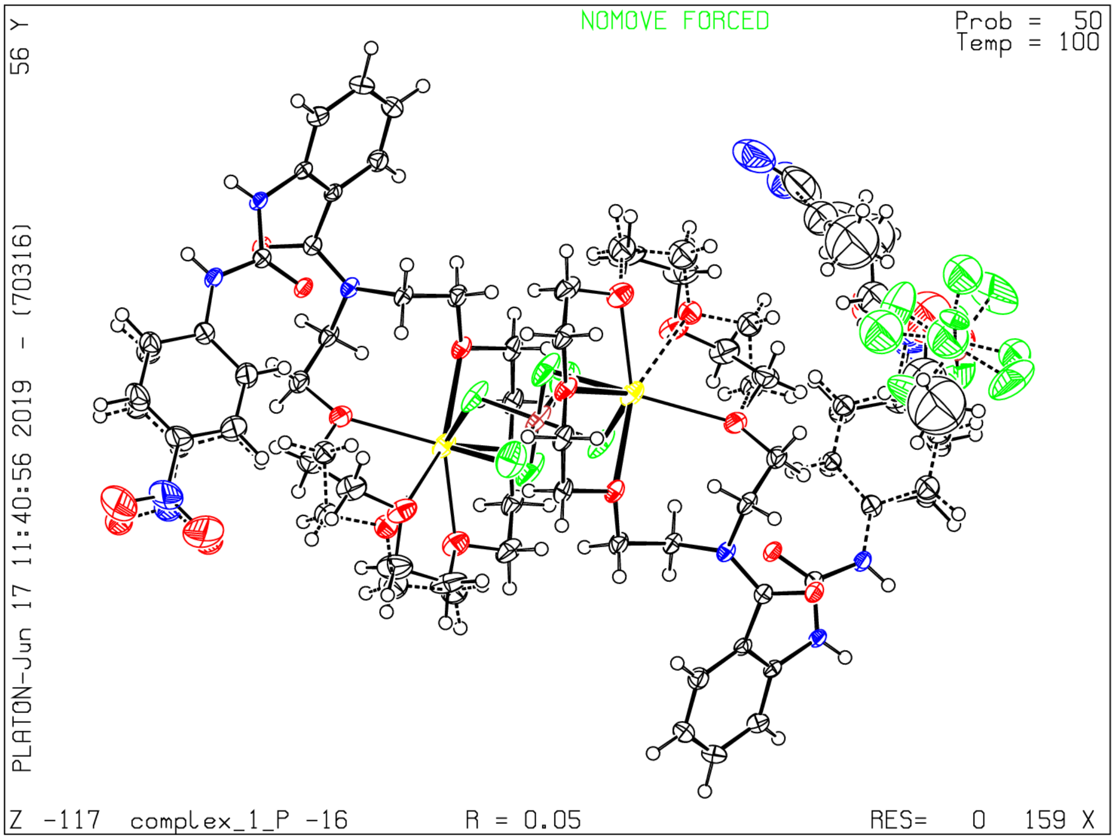

Supplement: Supplementary file 1 [file molecules-24-02990-s001.zip › checkcif_complex_1_NaPF6.pdf]
