# Supplementary material for: The Effect of Substitution Pattern on Binding Ability in Regioisomeric Ion Pair Receptors Based on an Aminobenzoic Platform
Source: Molecules. 2019 Aug 18;24(16):2990. doi: 10.3390/molecules24162990 (PMC6720599; doi:10.3390/molecules24162990)

checkCIF/PLATON report

You have not supplied any structure factors. As a result the full set of tests cannot be run.

THIS REPORT IS FOR GUIDANCE ONLY. IF USED AS PART OF A REVIEW PROCEDURE FOR PUBLICATION, IT SHOULD NOT REPLACE THE EXPERTISE OF AN EXPERIENCED CRYSTALLOGRAPHIC REFEREE.

No syntax errors found. CIF dictionary Interpreting this report

Datablock: complex_2_NaBr

```
Bond precision:  C-C = 0.0032 A          Wavelength=0.71073

Cell:          a=12.7282(10)      b=13.6326(11)     c=14.5482(12)
               alpha=106.806(2)   beta=113.890(2)   gamma=102.082(2)
Temperature:   100 K
```

	Calculated	Reported
Volume	2050.1(3)	2050.1(3)
Space group	P -1	P -1
Hall group	-P 1	-P 1
Moiety formula	C30 H46 N4 Na O11 S2, 0.683(C4 H10 O), 0.317(C2 H6 O S), Br	?
Sum formula	C33.37 H54.73 Br N4 Na O12 S2.32	C33.36 H54.73 Br N4 Na O12 S2.32
Mr	881.14	881.04
Dx,g cm-3	1.427	1.427
Z	2	2
Mu (mm-1)	1.194	1.193
F000	924.0	924.0
F000’	924.50	
h,k,lmax	15,16,17	15,16,17
Nref	7252	7246
Tmin,Tmax	0.688,0.827	0.770,0.830
Tmin’	0.674	

```
Correction method= # Reported T Limits: Tmin=0.770 Tmax=0.830
AbsCorr = MULTI-SCAN

Data completeness= 0.999          Theta(max)= 25.050

R(reflections)= 0.0286( 6531)     wR2(reflections)= 0.0688( 7246)

S = 1.048                   Npar= 543
```

The following ALERTS were generated. Each ALERT has the format
test-name_ALERT_alert-type_alert-level.
Click on the hyperlinks for more details of the test.

Alert level C

```
PLAT041_ALERT_1_C Calc. and Reported SumFormula    Strings  Differ     Please Check
PLAT077_ALERT_4_C Unitcell Contains Non-integer Number of Atoms ..     Please Check
```

Alert level G

```
PLAT002_ALERT_2_G Number of Distance or Angle Restraints on AtSite          9 Note
PLAT003_ALERT_2_G Number of Uiso or Uij Restrained non-H Atoms ...          6 Report
PLAT005_ALERT_5_G No Embedded Refinement Details Found  in the CIF     Please Do !
PLAT154_ALERT_1_G The s.u.'s on the Cell Angles are Equal ..(Note)      0.002 Degree
PLAT301_ALERT_3_G Main Residue  Disorder ..............(Resd  1  )         8% Note
PLAT302_ALERT_4_G Anion/Solvent/Minor-Residue Disorder (Resd  2  )       100% Note
PLAT302_ALERT_4_G Anion/Solvent/Minor-Residue Disorder (Resd  3  )       100% Note
PLAT302_ALERT_4_G Anion/Solvent/Minor-Residue Disorder (Resd  4  )       100% Note
PLAT304_ALERT_4_G Non-Integer Number of Atoms in ...... Resd  2         10.24 Check
PLAT304_ALERT_4_G Non-Integer Number of Atoms in ...... Resd  3          1.59 Check
PLAT304_ALERT_4_G Non-Integer Number of Atoms in ...... Resd  4          1.59 Check
PLAT398_ALERT_2_G Deviating  C-O-C    Angle From 120 for O1S            107.3 Degree
PLAT432_ALERT_2_G Short Inter X...Y Contact  S1T      ..C10              3.21 Ang.
                                                      x,y,z  =      1_555 Check
PLAT720_ALERT_4_G Number of Unusual/Non-Standard Labels ..........         40 Note
PLAT790_ALERT_4_G Centre of Gravity not Within Unit Cell: Resd.  #          2 Note
              C4 H10 O
PLAT790_ALERT_4_G Centre of Gravity not Within Unit Cell: Resd.  #          3 Note
              C2 H6 O S
PLAT790_ALERT_4_G Centre of Gravity not Within Unit Cell: Resd.  #          4 Note
              C2 H6 O S
PLAT860_ALERT_3_G Number of Least-Squares Restraints .............         19 Note
PLAT883_ALERT_1_G No Info/Value for _atom_sites_solution_primary .     Please Do !
```

0 **ALERT level A** = Most likely a serious problem - resolve or explain
0 **ALERT level B** = A potentially serious problem, consider carefully
2 **ALERT level C** = Check. Ensure it is not caused by an omission or oversight
19 **ALERT level G** = General information/check it is not something unexpected

3 ALERT type 1 CIF construction/syntax error, inconsistent or missing data
4 ALERT type 2 Indicator that the structure model may be wrong or deficient
2 ALERT type 3 Indicator that the structure quality may be low
11 ALERT type 4 Improvement, methodology, query or suggestion
1 ALERT type 5 Informative message, check

It is advisable to attempt to resolve as many as possible of the alerts in all categories. Often the minor alerts point to easily fixed oversights, errors and omissions in your CIF or refinement strategy, so attention to these fine details can be worthwhile. In order to resolve some of the more serious problems it may be necessary to carry out additional measurements or structure refinements. However, the purpose of your study may justify the reported deviations and the more serious of these should normally be commented upon in the discussion or experimental section of a paper or in the "special_details" fields of the CIF. checkCIF was carefully designed to identify outliers and unusual parameters, but every test has its limitations and alerts that are not important in a particular case may appear. Conversely, the absence of alerts does not guarantee there are no aspects of the results needing attention. It is up to the individual to critically assess their own results and, if necessary, seek expert advice.

Publication of your CIF in IUCr journals

A basic structural check has been run on your CIF. These basic checks will be run on all CIFs submitted for publication in IUCr journals (*Acta Crystallographica*, *Journal of Applied Crystallography*, *Journal of Synchrotron Radiation*); however, if you intend to submit to *Acta Crystallographica Section C* or *E* or *IUCrData*, you should make sure that full publication checks are run on the final version of your CIF prior to submission.

Publication of your CIF in other journals

Please refer to the *Notes for Authors* of the relevant journal for any special instructions relating to CIF submission.

PLATON version of 03/05/2019; check.def file version of 29/04/2019

Datablock complex_2_NaBr - ellipsoid plot

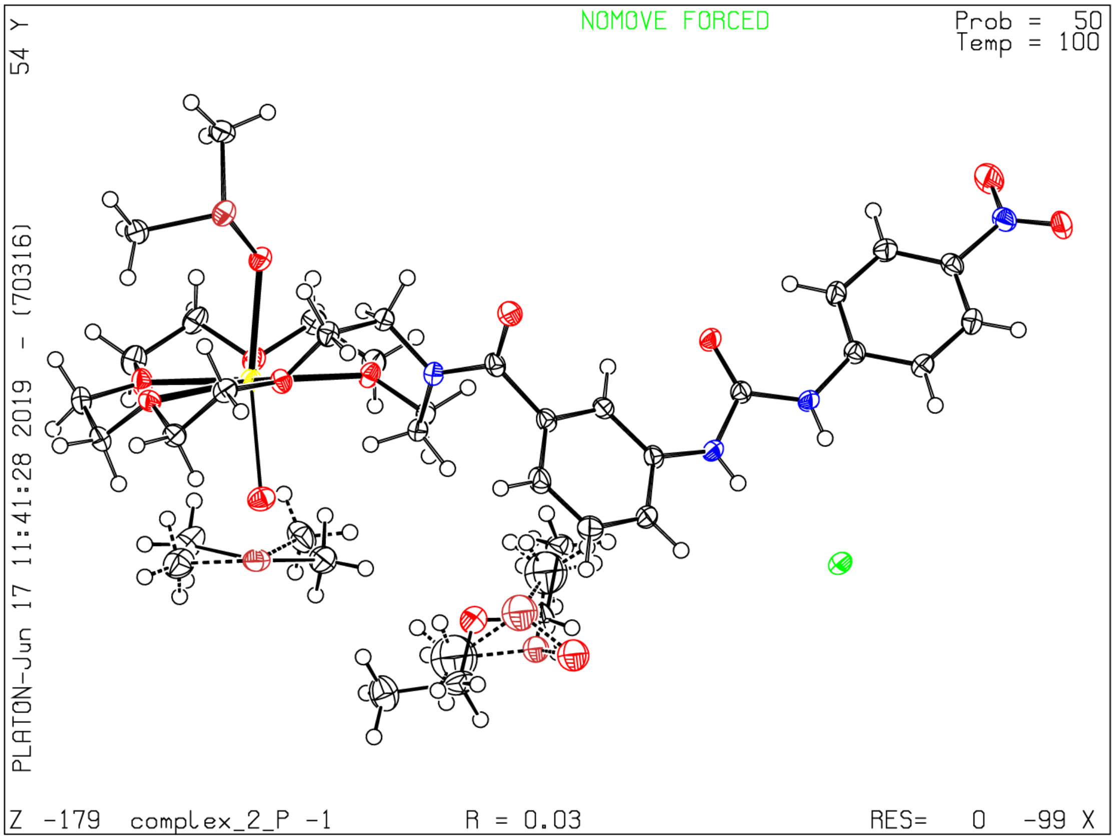

Supplement: Supplementary file 1 [file molecules-24-02990-s001.zip › checkcif_complex_2_NaBr.pdf]
