# Supplementary material for: The Effect of Substitution Pattern on Binding Ability in Regioisomeric Ion Pair Receptors Based on an Aminobenzoic Platform
Source: Molecules. 2019 Aug 18;24(16):2990. doi: 10.3390/molecules24162990 (PMC6720599; doi:10.3390/molecules24162990)

checkCIF/PLATON report

You have not supplied any structure factors. As a result the full set of tests cannot be run.

THIS REPORT IS FOR GUIDANCE ONLY. IF USED AS PART OF A REVIEW PROCEDURE FOR PUBLICATION, IT SHOULD NOT REPLACE THE EXPERTISE OF AN EXPERIENCED CRYSTALLOGRAPHIC REFEREE.

No syntax errors found. CIF dictionary Interpreting this report

Datablock: complex_3_NaBr

Bond precision: C-C = 0.0076 A Wavelength=0.71073

Cell:	a=15.488(2)	b=14.836(2)	c=55.956(9)
	alpha=90	beta=96.5751(19)	gamma=90
Temperature:	100 K		

	Calculated	Reported
Volume	12773(3)	12773(3)
Space group	P 21/n	P 1 21/n 1
Hall group	-P 2yn	-P 2yn
Moiety formula	C27 H38 Br N4 Na O10	?
Sum formula	C27 H38 Br N4 Na O10	C27 H38 Br N4 Na O10
Mr	681.50	681.51
Dx,g cm-3	1.418	1.418
Z	16	16
Mu (mm-1)	1.360	1.360
F000	5664.0	5664.0
F000’	5662.56	
h,k,lmax	18,17,66	18,17,66
Nref	22641	22597
Tmin,Tmax	0.753,0.880	0.730,0.880
Tmin’	0.600	

Correction method= # Reported T Limits: Tmin=0.730 Tmax=0.880
AbsCorr = MULTI-SCAN

Data completeness= 0.998 Theta(max)= 25.050

R(reflections)= 0.0680(17759) wR2(reflections)= 0.1408(22597)

S = 1.346 Npar= 1593

The following ALERTS were generated. Each ALERT has the format
test-name_ALERT_alert-type_alert-level.
Click on the hyperlinks for more details of the test.

Alert level C

```
PLAT213_ALERT_2_C Atom N3B            has ADP max/min Ratio .....        3.2 prolat
PLAT213_ALERT_2_C Atom C11B           has ADP max/min Ratio .....        3.6 oblate
PLAT213_ALERT_2_C Atom C13B           has ADP max/min Ratio .....        3.5 oblate
PLAT213_ALERT_2_C Atom C11D           has ADP max/min Ratio .....        3.2 prolat
PLAT220_ALERT_2_C Non-Solvent  Resd 2  C   Ueq(max)/Ueq(min) Range        4.2 Ratio
PLAT220_ALERT_2_C Non-Solvent  Resd 3  C   Ueq(max)/Ueq(min) Range        5.2 Ratio
PLAT222_ALERT_3_C Non-Solv.  Resd 2  H   Uiso(max)/Uiso(min) Range        4.1 Ratio
PLAT222_ALERT_3_C Non-Solv.  Resd 3  H   Uiso(max)/Uiso(min) Range        4.7 Ratio
PLAT230_ALERT_2_C Hirshfeld Test Diff for    O7A      --C20A     .        5.2 s.u.
PLAT230_ALERT_2_C Hirshfeld Test Diff for    C21A     --C22A     .        6.8 s.u.
PLAT230_ALERT_2_C Hirshfeld Test Diff for    N3B      --C7B      .        5.5 s.u.
PLAT230_ALERT_2_C Hirshfeld Test Diff for    O3C      --C7C      .        6.3 s.u.
PLAT230_ALERT_2_C Hirshfeld Test Diff for    C4C      --C5C      .        5.8 s.u.
PLAT230_ALERT_2_C Hirshfeld Test Diff for    C11C     --C12C     .        5.2 s.u.
PLAT230_ALERT_2_C Hirshfeld Test Diff for    O3D      --C7D      .        5.7 s.u.
PLAT341_ALERT_3_C Low Bond Precision on  C-C Bonds ...............    0.00763 Ang.
PLAT416_ALERT_2_C Short Intra D-H..H-D       H2ND     ..H3ND     .       1.97 Ang.
                                                      x,y,z  =      1_555 Check
PLAT601_ALERT_2_C Structure Contains Solvent Accessible VOIDS of .         56 Ang**3
```

Alert level G

```
PLAT002_ALERT_2_G Number of Distance or Angle Restraints on AtSite         24 Note
PLAT003_ALERT_2_G Number of Uiso or Uij Restrained non-H Atoms ...          4 Report
PLAT005_ALERT_5_G No Embedded Refinement Details Found  in the CIF     Please Do !
PLAT083_ALERT_2_G SHELXL Second Parameter in WGHT  Unusually Large      58.10 Why ?
PLAT432_ALERT_2_G Short Inter X...Y Contact  O1D      ..C25B             2.97 Ang.
                                          1/2-x,1/2+y,3/2-z  =      2_556 Check
PLAT720_ALERT_4_G Number of Unusual/Non-Standard Labels ..........         12 Note
PLAT860_ALERT_3_G Number of Least-Squares Restraints .............         36 Note
PLAT883_ALERT_1_G No Info/Value for _atom_sites_solution_primary .     Please Do !
```

```
   0 ALERT level A = Most likely a serious problem - resolve or explain
   0 ALERT level B = A potentially serious problem, consider carefully
  18 ALERT level C = Check. Ensure it is not caused by an omission or oversight
   8 ALERT level G = General information/check it is not something unexpected

   1 ALERT type 1 CIF construction/syntax error, inconsistent or missing data
  19 ALERT type 2 Indicator that the structure model may be wrong or deficient
   4 ALERT type 3 Indicator that the structure quality may be low
   1 ALERT type 4 Improvement, methodology, query or suggestion
   1 ALERT type 5 Informative message, check
```

It is advisable to attempt to resolve as many as possible of the alerts in all categories. Often the minor alerts point to easily fixed oversights, errors and omissions in your CIF or refinement strategy, so attention to these fine details can be worthwhile. In order to resolve some of the more serious problems it may be necessary to carry out additional measurements or structure refinements. However, the purpose of your study may justify the reported deviations and the more serious of these should normally be commented upon in the discussion or experimental section of a paper or in the "special_details" fields of the CIF. checkCIF was carefully designed to identify outliers and unusual parameters, but every test has its limitations and alerts that are not important in a particular case may appear. Conversely, the absence of alerts does not guarantee there are no aspects of the results needing attention. It is up to the individual to critically assess their own results and, if necessary, seek expert advice.

Publication of your CIF in IUCr journals

A basic structural check has been run on your CIF. These basic checks will be run on all CIFs submitted for publication in IUCr journals (*Acta Crystallographica*, *Journal of Applied Crystallography*, *Journal of Synchrotron Radiation*); however, if you intend to submit to *Acta Crystallographica Section C* or *E* or *IUCrData*, you should make sure that full publication checks are run on the final version of your CIF prior to submission.

Publication of your CIF in other journals

Please refer to the *Notes for Authors* of the relevant journal for any special instructions relating to CIF submission.

PLATON version of 03/05/2019; check.def file version of 29/04/2019

Datablock complex_3_NaBr - ellipsoid plot

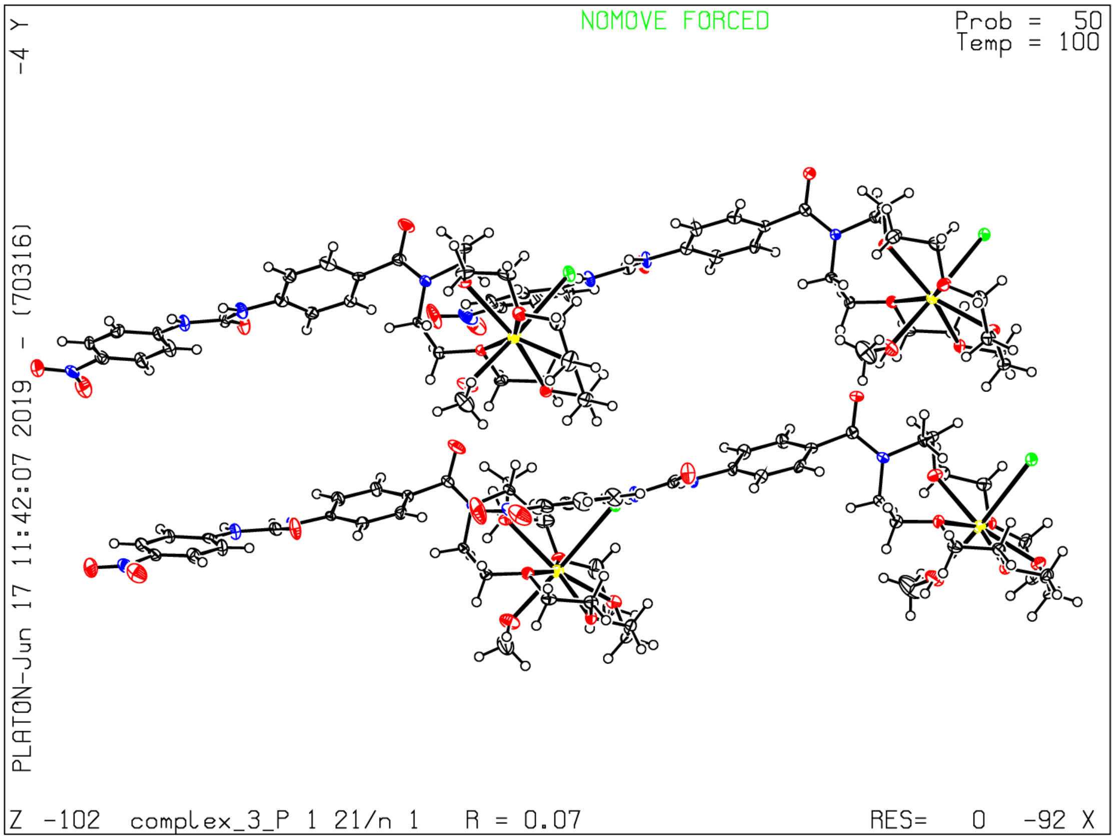

Supplement: Supplementary file 1 [file molecules-24-02990-s001.zip › checkcif_complex_3_NaBr.pdf]
