# Supplementary material for: The Effect of Substitution Pattern on Binding Ability in Regioisomeric Ion Pair Receptors Based on an Aminobenzoic Platform
Source: Molecules. 2019 Aug 18;24(16):2990. doi: 10.3390/molecules24162990 (PMC6720599; doi:10.3390/molecules24162990)

checkCIF/PLATON report

You have not supplied any structure factors. As a result the full set of tests cannot be run.

THIS REPORT IS FOR GUIDANCE ONLY. IF USED AS PART OF A REVIEW PROCEDURE FOR PUBLICATION, IT SHOULD NOT REPLACE THE EXPERTISE OF AN EXPERIENCED CRYSTALLOGRAPHIC REFEREE.

No syntax errors found. CIF dictionary Interpreting this report

Datablock: receptor_1

Bond precision: C-C = 0.0037 A Wavelength=0.71073

Cell:	a=13.9190(9)	b=15.511(1)	c=16.3591(10)
	alpha=71.369(2)	beta=73.035(2)	gamma=67.080(2)
Temperature:	100 K		

	Calculated	Reported
Volume	3025.4(3)	3025.4(3)
Space group	P -1	P -1
Hall group	-P 1	-P 1
Moiety formula	C26 H34 N4 O9, 0.915(C H Cl3)	?
Sum formula	C26.92 H34.92 Cl2.75 N4 O9	C53.83 H69.83 Cl5.49 N8 O18
Mr	655.79	1311.58
Dx,g cm-3	1.440	1.440
Z	4	2
Mu (mm-1)	0.339	0.339
F000	1372.3	1372.0
F000’	1374.55	
h,k,lmax	16,18,19	16,18,19
Nref	10741	10732
Tmin,Tmax	0.907,0.969	0.930,0.970
Tmin’	0.889	

Correction method= # Reported T Limits: Tmin=0.930 Tmax=0.970
AbsCorr = MULTI-SCAN

Data completeness= 0.999 Theta(max)= 25.050

R(reflections)= 0.0529(8815) wR2(reflections)= 0.1349(10732)

S = 1.021 Npar= 949

The following ALERTS were generated. Each ALERT has the format
test-name_ALERT_alert-type_alert-level.
Click on the hyperlinks for more details of the test.

Alert level C

```
PLAT213_ALERT_2_C Atom O8D            has ADP max/min Ratio .....        3.5 prolat
PLAT213_ALERT_2_C Atom C16B           has ADP max/min Ratio .....        3.1 prolat
PLAT220_ALERT_2_C Non-Solvent  Resd 1  C   Ueq(max)/Ueq(min) Range       3.8 Ratio
PLAT220_ALERT_2_C Non-Solvent  Resd 1  O   Ueq(max)/Ueq(min) Range       3.8 Ratio
PLAT336_ALERT_2_C Long Bond Distance for ..... C1SB     -Cl2B          1.860 Ang.
```

Alert level G

```
PLAT002_ALERT_2_G Number of Distance or Angle Restraints on AtSite        62 Note
PLAT003_ALERT_2_G Number of Uiso or Uij Restrained non-H Atoms ...         4 Report
PLAT005_ALERT_5_G No Embedded Refinement Details Found  in the CIF     Please Do !
PLAT045_ALERT_1_G Calculated and Reported Z Differ by a Factor ...      2.00 Check
PLAT068_ALERT_1_G Reported F000 Differs from Calcd (or Missing)...    Please Check
PLAT154_ALERT_1_G The s.u.'s on the Cell Angles are Equal ..(Note)     0.002 Degree
PLAT230_ALERT_2_G Hirshfeld Test Diff for    O8B      --C23B     .       9.0 s.u.
PLAT230_ALERT_2_G Hirshfeld Test Diff for    C23B     --C24B     .       5.4 s.u.
PLAT300_ALERT_4_G Atom Site Occupancy of O3A         Constrained at      0.5 Check
PLAT300_ALERT_4_G Atom Site Occupancy of O3C         Constrained at      0.5 Check
PLAT300_ALERT_4_G Atom Site Occupancy of Cl1C        Constrained at      0.5 Check
PLAT300_ALERT_4_G Atom Site Occupancy of Cl2C        Constrained at      0.5 Check
PLAT300_ALERT_4_G Atom Site Occupancy of Cl3C        Constrained at      0.5 Check
PLAT300_ALERT_4_G Atom Site Occupancy of C1SC        Constrained at      0.5 Check
PLAT300_ALERT_4_G Atom Site Occupancy of H1SC        Constrained at      0.5 Check
PLAT300_ALERT_4_G Atom Site Occupancy of Cl1D        Constrained at     0.23 Check
PLAT300_ALERT_4_G Atom Site Occupancy of Cl2D        Constrained at     0.23 Check
PLAT300_ALERT_4_G Atom Site Occupancy of Cl3D        Constrained at     0.23 Check
PLAT300_ALERT_4_G Atom Site Occupancy of C1SD        Constrained at     0.23 Check
PLAT300_ALERT_4_G Atom Site Occupancy of H1SD        Constrained at     0.23 Check
PLAT300_ALERT_4_G Atom Site Occupancy of Cl1E        Constrained at      0.1 Check
PLAT300_ALERT_4_G Atom Site Occupancy of Cl2E        Constrained at      0.1 Check
PLAT300_ALERT_4_G Atom Site Occupancy of Cl3E        Constrained at      0.1 Check
PLAT300_ALERT_4_G Atom Site Occupancy of C1SE        Constrained at      0.1 Check
PLAT300_ALERT_4_G Atom Site Occupancy of H1SE        Constrained at      0.1 Check
PLAT301_ALERT_3_G Main Residue  Disorder .............(Resd  1  )        36% Note
PLAT301_ALERT_3_G Main Residue  Disorder .............(Resd  2  )         3% Note
PLAT302_ALERT_4_G Anion/Solvent/Minor-Residue Disorder (Resd  3  )      100% Note
PLAT302_ALERT_4_G Anion/Solvent/Minor-Residue Disorder (Resd  4  )      100% Note
PLAT302_ALERT_4_G Anion/Solvent/Minor-Residue Disorder (Resd  5  )      100% Note
PLAT302_ALERT_4_G Anion/Solvent/Minor-Residue Disorder (Resd  6  )      100% Note
PLAT302_ALERT_4_G Anion/Solvent/Minor-Residue Disorder (Resd  7  )      100% Note
PLAT304_ALERT_4_G Non-Integer Number of Atoms in ...... Resd  3         3.16 Check
PLAT304_ALERT_4_G Non-Integer Number of Atoms in ...... Resd  4         2.50 Check
PLAT304_ALERT_4_G Non-Integer Number of Atoms in ...... Resd  5         1.84 Check
PLAT304_ALERT_4_G Non-Integer Number of Atoms in ...... Resd  6         1.15 Check
PLAT304_ALERT_4_G Non-Integer Number of Atoms in ...... Resd  7         0.50 Check
PLAT395_ALERT_2_G Deviating  X-O-Y    Angle From 120 for O3A            76.5 Degree
PLAT395_ALERT_2_G Deviating  X-O-Y    Angle From 120 for O3C            76.9 Degree
PLAT398_ALERT_2_G Deviating  C-O-C    Angle From 120 for O8B            80.6 Degree
PLAT398_ALERT_2_G Deviating  C-O-C    Angle From 120 for O8D           154.9 Degree
PLAT720_ALERT_4_G Number of Unusual/Non-Standard Labels ..........        14 Note
PLAT773_ALERT_2_G Check long C-C Bond in CIF: C22B     --C23B           1.94 Ang.
PLAT779_ALERT_4_G Suspect or Irrelevant (Bond) Angle in CIF .... #        33 Check
              O3C  -C7A  -O3A     1.555   1.555   1.555            26.60 Deg.
PLAT811_ALERT_5_G No ADDSYM Analysis: Too Many Excluded Atoms ....         ! Info
PLAT860_ALERT_3_G Number of Least-Squares Restraints ............        160 Note
```

```
PLAT883_ALERT_1_G No Info/Value for _atom_sites_solution_primary .     Please Do !
```

```
   0 ALERT level A = Most likely a serious problem - resolve or explain
   0 ALERT level B = A potentially serious problem, consider carefully
   5 ALERT level C = Check. Ensure it is not caused by an omission or oversight
  47 ALERT level G = General information/check it is not something unexpected

   4 ALERT type 1 CIF construction/syntax error, inconsistent or missing data
  14 ALERT type 2 Indicator that the structure model may be wrong or deficient
   3 ALERT type 3 Indicator that the structure quality may be low
  29 ALERT type 4 Improvement, methodology, query or suggestion
   2 ALERT type 5 Informative message, check
```

It is advisable to attempt to resolve as many as possible of the alerts in all categories. Often the minor alerts point to easily fixed oversights, errors and omissions in your CIF or refinement strategy, so attention to these fine details can be worthwhile. In order to resolve some of the more serious problems it may be necessary to carry out additional measurements or structure refinements. However, the purpose of your study may justify the reported deviations and the more serious of these should normally be commented upon in the discussion or experimental section of a paper or in the "special_details" fields of the CIF. checkCIF was carefully designed to identify outliers and unusual parameters, but every test has its limitations and alerts that are not important in a particular case may appear. Conversely, the absence of alerts does not guarantee there are no aspects of the results needing attention. It is up to the individual to critically assess their own results and, if necessary, seek expert advice.

Publication of your CIF in IUCr journals

A basic structural check has been run on your CIF. These basic checks will be run on all CIFs submitted for publication in IUCr journals (*Acta Crystallographica*, *Journal of Applied Crystallography*, *Journal of Synchrotron Radiation*); however, if you intend to submit to *Acta Crystallographica Section C* or *E* or *IUCrData*, you should make sure that full publication checks are run on the final version of your CIF prior to submission.

Publication of your CIF in other journals

Please refer to the *Notes for Authors* of the relevant journal for any special instructions relating to CIF submission.

PLATON version of 03/05/2019; check.def file version of 29/04/2019

Datablock receptor_1 - ellipsoid plot

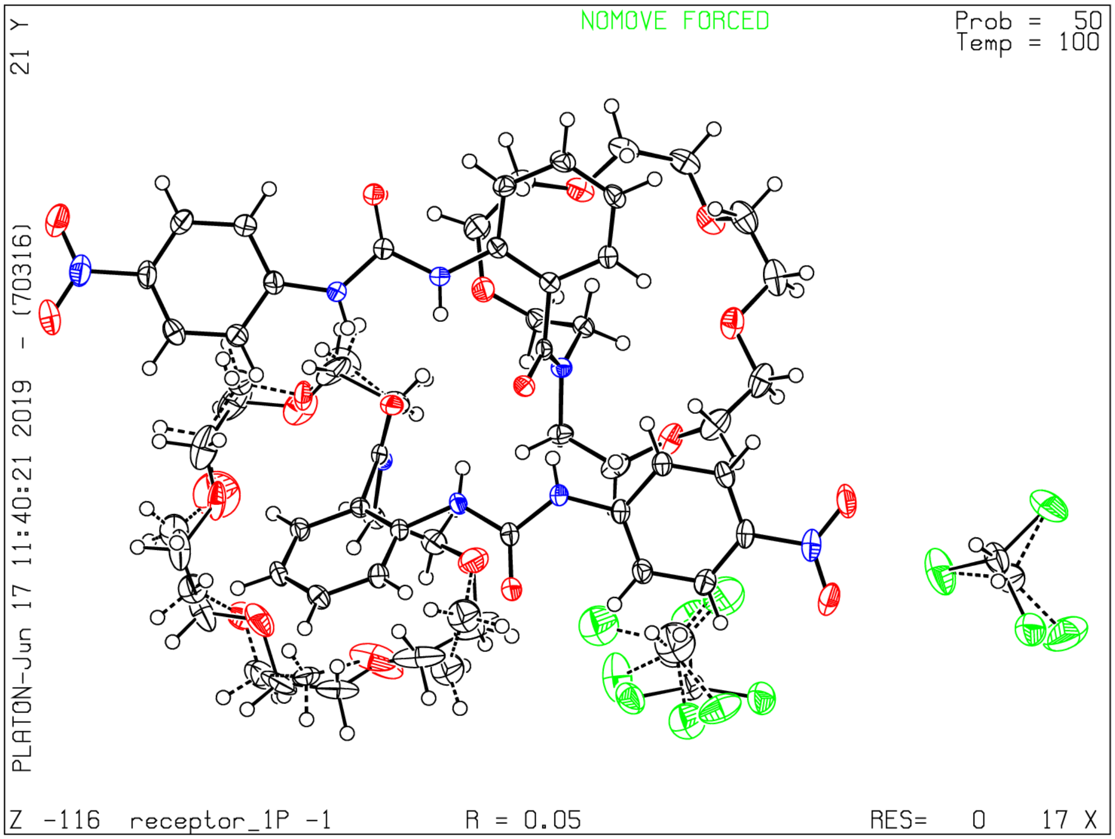

Supplement: Supplementary file 1 [file molecules-24-02990-s001.zip › checkcif_receptor_1.pdf]
