# Supplementary material for: The Effect of Substitution Pattern on Binding Ability in Regioisomeric Ion Pair Receptors Based on an Aminobenzoic Platform
Source: Molecules. 2019 Aug 18;24(16):2990. doi: 10.3390/molecules24162990 (PMC6720599; doi:10.3390/molecules24162990)
Supplement: Supplementary file 1 [file molecules-24-02990-s001.zip › Molecules_ESI_JR_Rev.pdf]

The effect of substitution pattern on binding ability in regioisomeric ion pair receptors based on aminobenzoic platform

Damian Jagleniec[a], Krzysztof Ziach[a], Kajetan Dąbrowa[b] and Jan Romański[a,*]

[a]Faculty of Chemistry, University of Warsaw, Pasteura 1, 02-093 Warsaw, Poland
[b]Institute of Organic Chemistry, Polish Academy of Sciences, Kasprzaka 44/52, 01-224 Warsaw, Poland

*jarom@chem.uw.edu.pl

TABLE OF CONTENTS

1. General 1
2. UV-vis measurements 2
3. NMR measurements 6
4. Crystal data 11
5. NMR spectra 22

GENERAL INFORMATION

Unless specifically indicated, all other chemicals and reagents used in this study were purchased from commercial sources and used as received. If necessary purification of products was performed using column chromatography on silica gel (Merck Kieselgel 60, 230-400 mesh) with mixtures of chloroform/methanol. Thin-layer chromatography (TLC) was performed on silica gel plates (Merck Kieselgel 60 F254).

^{1}H and ^{13}C NMR spectra used in the characterization of products were recorded on Bruker 300 spectrometer using a residual protonated solvent as internal standard.

UV-vis analyses were performed using Thermo Spectronic Unicam UV500 Spectrophotometer.

UV-vis titration experiments

The UV-Vis titration was performed using Thermo Spectronic Unicam UV500 Spectrophotometer at 298K in acetonitrile. In each case, a 2500 μL of freshly prepared 2.93×10^{-5} M solution of receptor was added to a cuvette and small aliquots of TBAX, containing constant concentration of the receptor, were added and a spectrum was acquired after each addition. In the case of ion pair titration receptor was firstly pretreated with one equivalent of $NaClO_4$ (refers to receptor).The resulting titration data were analyzed using BindFit (v0.5) package, available online at http://supramolecular.org

Fig. S1: UV-Vis titration of receptor **1** with TBACl and selected binding isotherms.

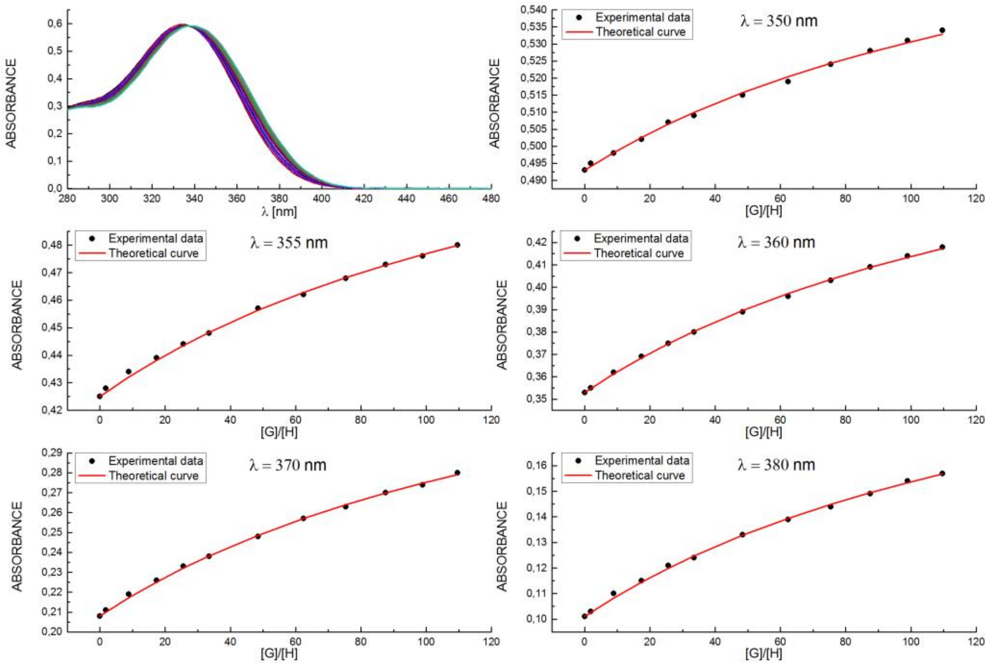

Fig. S2: UV-Vis titration of receptor **1** with TBACl in the presence of 1 equivalent of $NaClO_4$ and selected binding isotherms.

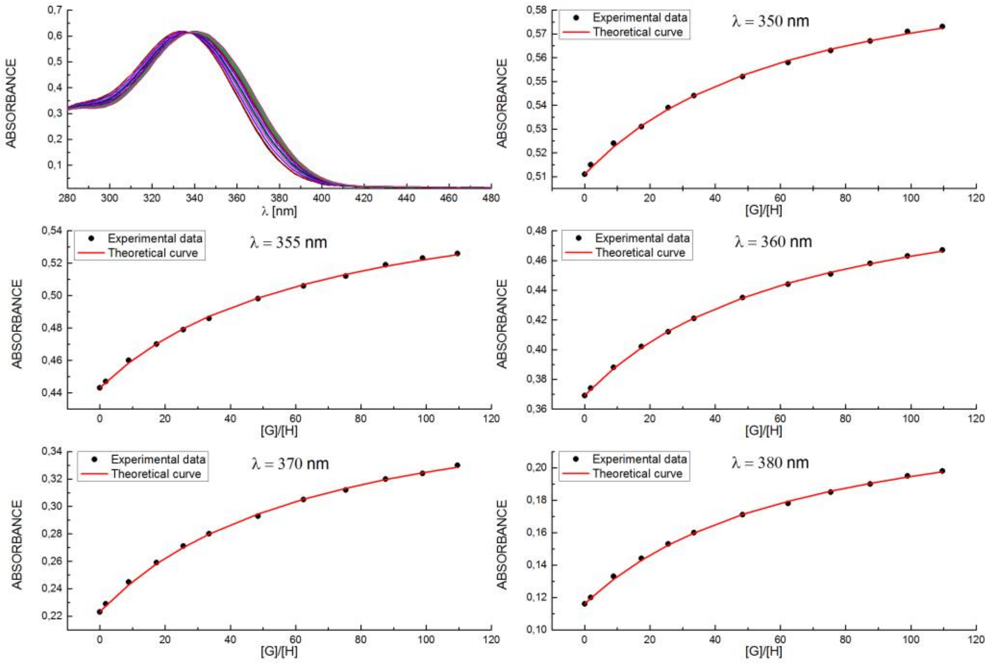

Fig. S3: UV-Vis titration of receptor **2** with TBACl and selected binding isotherms.

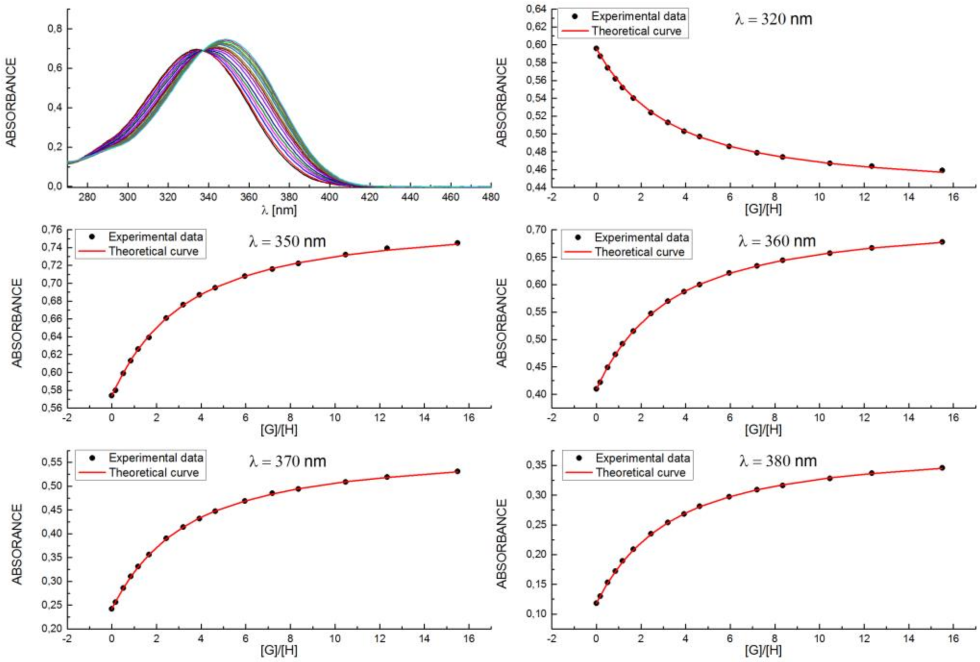

Fig. S4: UV-Vis titration of receptor **1** with TBACl in the presence of 1 equivalent of $NaClO_4$ and selected binding isotherms.

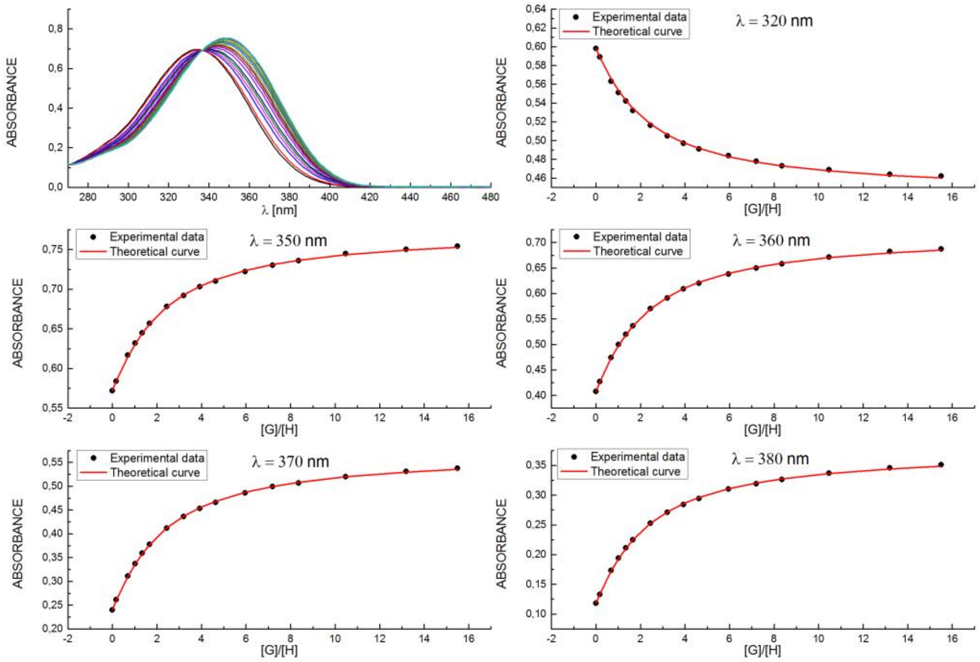

Fig. S5: UV-Vis titration of receptor **3** with TBACl and selected binding isotherms.

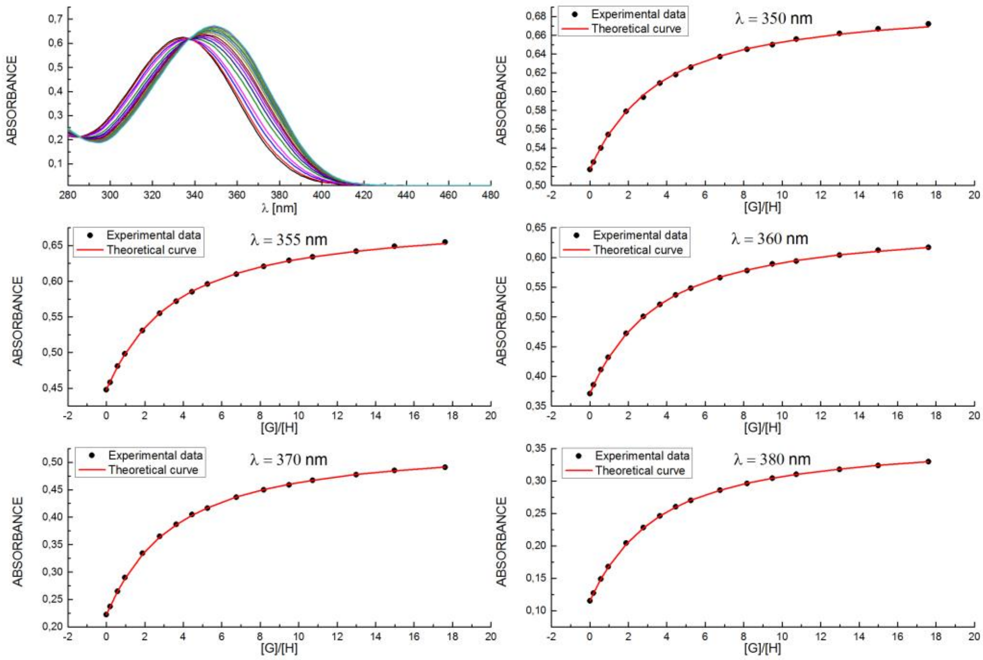

Fig. S6: UV-Vis titration of receptor **3** with TBACl in the presence of 1 equivalent of $NaClO_4$ and selected binding isotherms.

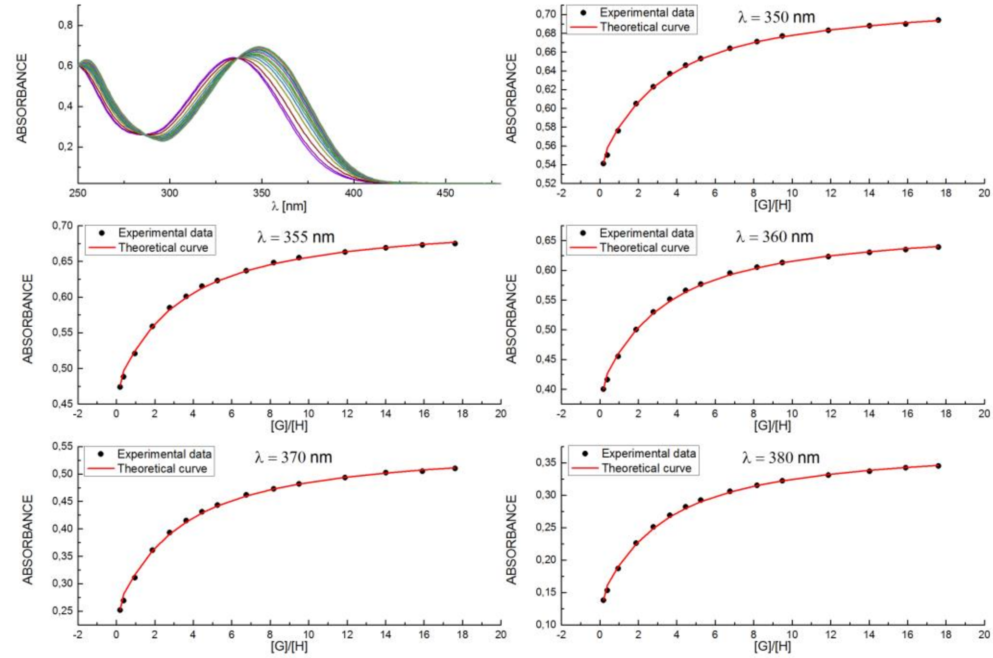

Fig. S7: 1a-3a: UV-Vis titration of receptor **1-3** with TBAAc. 1b-3b: UV-Vis titration of receptor **1-3** with TBACl in the presence of 1 equivalent of $NaClO_4$

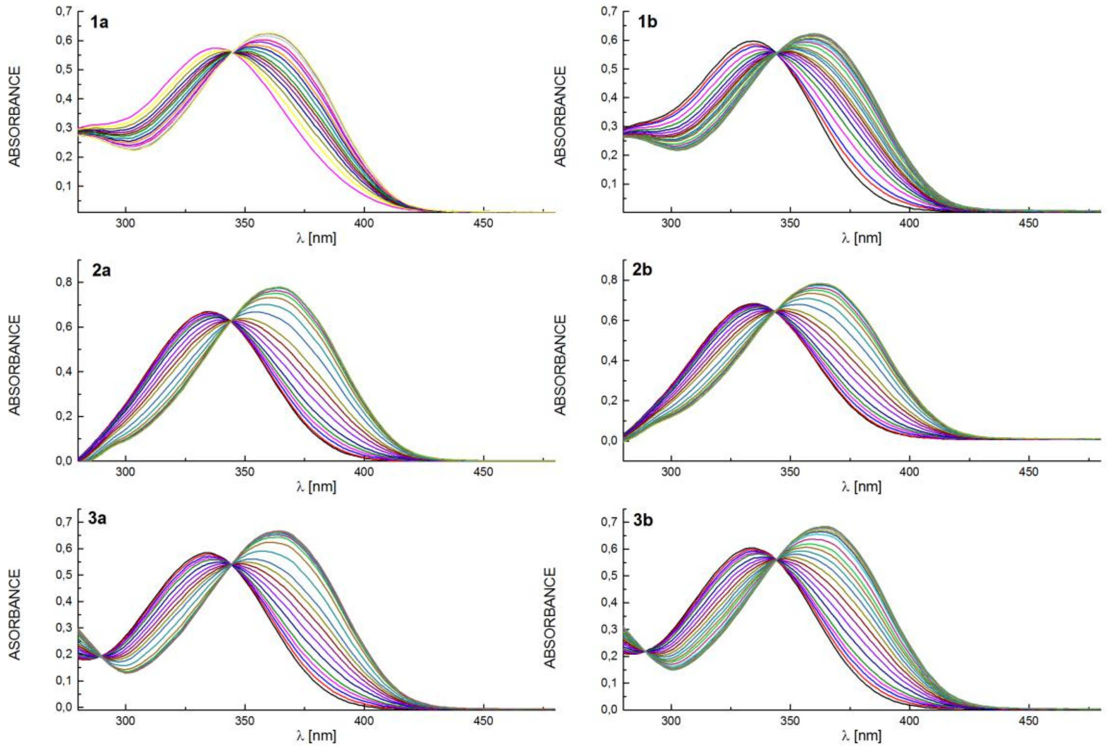

NMR TITRATION

The ^{1}H NMR titration was performed on a Bruker 300 spectrometer, at 298K in CD_3CN. In each case, a 500 µL of freshly prepared 3.13 mM solution of receptor **1** was added to a 5mm NMR tube. In the case of ion pair titration receptor was firstly pretreated with one equivalent of $NaClO_4$ (refers to receptor). Then small aliquots of solution of TBAX, containing **1** at constant concentration, were added and a spectrum was acquired after each addition. The resulting titration data were analyzed using BindFit (v0.5) package, available online at http://supramolecular.org

Fig. S8: ^{1}HNMR spectra recorded upon titration of receptor 1 in CD_3CN with TBABr.

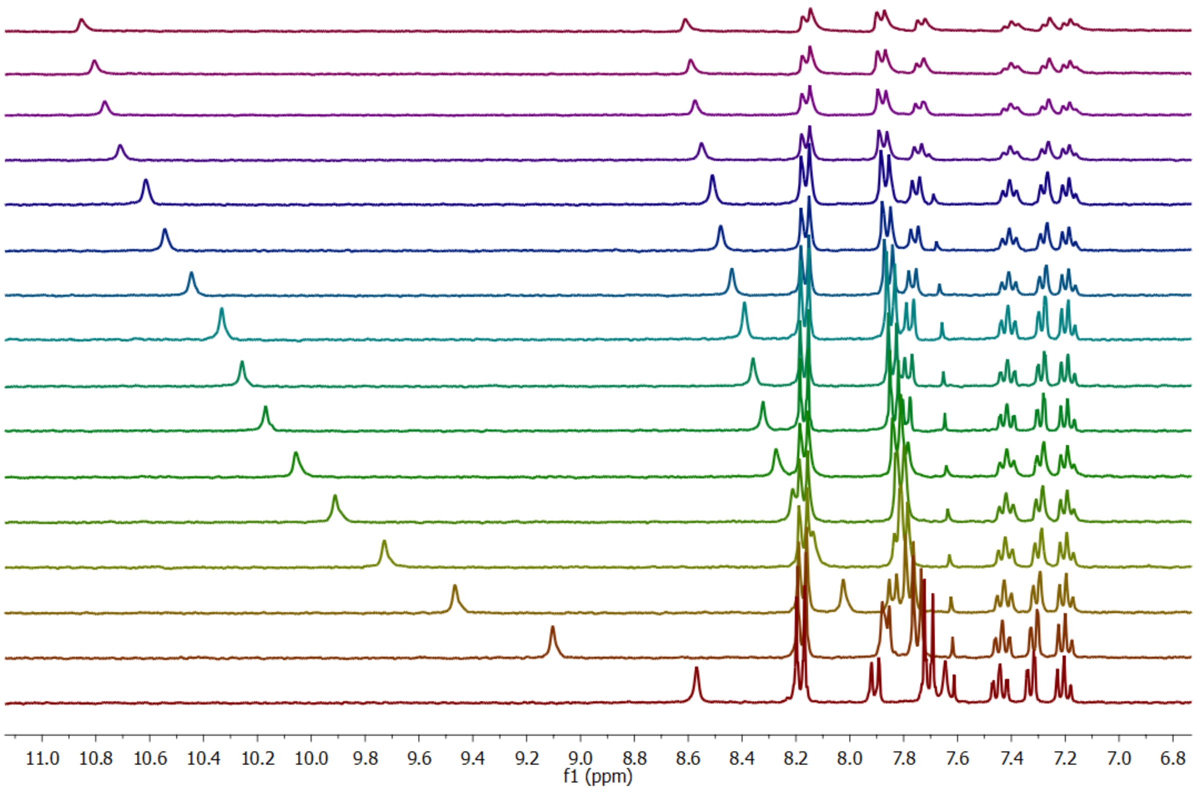

Fig. S9: ^{1}HNMR titration binding isotherms of receptor 1 in CD_3CN upon addition of increasing amounts of TBABr.

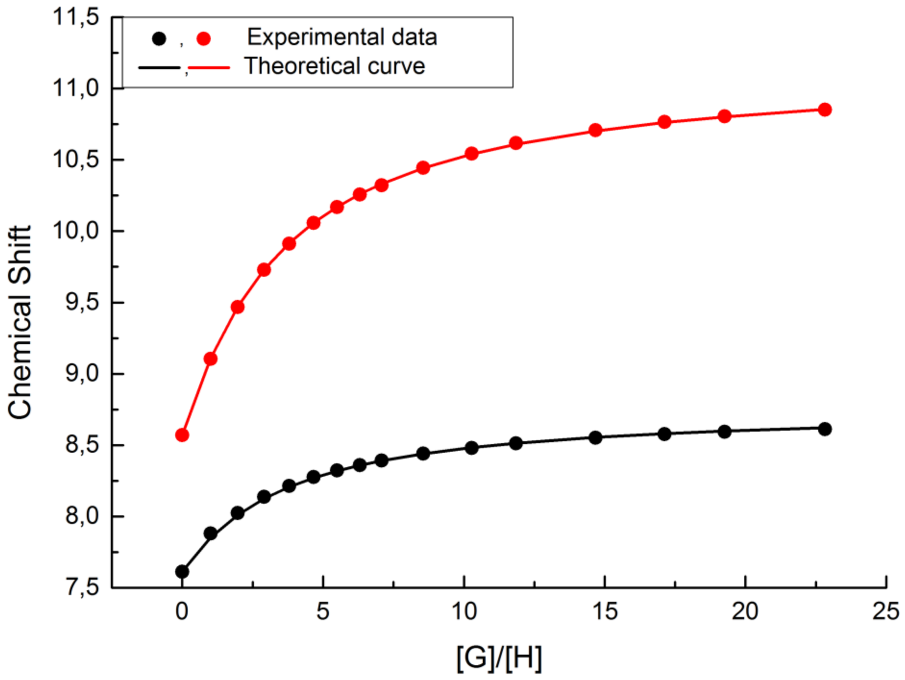

Fig. S10: ^{1}HNMR spectra recorded upon titration of receptor 1 in CD_3CN with TBABr in the presence of 1 eq. $NaClO_4$.

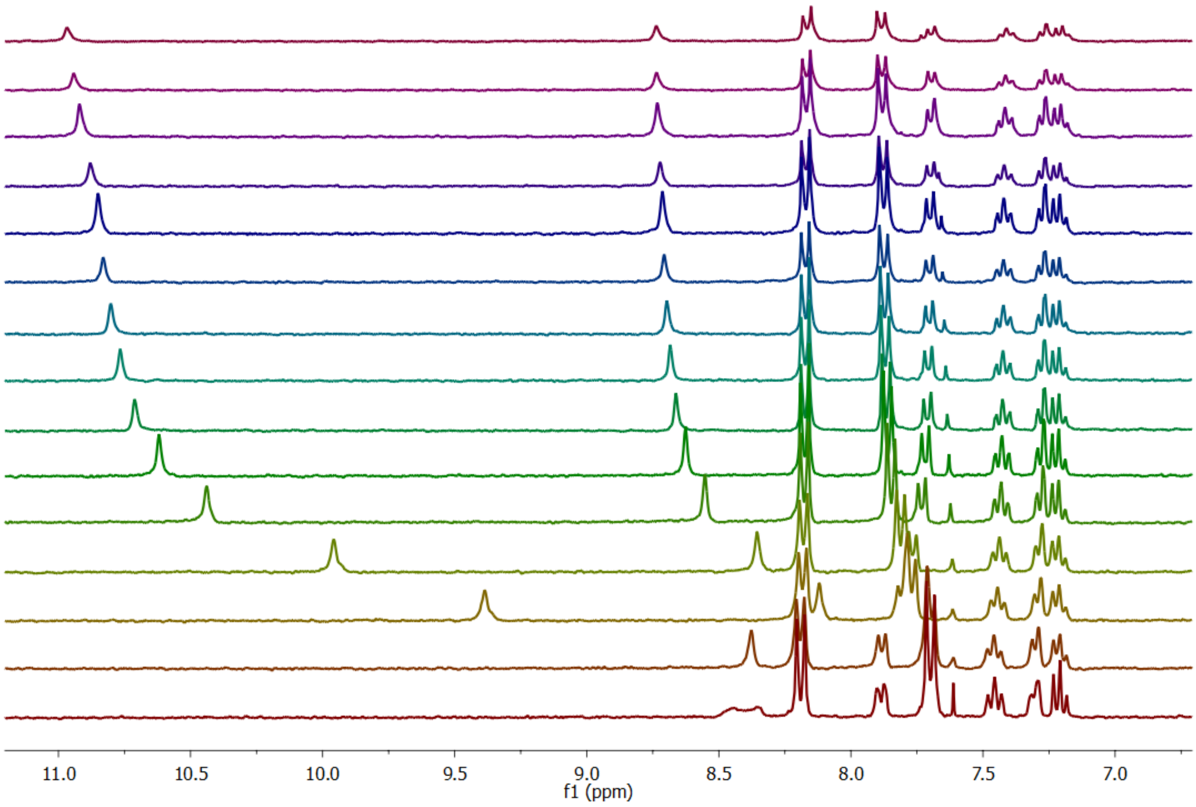

Fig. S11: ^{1}HNMR titration binding isotherms of receptor 1 in CD_3CN upon addition of increasing amounts of TBABr in the presence of 1 eq. $NaClO_4$.

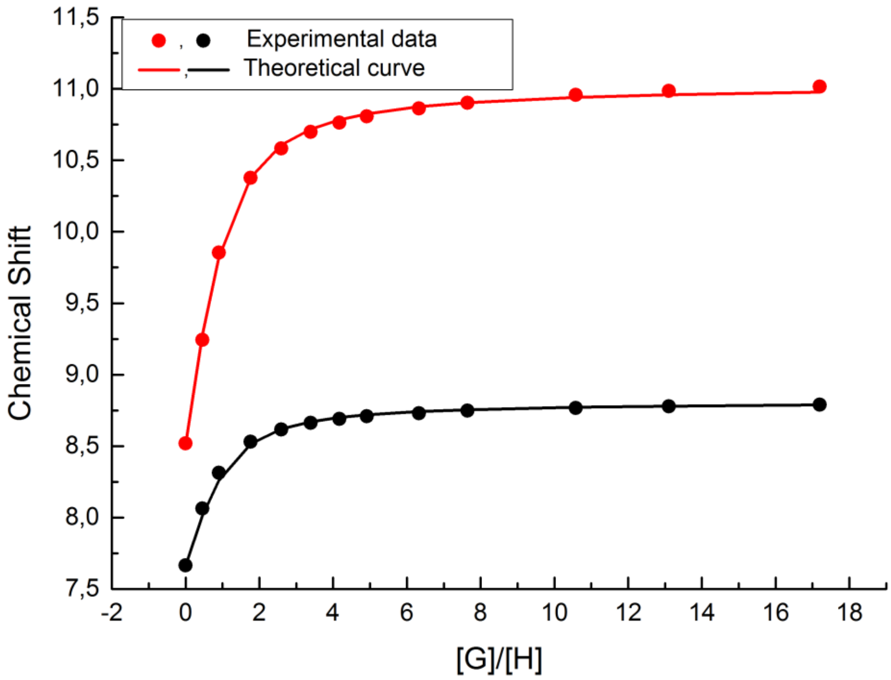

Fig. S12: ^{1}HNMR spectra recorded upon titration of receptor 2 in CD_3CN with TBABr.

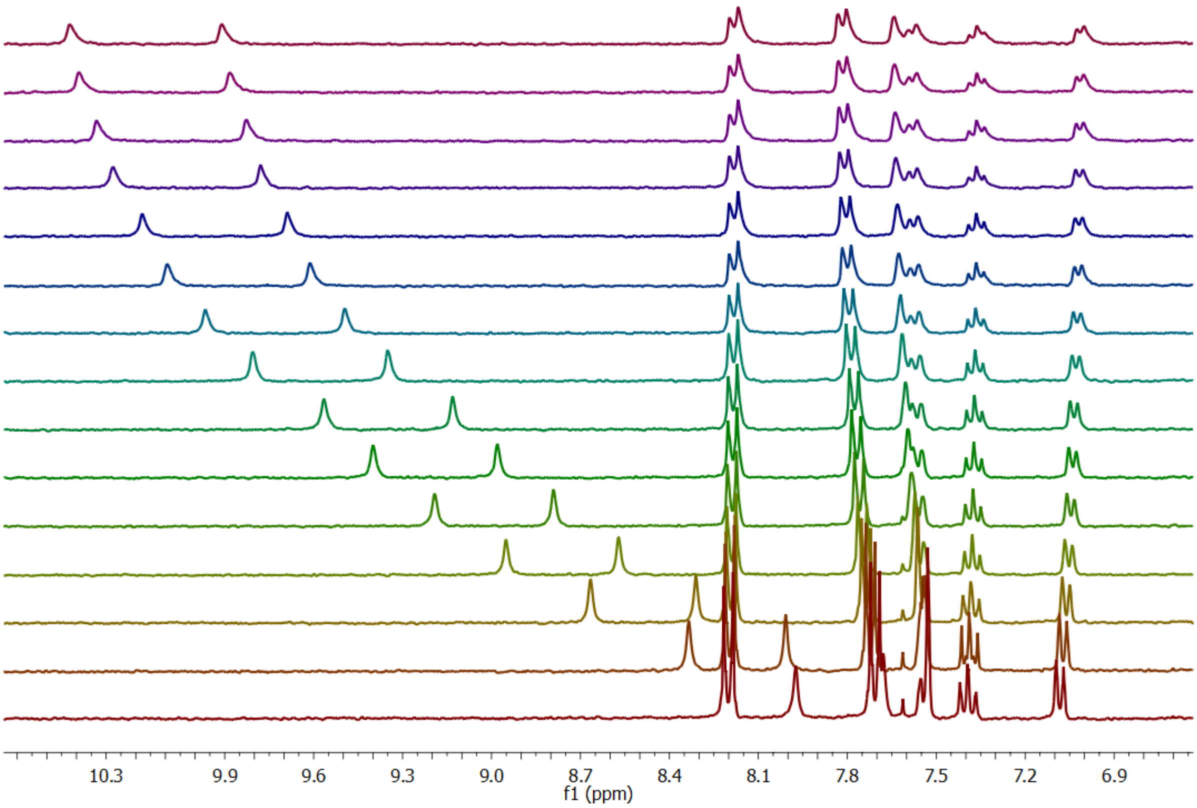

Fig. S13: ^{1}HNMR titration binding isotherms of receptor 2 in CD_3CN upon addition of increasing amounts of TBABr.

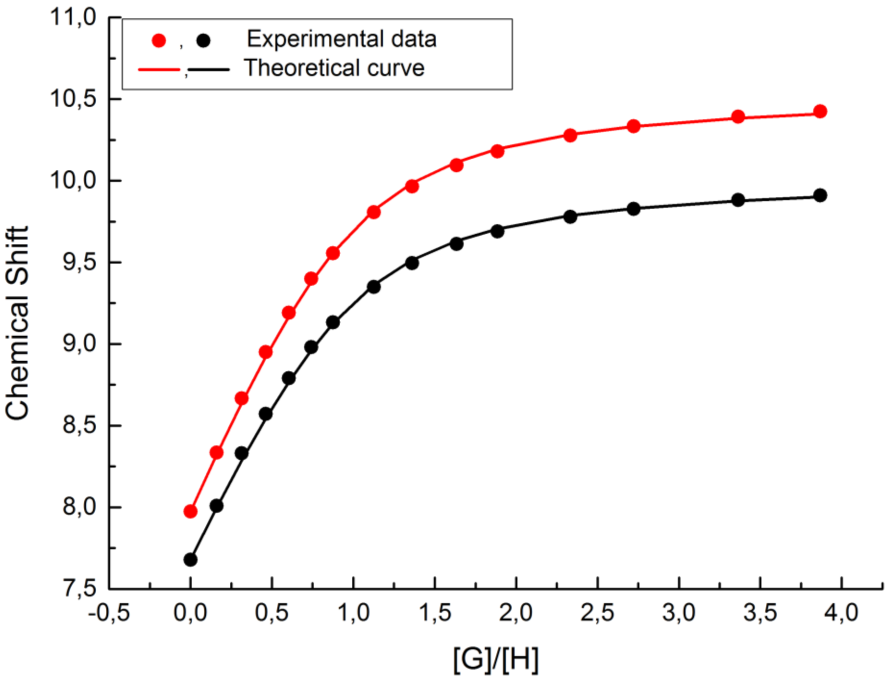

Fig. S14: ^{1}HNMR spectra recorded upon titration of receptor 2 in CD_3CN with TBABr in the presence of 1 eq. $NaClO_4$.

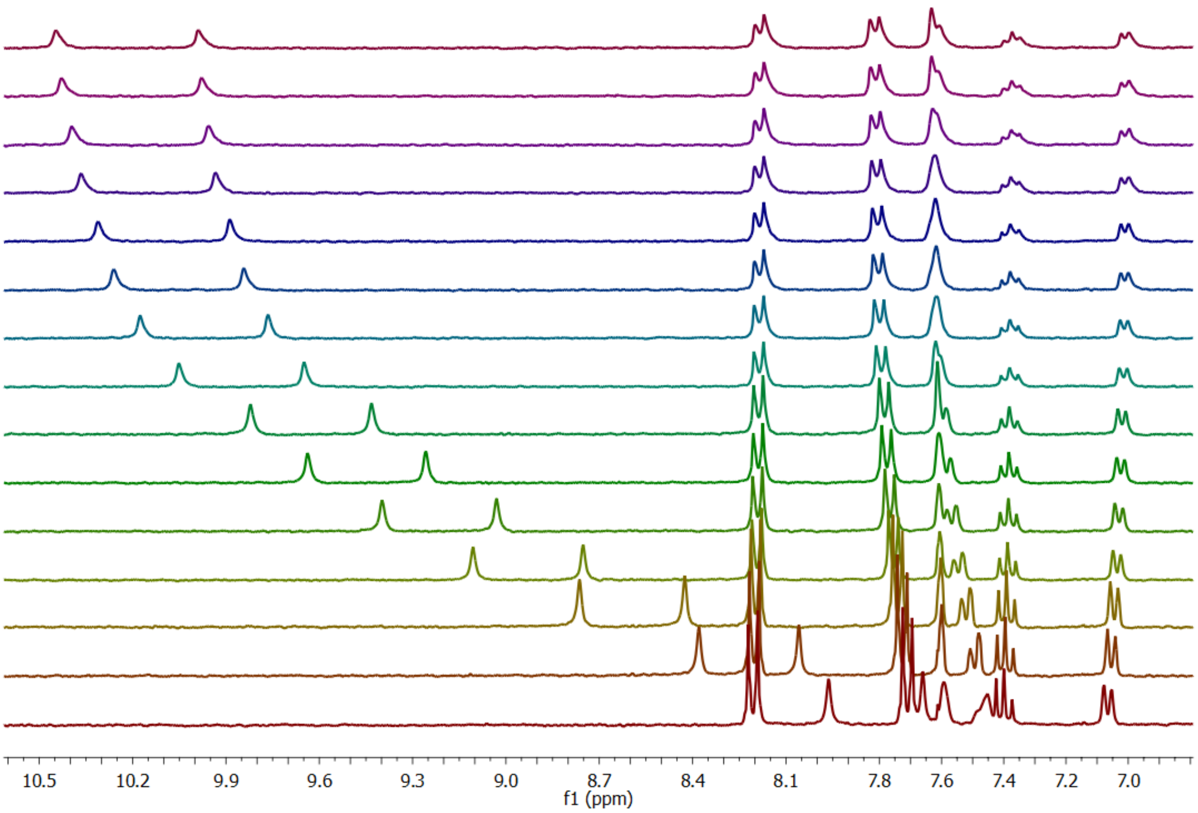

Fig. S15: ^{1}HNMR titration binding isotherms of receptor 2 in CD_3CN upon addition of increasing amounts of TBABr in the presence of 1 eq. $NaClO_4$.

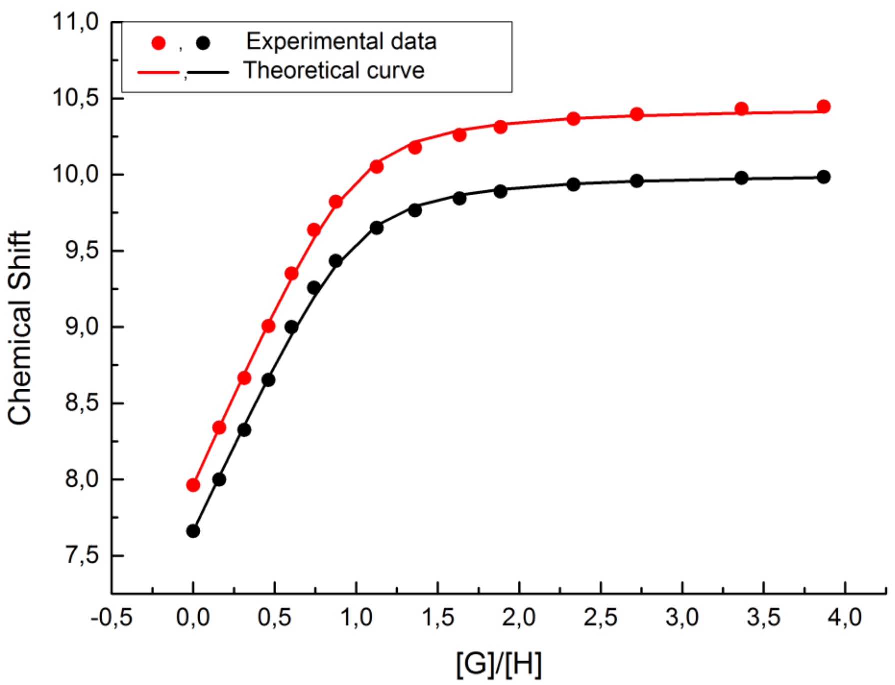

Fig. S16: ^{1}HNMR spectra recorded upon titration of receptor 3 in CD_3CN with TBABr.

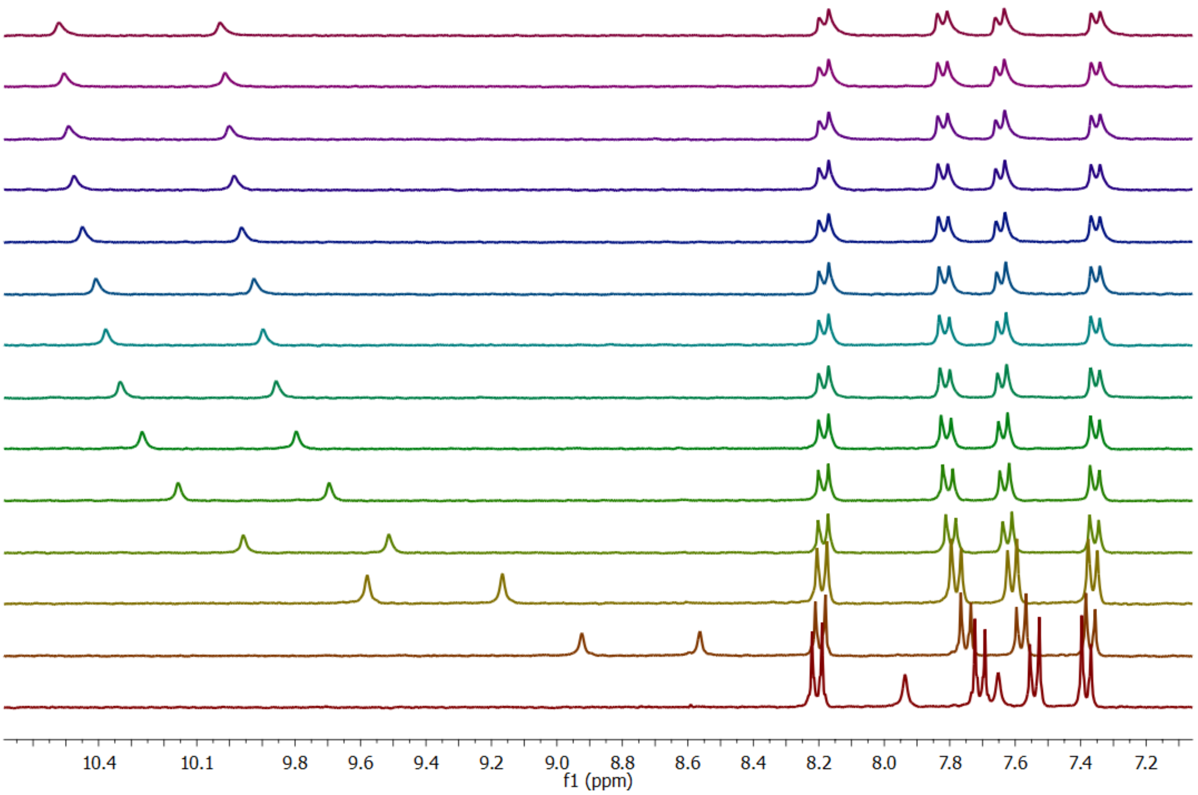

Fig. S17: ^{1}HNMR titration binding isotherms of receptor 3 in CD_3CN upon addition of increasing amounts of TBABr.

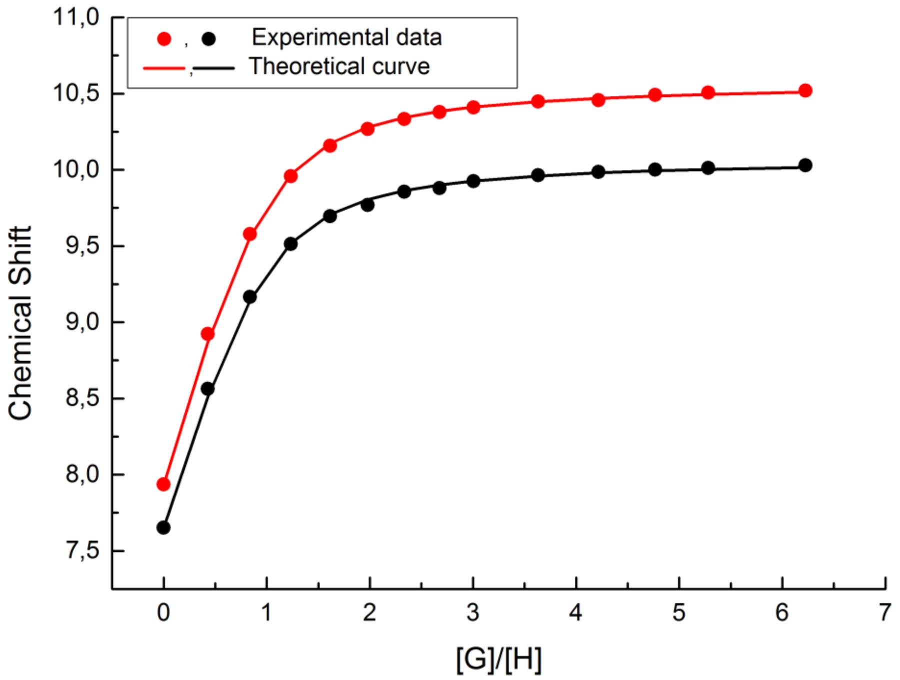

Fig. S18: ^{1}HNMR spectra recorded upon titration of receptor 3 in CD_3CN with TBABr in the presence of 1 eq. $NaClO_4$.

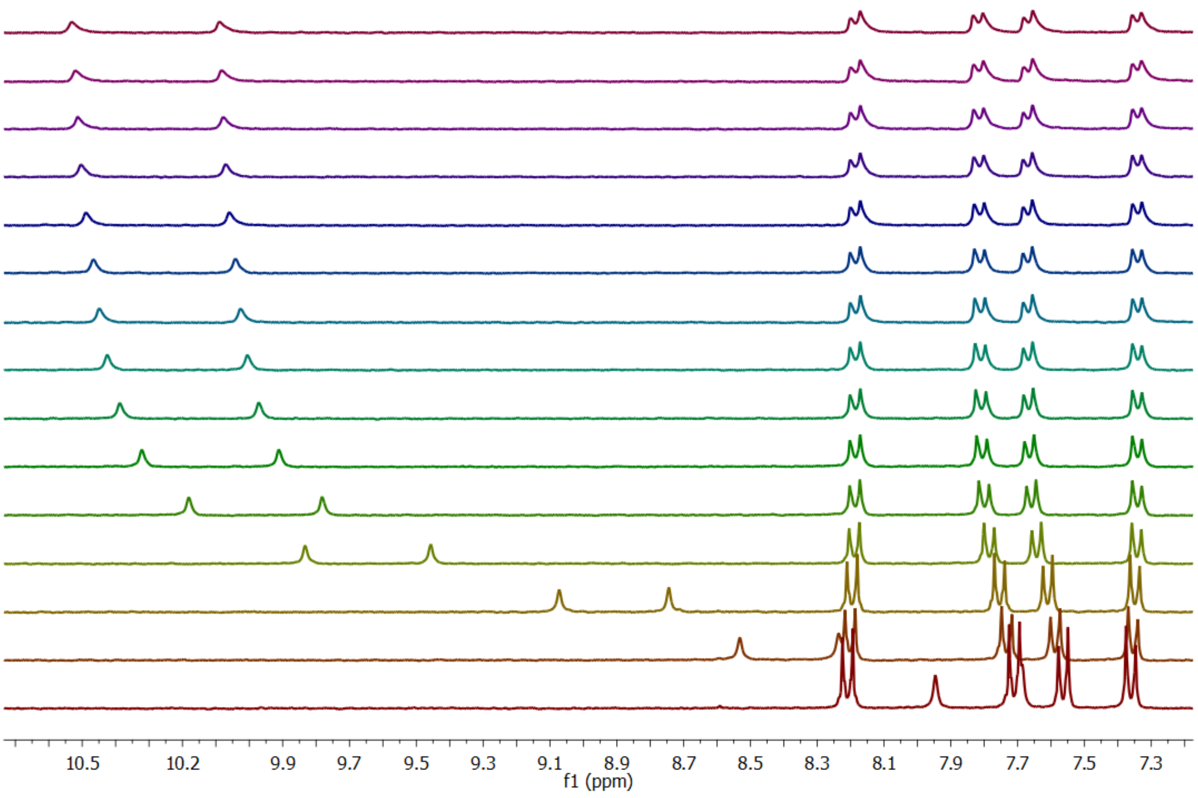

Fig. S19: ^{1}HNMR titration binding isotherms of receptor 3 in CD_3CN upon addition of increasing amounts of TBABr in the presence of 1 eq. $NaClO_4$.

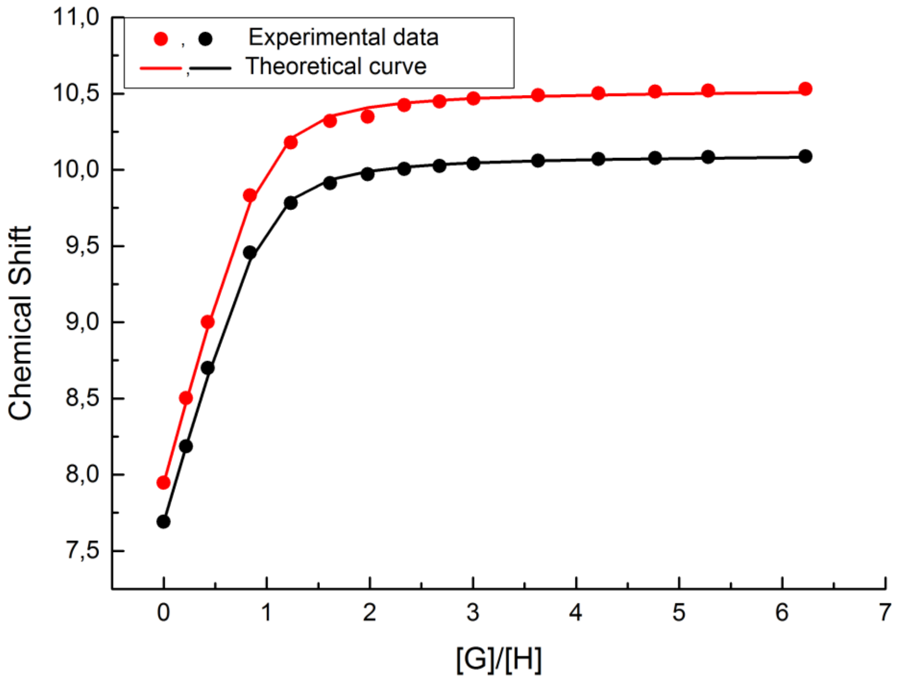

CRYSTAL DATA

The X-ray measurement of 1 was performed at 100(2) K on a Bruker D8 Venture Photon100 diffractometer equipped with a TRIUMPH monochromator and a MoKα fine focus sealed tube (λ = 0.71073 Å). A total of 2030 frames were collected with Bruker APEX2 program [1a]. The frames were integrated with the Bruker SAINT software package [1b] using a narrow-frame algorithm. The integration of the data using a triclinic unit cell yielded a total of 48072 reflections to a maximum θ angle of 25.05° (0.84 Å resolution), of which 10732 were independent (average redundancy 4.479, completeness = 99.9%, R_{int} = 2.23%, R_{sig} = 2.13%) and 8815 (82.14%) were greater than 2σ(F^2). The final cell constants of a = 13.9190(9) Å, b = 15.5110(10) Å, c = 16.3591(10) Å, α = 71.369(2)°, β = 73.035(2)°, γ = 67.080(2)°, volume = 3025.4(3) $Å^3$, are based upon the refinement of the XYZ-centroids of 9982 reflections above 20 σ(I) with 4.408° < 2θ < 50.76°. Data were corrected for absorption effects using the multi-scan method (SADABS) [1c]. The ratio of minimum to maximum apparent transmission was 0.960. The calculated minimum and maximum transmission coefficients (based on crystal size) are 0.8920 and 0.9690.

The structure was solved and refined using SHELXTL Software Package [2] using the space group P -1, with Z = 2 for the formula unit, $C_{53.83}H_{69.83}Cl_{5.49}N_8O_{18}$. The final anisotropic full-

matrix least-squares refinement on F^2 with 949 variables converged at R1 = 5.29%, for the observed data and wR2 = 13.49% for all data. The goodness-of-fit was 1.021. The largest peak in the final difference electron density synthesis was 0.768 $e^-/Å^3$ and the largest hole was -0.803 $e^-/Å^3$ with an RMS deviation of 0.056 $e^-/Å^3$. On the basis of the final model, the calculated density was 1.440 g/cm^3 and F(000), 1372 e^-.

Crystal lattice in the asymmetric part contains two organic molecules of **1**, of which one is disordered over two alternative positions in the crown ether fragment and disordered $CHCl_3$ molecules. One solvent molecule is disordered over two positions whereas the second chloroform molecule is disordered over three sites with the total occupancy equal to 0.83.

Occupancy ratio of disordered fragments in molecule of **1** and another chloroform molecule was refined at the same level yielding 0.632(3):0.368(3). To model the correct geometry of the disordered fragment of crown ether similarity restraints were used based on the corresponding fragment in the ordered molecule of **1**.

All ordered and major-component disordered non-H atoms were refined anisotropically. Some of disordered non-H atoms with lower occupancy were also refined anisotropicaly.. Most of hydrogen atoms were placed in calculated positions and refined within the riding model. The temperature factors of these hydrogen atoms were not refined and were set to be equal to either 1.2 or 1.5 times larger than U_{eq} of the corresponding heavy atom. Positions of hydrogen atoms engaged in hydrogen bonds, were refined together with their isotropic temperature factors. The atomic scattering factors were taken from the International Tables [3]. ORTEP plot (P=50%) of **1** is presented in **Figure S20**.

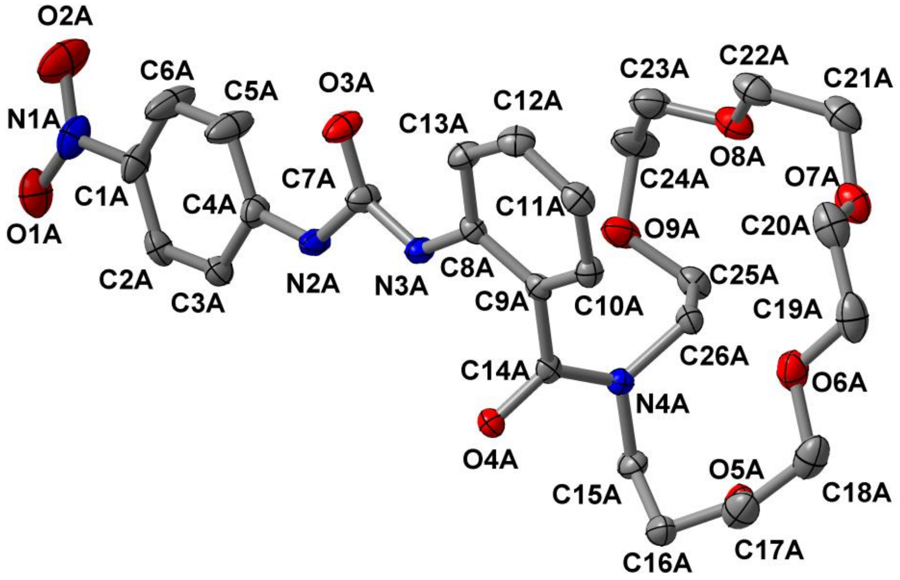

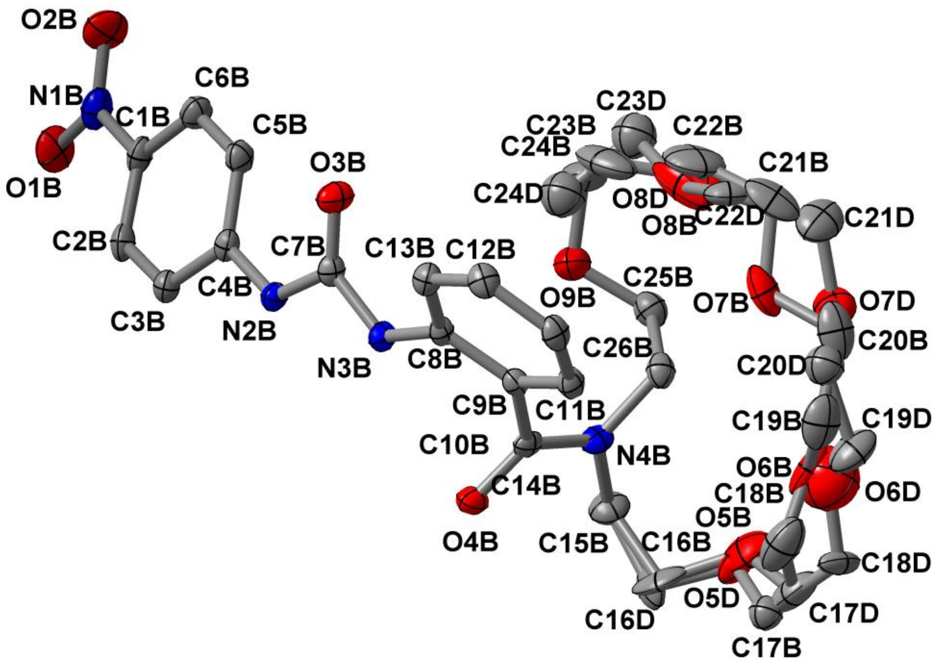

Fig. S20. ORTEP plot (P=50%) of **1**. Disordered solvent molecules and hydrogens are omitted for clarity.

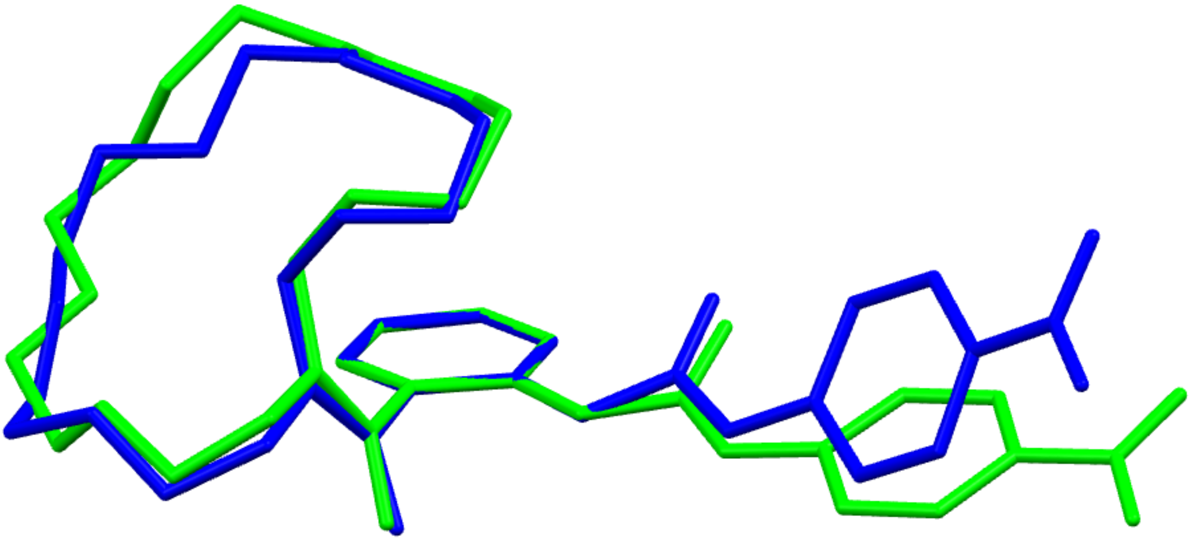

Figure S21. ORTEP plot (P=50%) of **1**. Hydrogens and disordered parts are omitted for clarity.

The X-ray measurement of **1•NaPF6** was performed at 100(2) K on a Bruker D8 Venture Photon100 diffractometer equipped with a TRIUMPH monochromator and a MoKα fine focus sealed tube (λ = 0.71073 Å). A total of 2320 frames were collected with Bruker APEX2 program [1a]. The integration of the data using a triclinic unit cell yielded a total of 39203 reflections to a maximum θ angle of 25.05° (0.84 Å resolution), of which 6246 were independent (average redundancy 6.276, completeness = 99.8%, R_{int} = 2.98%, R_{sig} = 2.06%) and 5083 (81.38%) were greater than 2σ(F^2). The final cell constants of a = 8.4135(4) Å, b = 13.0050(6) Å, c = 16.7431(8) Å, α = 104.0055(12)°, β = 92.4228(13)°, γ = 92.5966(13)°, V =

1773.02(15) Å^3, are based upon the refinement of the XYZ-centroids of 9321 reflections above 20 $\sigma(I)$ with 6.068° < 2θ < 50.75°. Data were corrected for absorption effects using the multi-scan method (SADABS) [1c]. The ratio of minimum to maximum apparent transmission was 0.925. The calculated minimum and maximum transmission coefficients (based on crystal size) are 0.951 and 0.984.

The structure was solved and refined using SHELXTL Software Package [X2] sing the space group *P*-1, with *Z* = 1 for the formula unit, $C_{59}H_{82.50}F_{12}N_{9.50}Na_2O_{19}P_2$. The final anisotropic full-matrix least-squares refinement on F^2 with 610 variables converged at *R*1 = 5.09%, for the observed data and *wR*2 = 13.28% for all data. The goodness-of-fit was 1.046. The largest peak in the final difference electron density synthesis was 0.558 e^-/Å^3 and the largest hole was -0.436 e^-/Å^3 with an RMS deviation of 0.062 e^-/Å^3. On the basis of the final model, the calculated density was 1.465 g/cm^3 and *F*(000), 815 e^-.

The ligand molecule is disordered in the crown ether fragment and the *p*-nitrophenyl part. In both cases the atoms are disordered over two sites with refined occupancy ratio equal to 0.789(7):0.211(7) and 0.901(12):0.099(12) in the crown ether and *p*-nitrophenyl moieties respectively. The crystal contains the ligand molecule complexing Na^+ ion by the crown ether unit altogether located in general position. The PF_6^- moiety, located on the center of inversion, is bound by Na^+…F contacts between two ligands. The electroneutrality of the crystal is provided by the presence of additional PF_6^- ion (located in general position) which is disordered over two sites with not refined occupancy ratio equal to 0.3:0.2, giving overall occupancy of the moiety equal to 0.5. The PF_6^- anion share the space with diethyl ether with atoms occupancy equal to 0.5. In addition the structure contains acetonitile molecule disordered over two sites with not refined occupancy ratio equal to 0.5:0.25.

The non-hydrogen atoms, including major component disordered moieties, were refined anisotropically. Most of hydrogen atoms were placed in calculated positions and refined within the riding model. Position and temperature factors of two hydrogen atoms engaged in hydrogen bonds were refined. The temperature factors of all other hydrogen atoms were not refined and were set to be either 1.2 or 1.5 times larger than *Ueq* of the corresponding heavy atom. The atomic scattering factors were taken from the International Tables [3]. ORTEP plot (P=50%) of **1•NaPF$_6$** is presented in **Figure S20**.

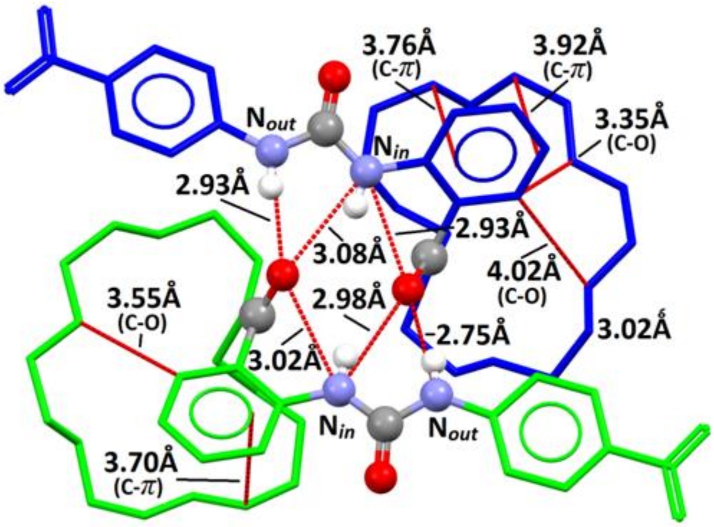

Fig. S22. Selected hydrogen bonds and CH-Π interactions in the dimer of receptor 1 in the crystal structure.

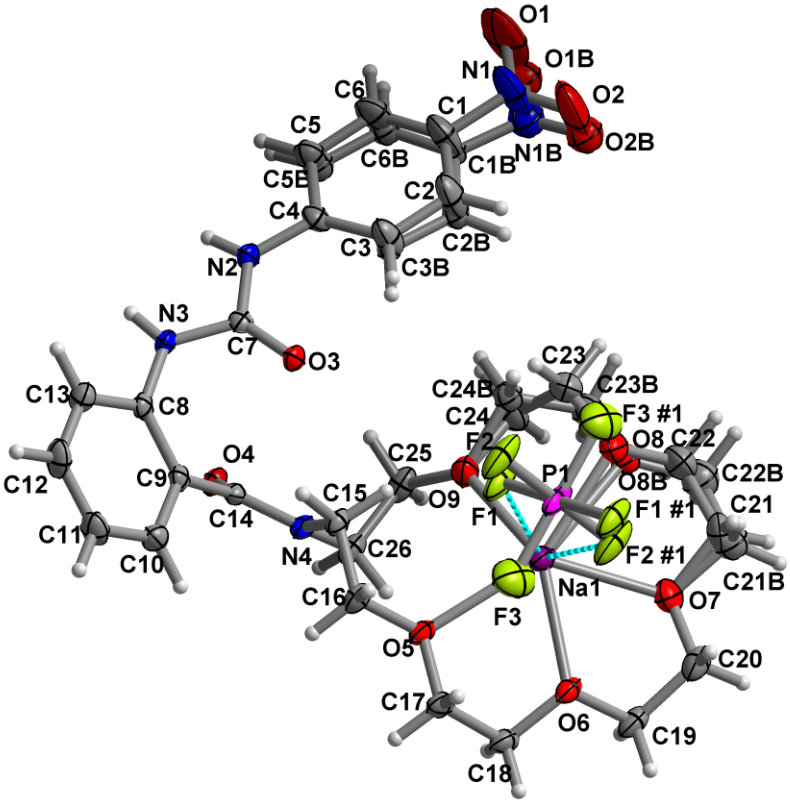

Fig. S23. ORTEP plot (P=50%) of **1•NaPF₆**.

The X-ray measurement of **2•NaBr** was performed at 100(2) K on a Bruker D8 Venture Photon100 diffractometer equipped with a TRIUMPH monochromator and a MoKα fine focus sealed tube (λ = 0.71073 Å). A total of 2320 frames were collected with Bruker APEX2

program [1a]. The frames were integrated with the Bruker SAINT software package [1b] using a narrow-frame algorithm. The integration of the data using a triclinic unit cell yielded a total of 47583 reflections to a maximum θ angle of 25.05° (0.84 Å resolution), of which 7246 were independent (average redundancy 6.567, completeness = 99.9%, R_{int} = 2.49%, R_{sig} = 1.72%) and 6531 (90.13%) were greater than $2\sigma(F^2)$. The final cell constants of a = 12.7282(10) Å, b = 13.6326(11) Å, c = 14.5482(12) Å, α = 106.806(2)°, β = 113.890(2)°, γ = 102.082(2)°, volume = 2050.1(3) Å^3, are based upon the refinement of the XYZ-centroids of 1070 reflections above 20 $\sigma(I)$ with 3.578° < 2θ < 58.35°. Data were corrected for absorption effects using the multi-scan method (SADABS) [1c]. The ratio of minimum to maximum apparent transmission was 0.926. The calculated minimum and maximum transmission coefficients (based on crystal size) are 0.700 and 0.833.

The structure was solved and refined using SHELXTL Software Package [2] using the space group P -1, with Z = 2 for the formula unit, $C_{33.36}H_{54.73}BrN_4NaO_{12}S_{2.32}$. The final anisotropic full-matrix least-squares refinement on F^2 with 543 variables converged at $R1$ = 2.86%, for the observed data and $wR2$ = 6.88% for all data. The goodness-of-fit was 1.048. The largest peak in the final difference electron density synthesis was 0.736 e^-/Å^3 and the largest hole was -0.374 e^-/Å^3 with an RMS deviation of 0.051 e^-/Å^3. On the basis of the final model, the calculated density was 1.427 g/cm^3 and F(000), 924 e^-.

The structure contains disordered DMSO and diethyl ether solvent molecules. These molecules are sharing the same site with refined occupancy ratio equal to 0.683(3) and 0.317(3) for DMSO and Et_2O molecules respectively. DMSO moiety is, in addition, disordered over two sites with not refined occupancy ratio equal to 0.5:0.5. The O and C atoms are common for both sites. In addition one of DMSO molecules coordinating Na ion is disordered over two sites with S and O atoms common. The refined occupancy ration of both sites is equal to 0.54(1):0.46(1). To preserve reasonable geometry of disordered DMSO molecules geometry constraints were used. Almost all non-H atoms were refined anisotropically. The exception was made for all heavy atoms of disordered DMSO solvent molecule. Most of hydrogen atoms were placed in calculated positions and refined within the riding model. The temperature factors of these hydrogen atoms were not refined and were set to be equal to either 1.2 or 1.5 times larger than U_{eq} of the corresponding heavy atom. Positions of two hydrogen atoms in the urea fragment engaged in hydrogen bonds were refined together with their isotropic temperature factors. The atomic scattering factors were taken from the International Tables [3]. ORTEP plot (P=50%) of **2•NaBr** is presented in **Figure S24**.

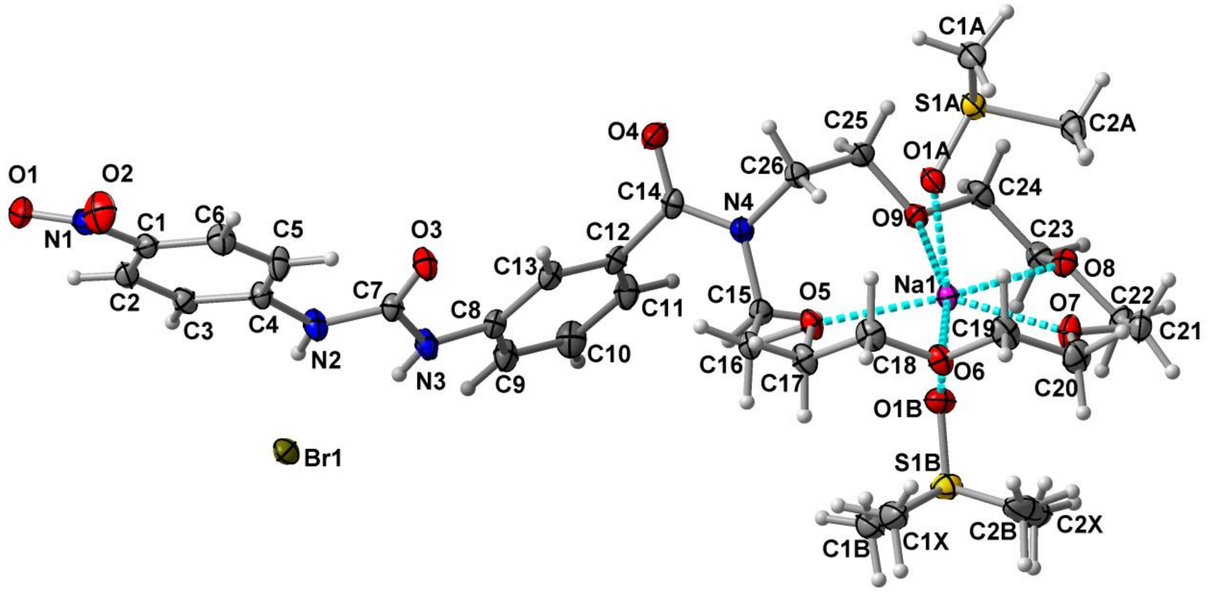

Fig. S24. ORTEP plot (P=50%) of **2•NaBr**.

The X-ray measurement of **3•NaBr** was performed at 100(2) K on a Bruker D8 Venture Photon100 diffractometer equipped with a TRIUMPH monochromator and a MoKα fine focus sealed tube (λ = 0.71073 Å). A total of 2310 frames were collected with Bruker APEX2 program [1a]. The frames were integrated with the Bruker SAINT software package [1b] using a narrow-frame algorithm. The integration of the data using a monoclinic unit cell yielded a total of 212011 reflections to a maximum θ angle of 25.05° (0.84 Å resolution), of which 22597 were independent (average redundancy 9.382, completeness = 99.8%, R_{int} = 7.87%, R_{sig} = 4.05%) and 17759 (78.59%) were greater than 2σ(F^2). The final cell constants of a = 15.488(2) Å, b = 14.836(2) Å, c = 55.956(9) Å, β = 96.5751(19)°, V = 12773.(3) Å^3, are based upon the refinement of the XYZ-centroids of 9746 reflections above 20 $\sigma(I)$ with 5.863° < 2θ < 50.77°. Data were corrected for absorption effects using the multi-scan method (SADABS) [1c]. The ratio of minimum to maximum apparent transmission was 0.831. The calculated minimum and maximum transmission coefficients (based on crystal size) are 0.630 and 0.883.

The structure was solved and refined using SHELXTL Software Package [2] using the space group $P2_1/n$, with Z = 16 for the formula unit, $C_{27}H_{38}BrN_4NaO_{10}$. The final anisotropic full-matrix least-squares refinement on F^2 with 1593 variables converged at $R1$ = 6.80%, for the observed data and $wR2$ = 14.08% for all data. The goodness-of-fit was 1.346. The largest peak in the final difference electron density synthesis was 0.997 e^-/Å^3 and the largest hole was -1.538 e^-/Å^3 with an RMS deviation of 0.086 e^-/Å^3. On the basis of the final model, the calculated density was 1.418 g/cm^3 and F(000), 5664 e^-.

The non-hydrogen atoms were refined anisotropically. Most of hydrogen atoms were placed in calculated positions and refined within the riding model. Position of hydrogen atoms engaged in hyfrogen bonds were refined. The temperature factors of all but four hydrogen atoms were not refined and were set to be either 1.2 or 1.5 times larger than *Ueq* of the corresponding heavy atom, temperature factors of four hydrogen atoms in the hydroxyl groups of MeOH were refined. The atomic scattering factors were taken from the International Tables [3]. ORTEP plot (P=50%) of 2•NaBr is presented in **Figure S25**.

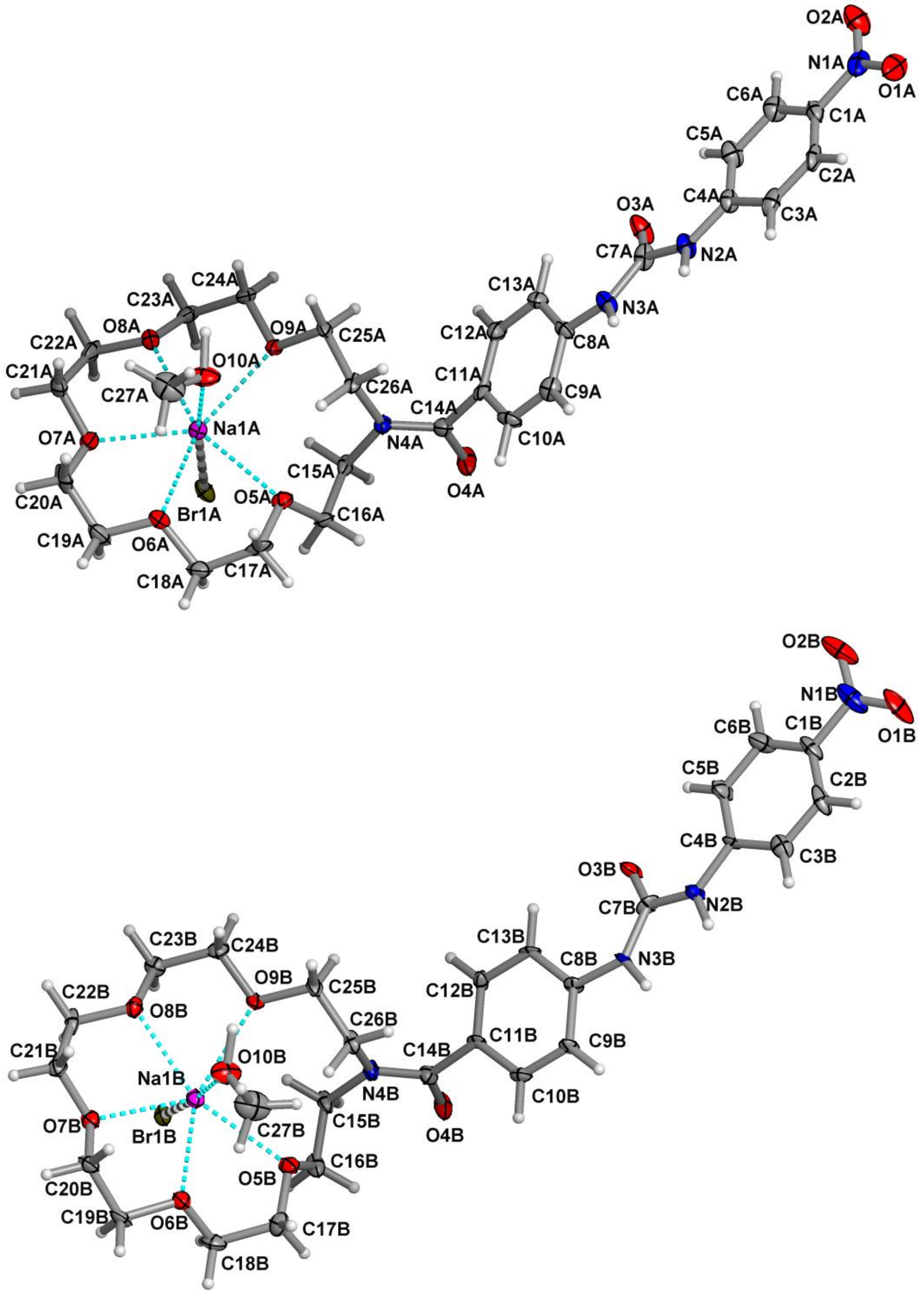

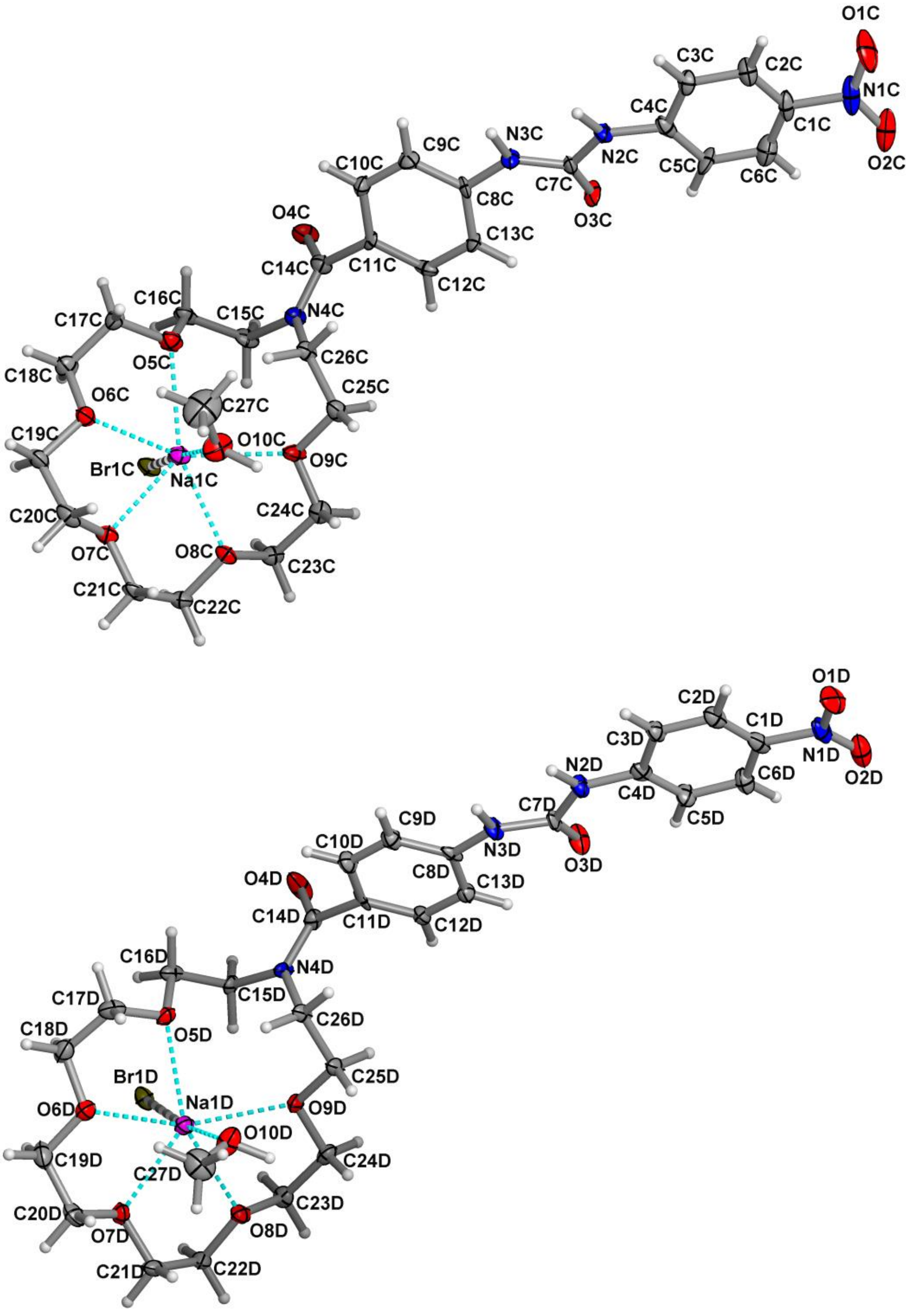

Fig. S25. ORTEP plot (P=50%) of **3•NaBr**.

2

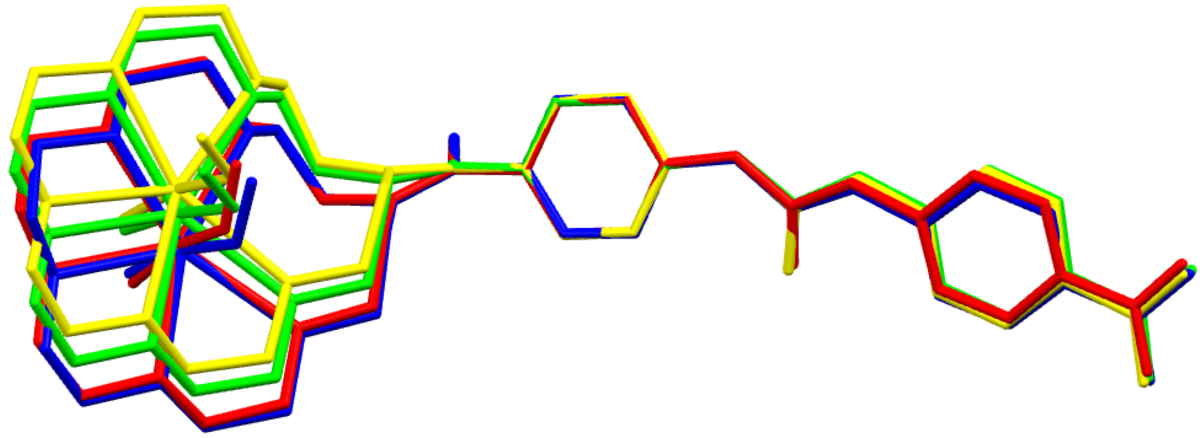

Fig. S26. Superimposition (by central ring C atoms) of **3•NaBr**.

NMR Spectra

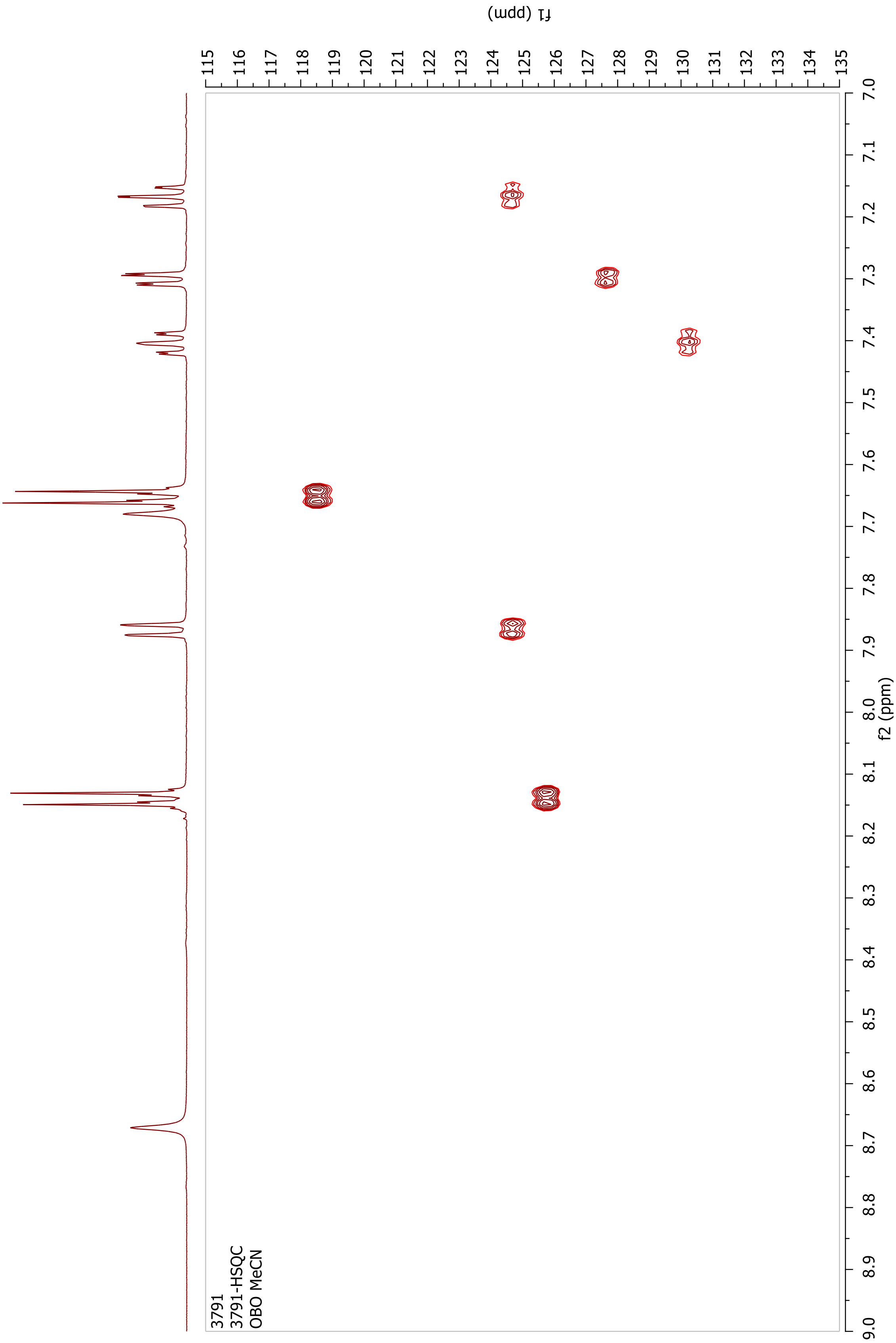

Figure S27. Part of HSQC spectrum (aromatic and urea region) of receptor **1** in CD_3CN.

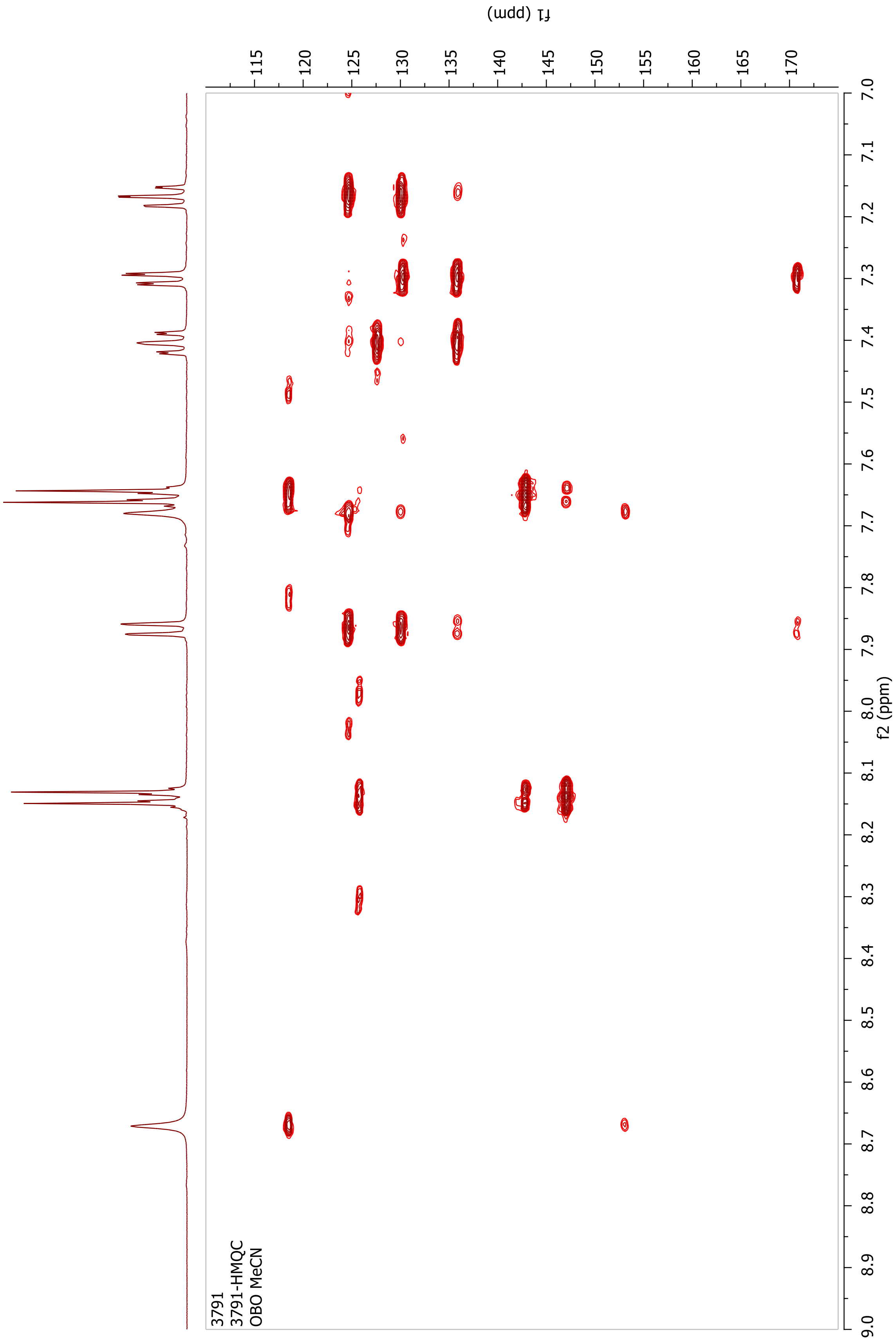

Figure S28. Part of HMBC spectrum (aromatic and urea region) of receptor **1** in CD_3CN.

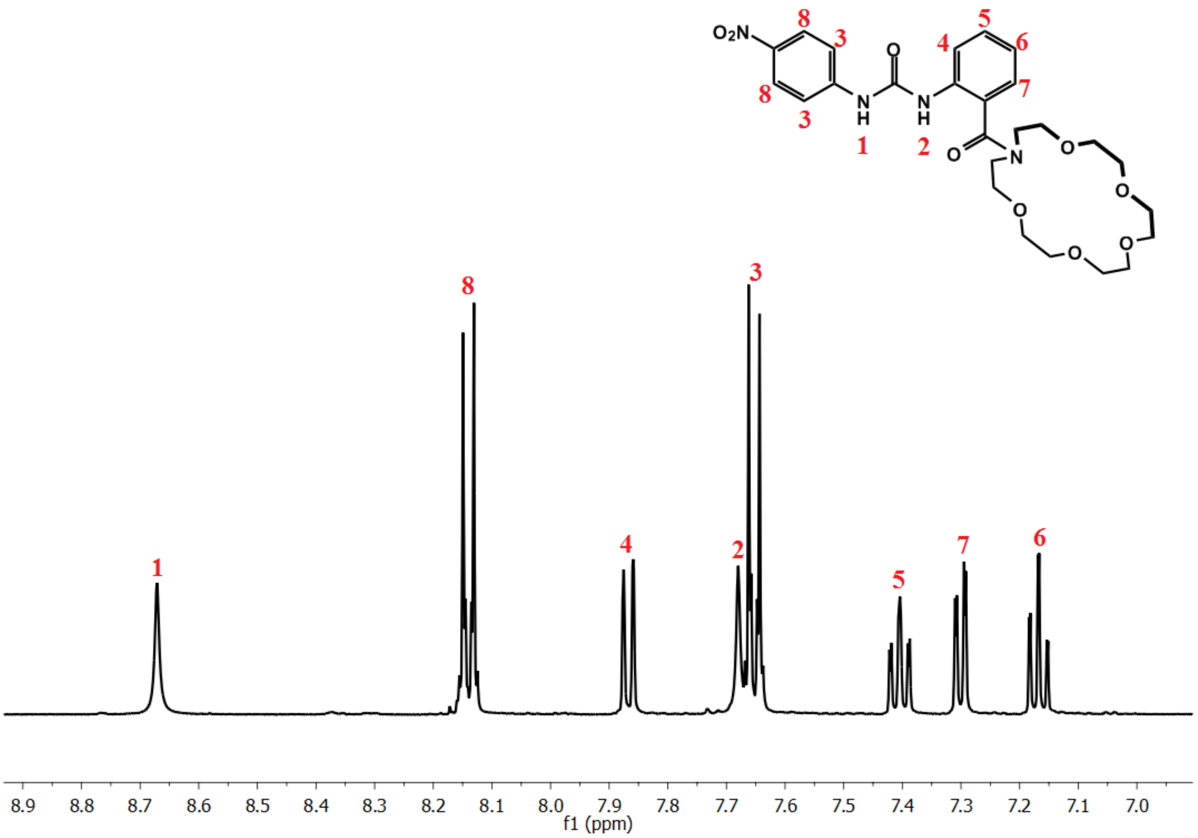

Figure S29. Assignment of signals in ^{1}H NMR spectrum (aromatic and urea region) of receptor **1** in CD_3CN.

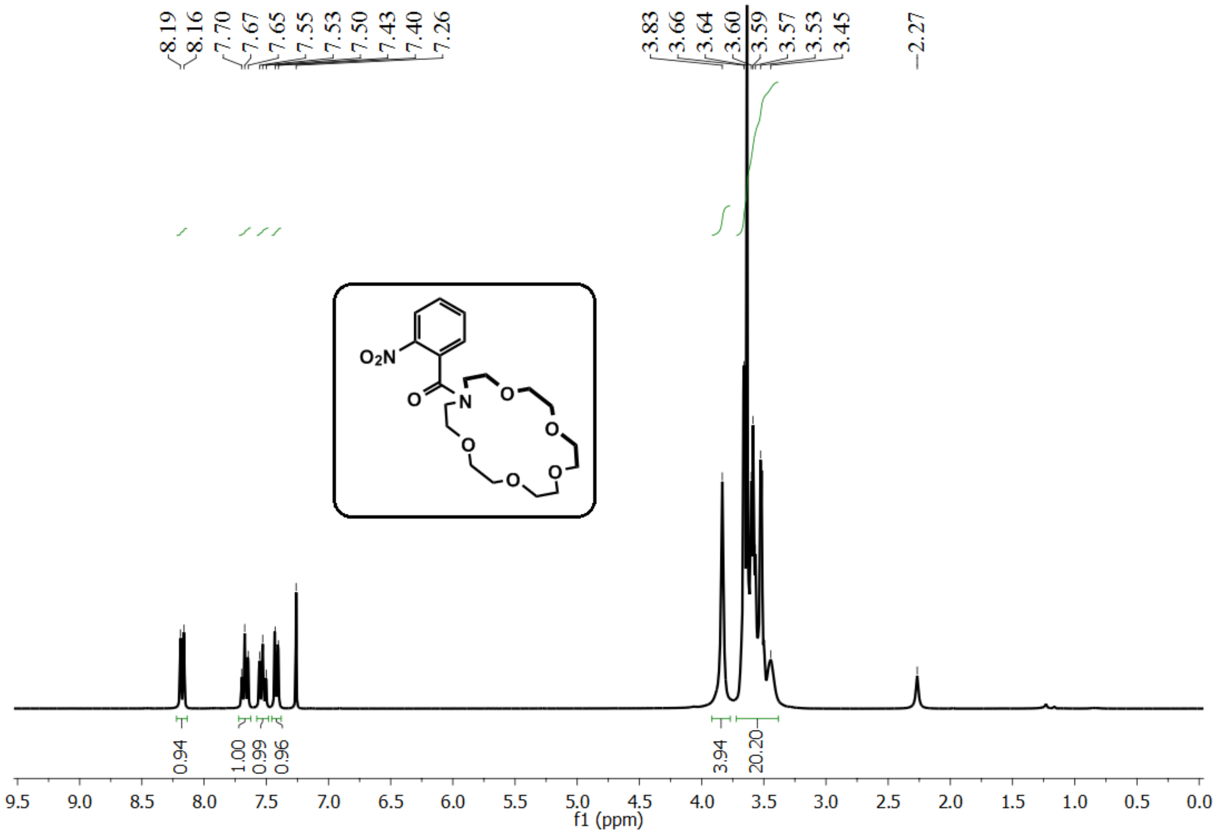

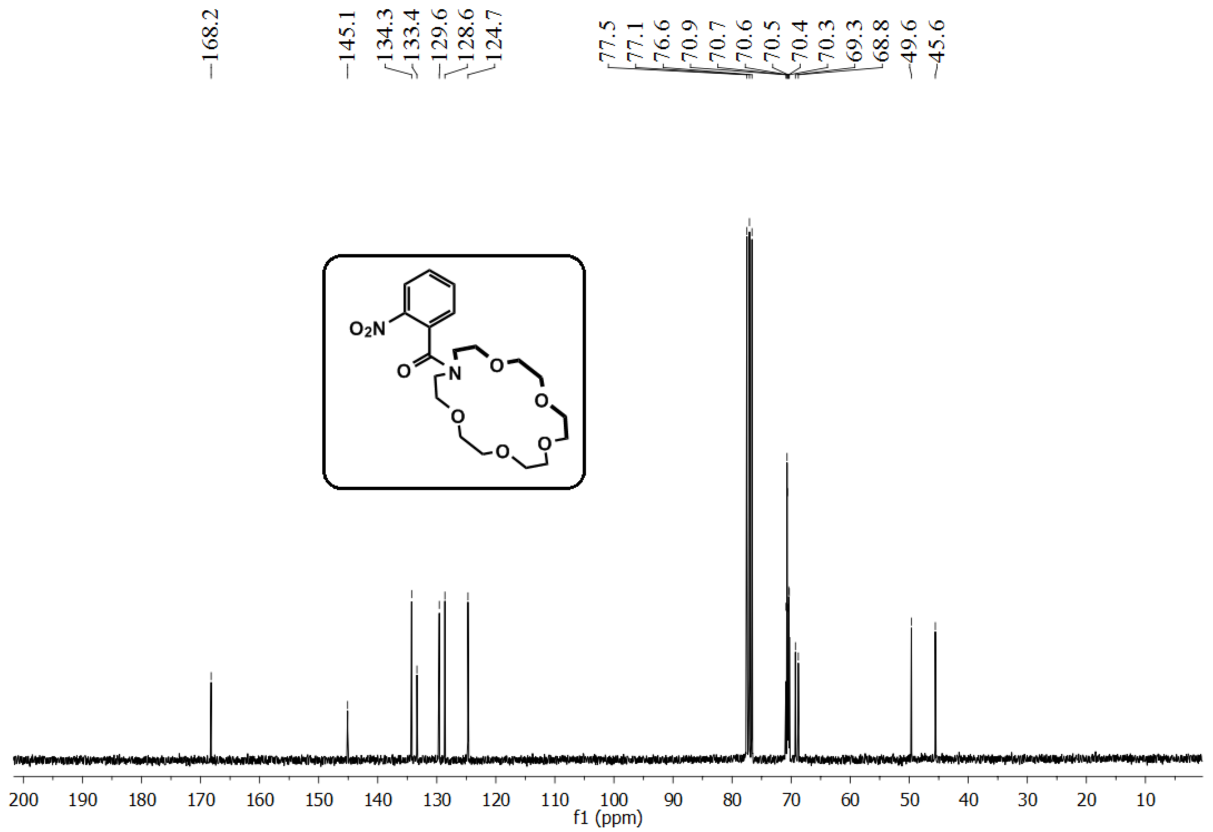

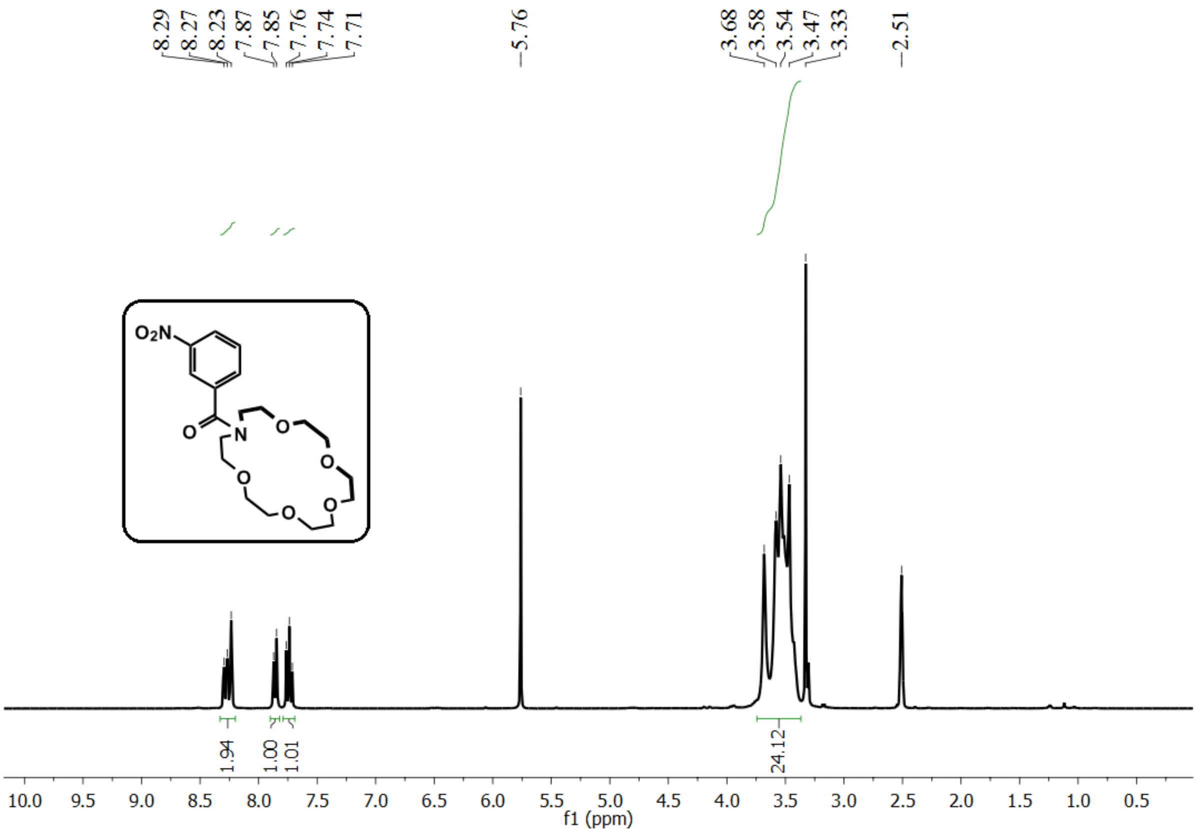
8.29
8.27
8.23
7.87
7.85
7.76
7.74
7.71
5.76
3.68
3.58
3.54
3.47
3.33
2.51
O_2N
O
N
O
O
O
O
1.94
1.00
1.01
24.12
f1 (ppm)

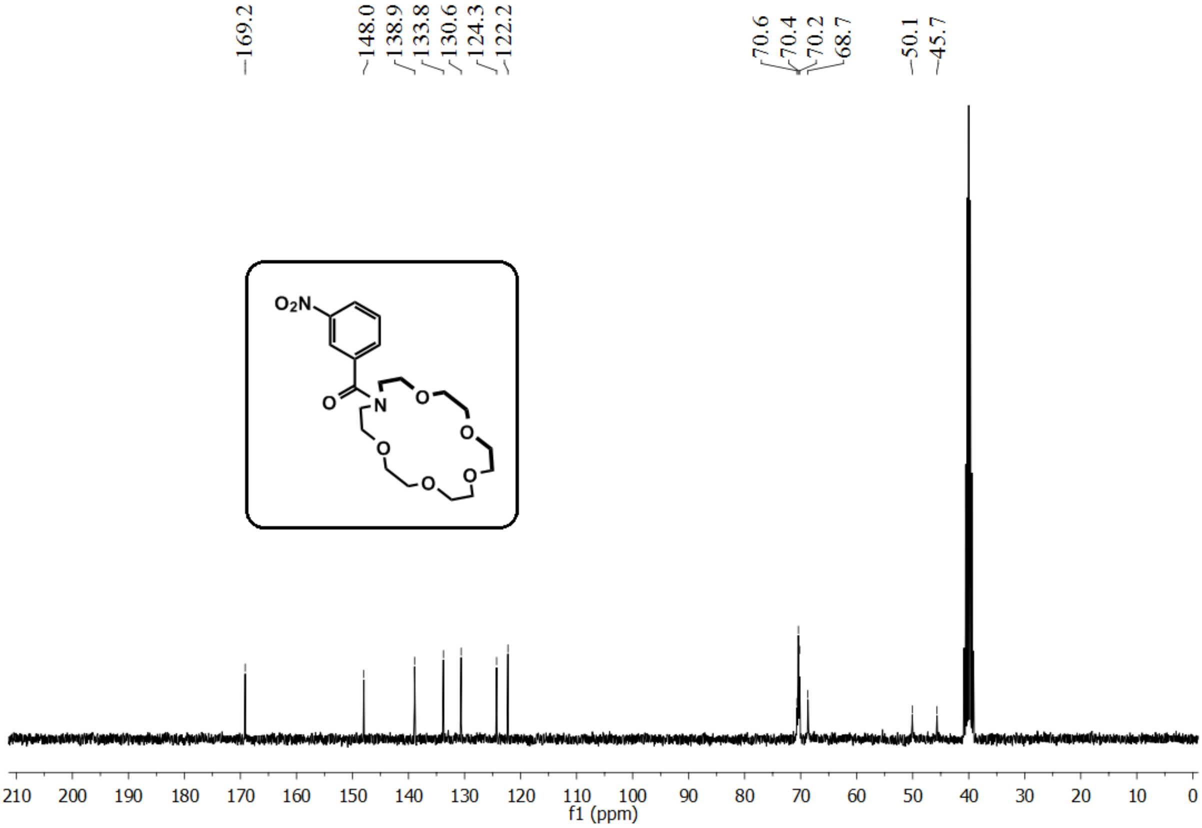
169.2
148.0
138.9
133.8
130.6
124.3
122.2
70.6
70.4
70.2
68.7
50.1
45.7
O_2N
O
N
O
O
O
O
f1 (ppm)

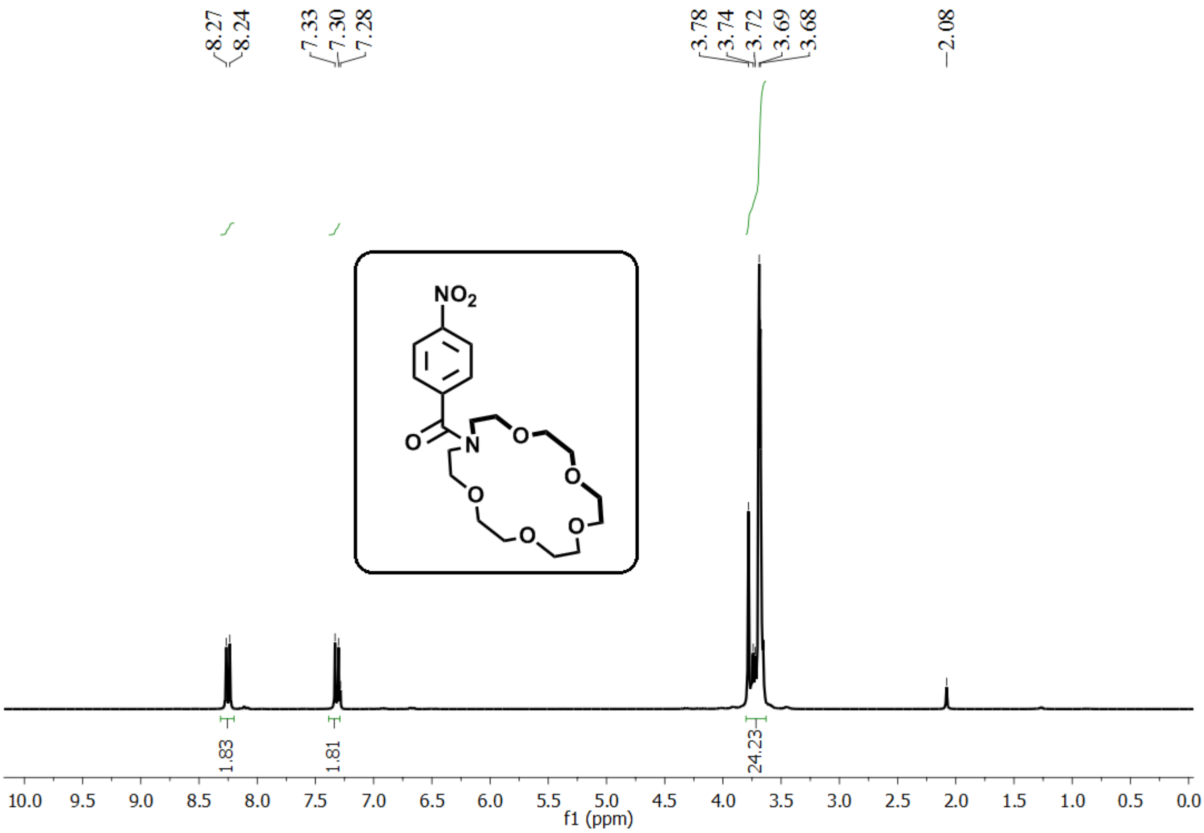
8.27
8.24
7.33
7.30
7.28
3.78
3.74
3.72
3.69
3.68
2.08
NO2
O
N
O
O
O
O
1.83
1.81
24.23
10.0 9.5 9.0 8.5 8.0 7.5 7.0 6.5 6.0 5.5 5.0 4.5 4.0 3.5 3.0 2.5 2.0 1.5 1.0 0.5 0.0
f1 (ppm)

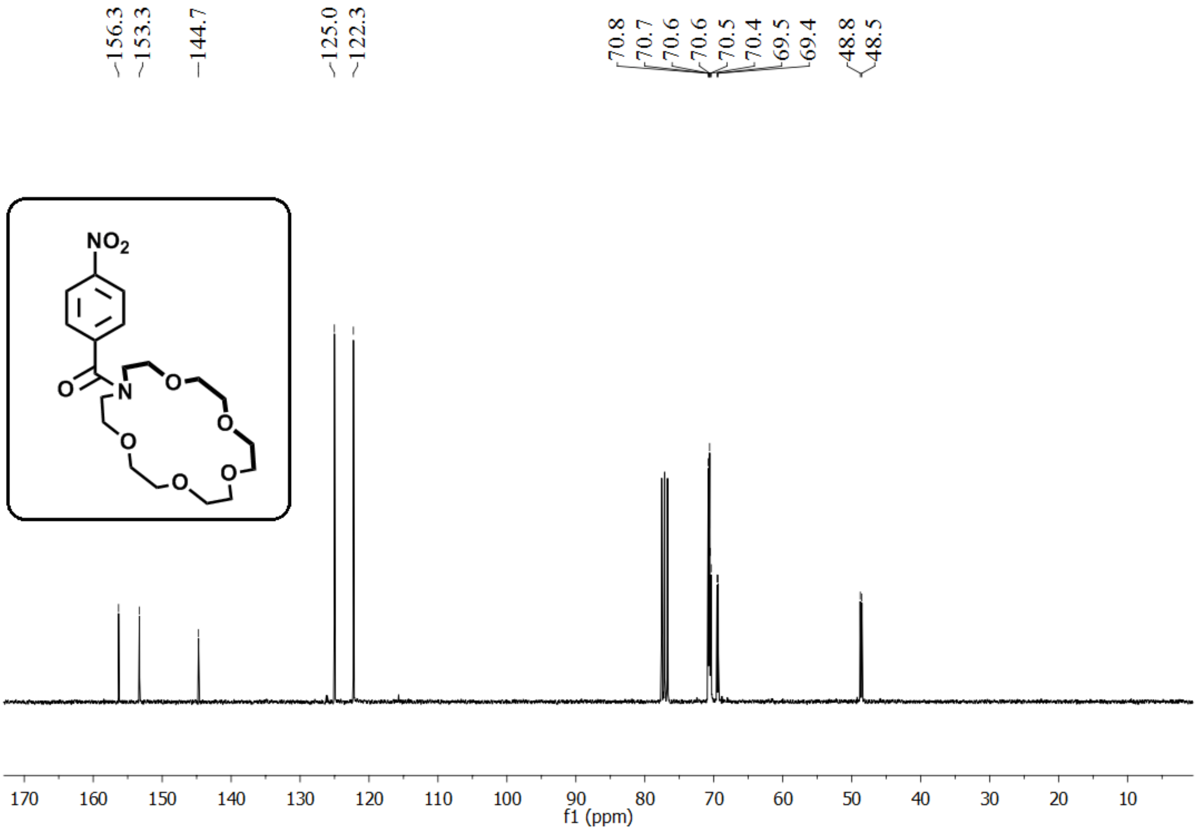
156.3
153.3
144.7
125.0
122.3
70.8
70.7
70.6
70.6
70.5
70.4
69.5
69.4
48.8
48.5
NO2
O
N
O
O
O
O
170 160 150 140 130 120 110 100 90 80 70 60 50 40 30 20 10
f1 (ppm)

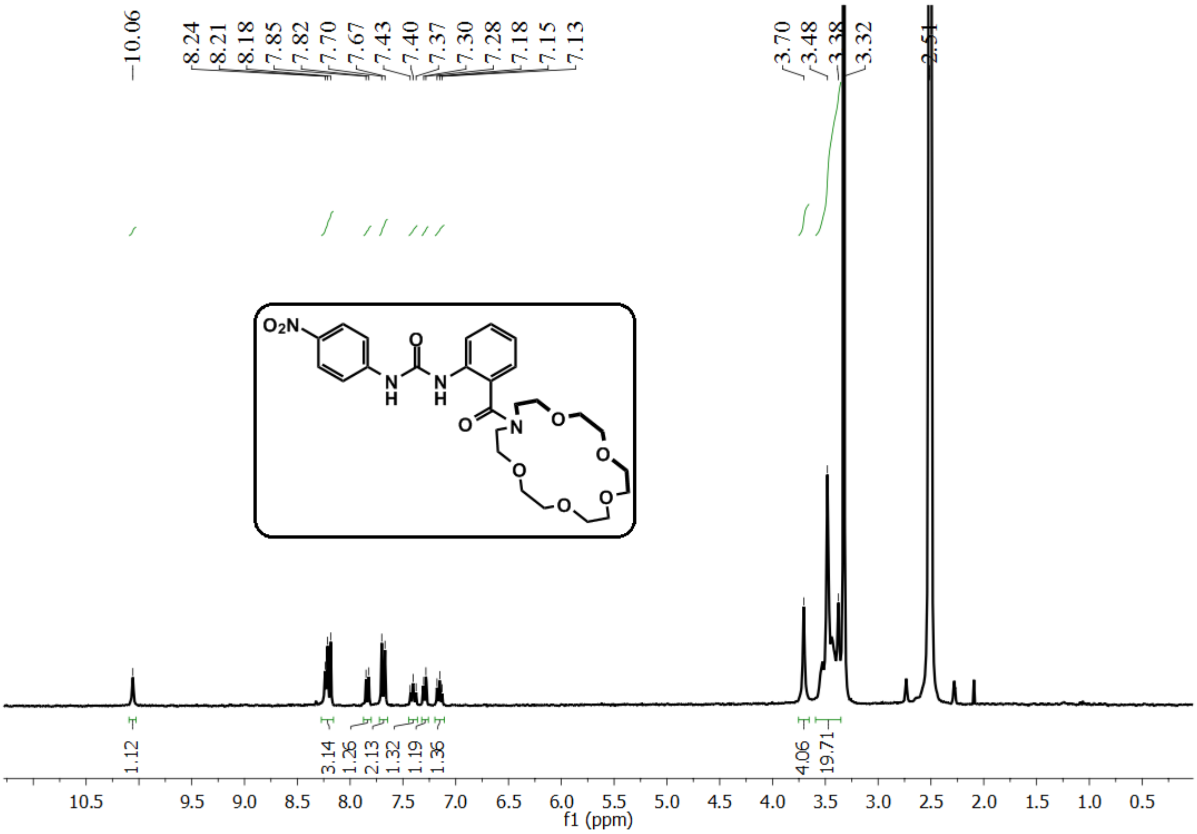
10.06
8.24
8.21
8.18
7.85
7.82
7.70
7.67
7.43
7.40
7.37
7.30
7.28
7.18
7.15
7.13
3.70
3.48
3.38
3.32
2.51
O2N
N
H
O
1.12
3.14
1.26
2.13
1.32
1.19
1.36
4.06
19.71
f1 (ppm)

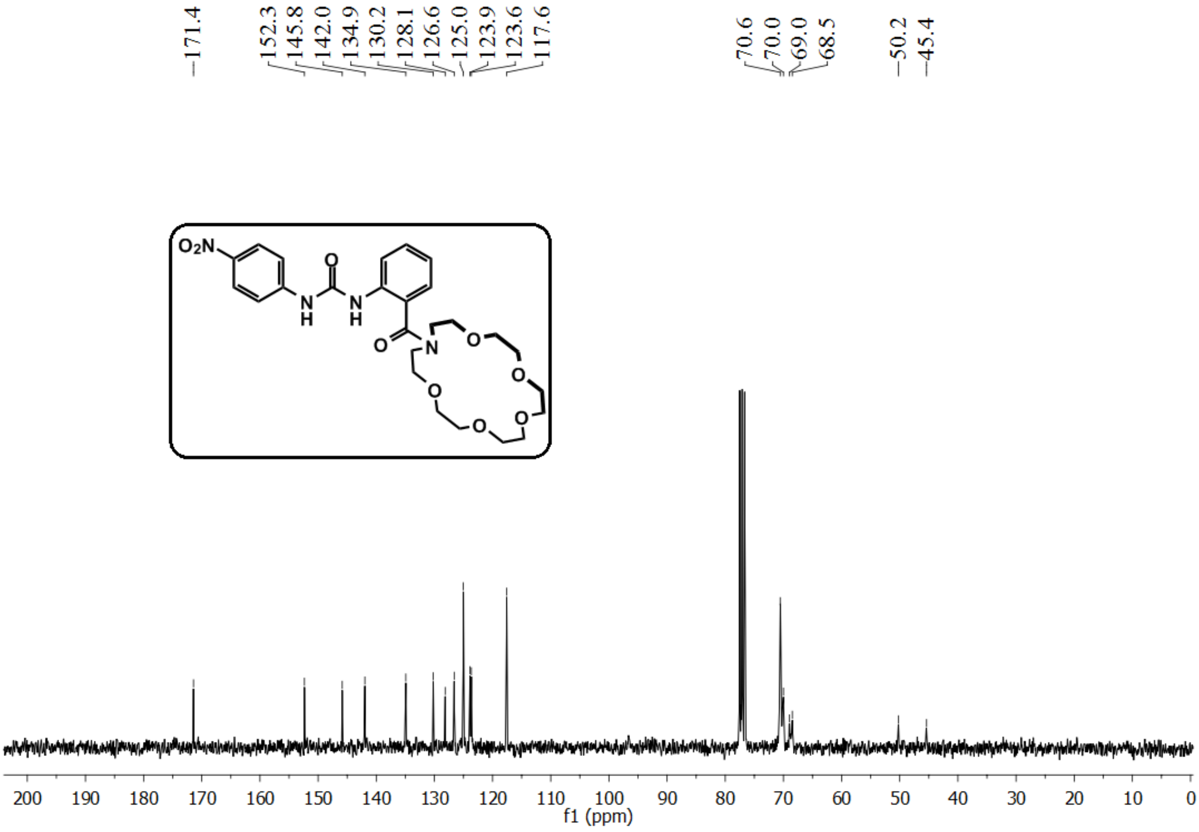
171.4
152.3
145.8
142.0
134.9
130.2
128.1
126.6
125.0
123.9
123.6
117.6
70.6
70.0
69.0
68.5
50.2
45.4
O2N
N
H
O
f1 (ppm)

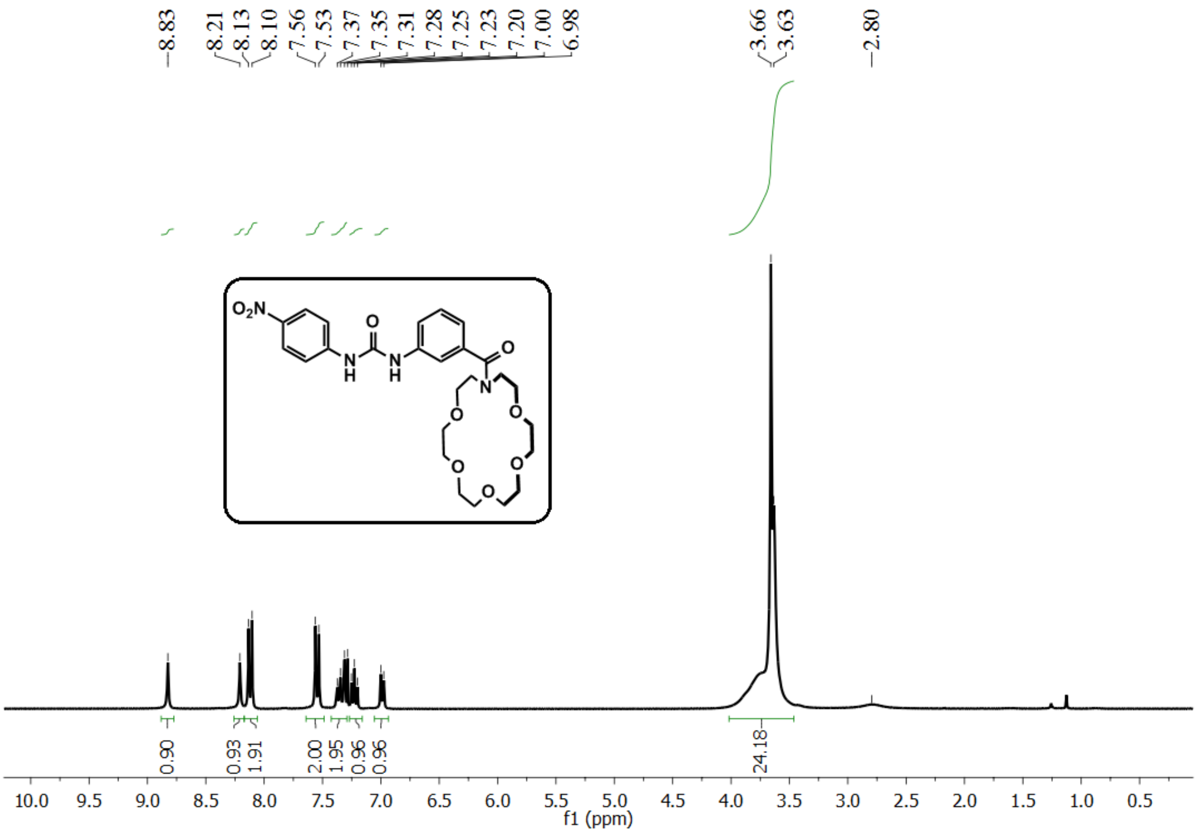
8.83
8.21
8.13
8.10
7.56
7.53
7.37
7.35
7.31
7.28
7.25
7.23
7.20
7.00
6.98
3.66
3.63
2.80
O2N
O
N
H
N
H
O
N
O
O
O
O
0.90
0.93
1.91
2.00
1.95
0.96
0.96
24.18
10.0
9.5
9.0
8.5
8.0
7.5
7.0
6.5
6.0
5.5
5.0
4.5
4.0
3.5
3.0
2.5
2.0
1.5
1.0
0.5
f1 (ppm)

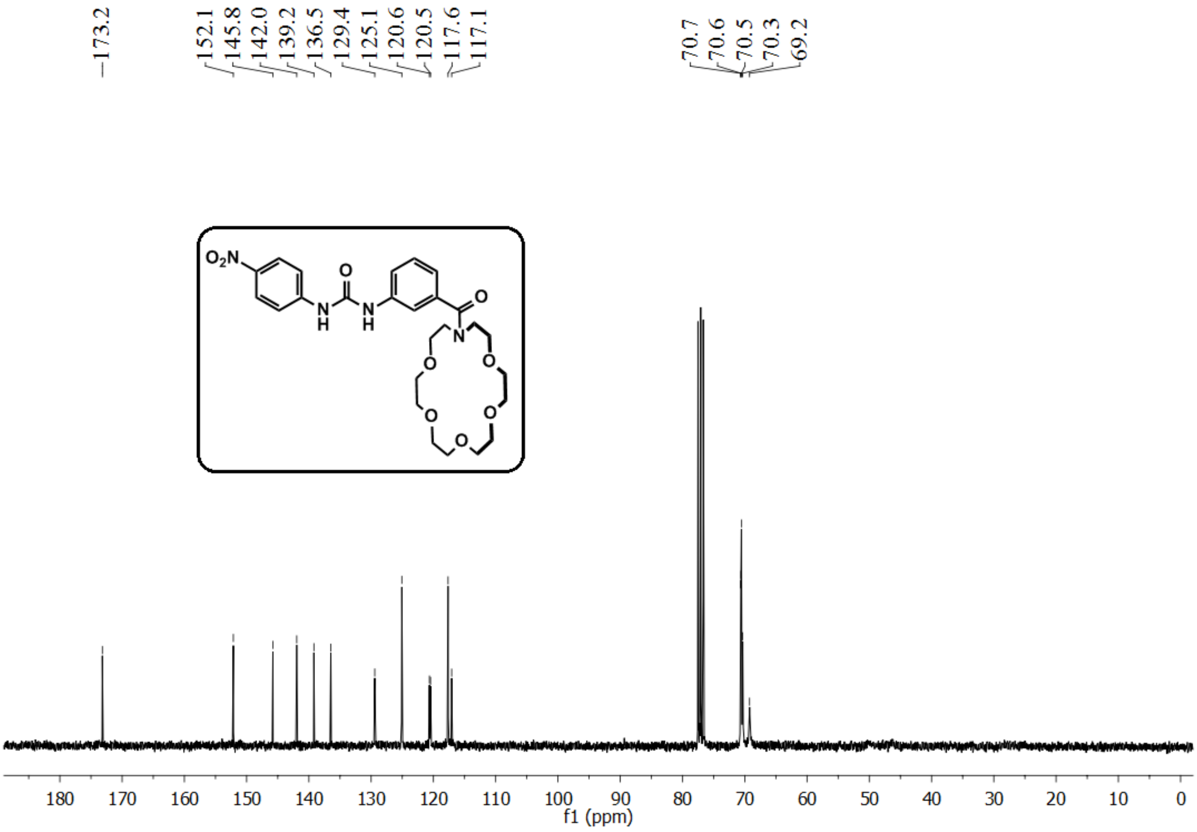
173.2
152.1
145.8
142.0
139.2
136.5
129.4
125.1
120.6
120.5
117.6
117.1
70.7
70.6
70.5
70.3
69.2
O2N
O
N
H
N
H
O
N
O
O
O
O
180
170
160
150
140
130
120
110
100
90
80
70
60
50
40
30
20
10
0
f1 (ppm)

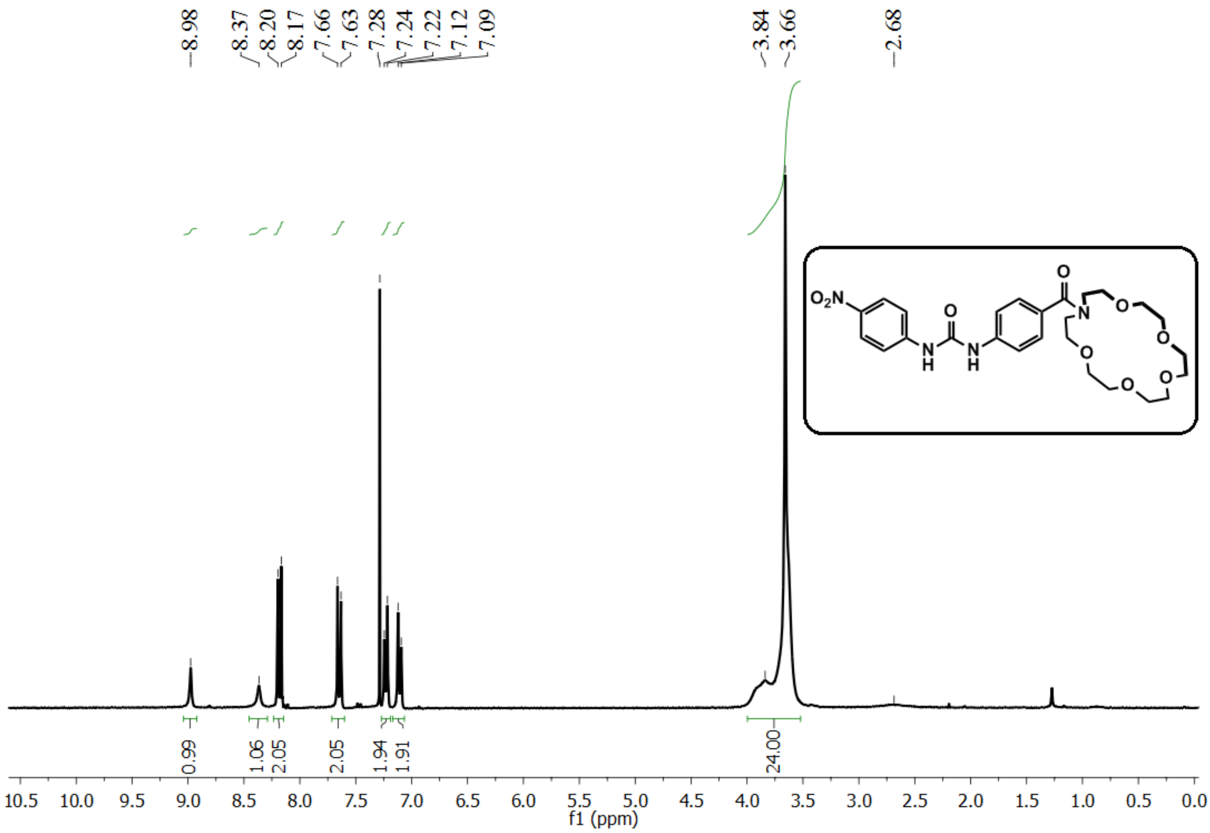

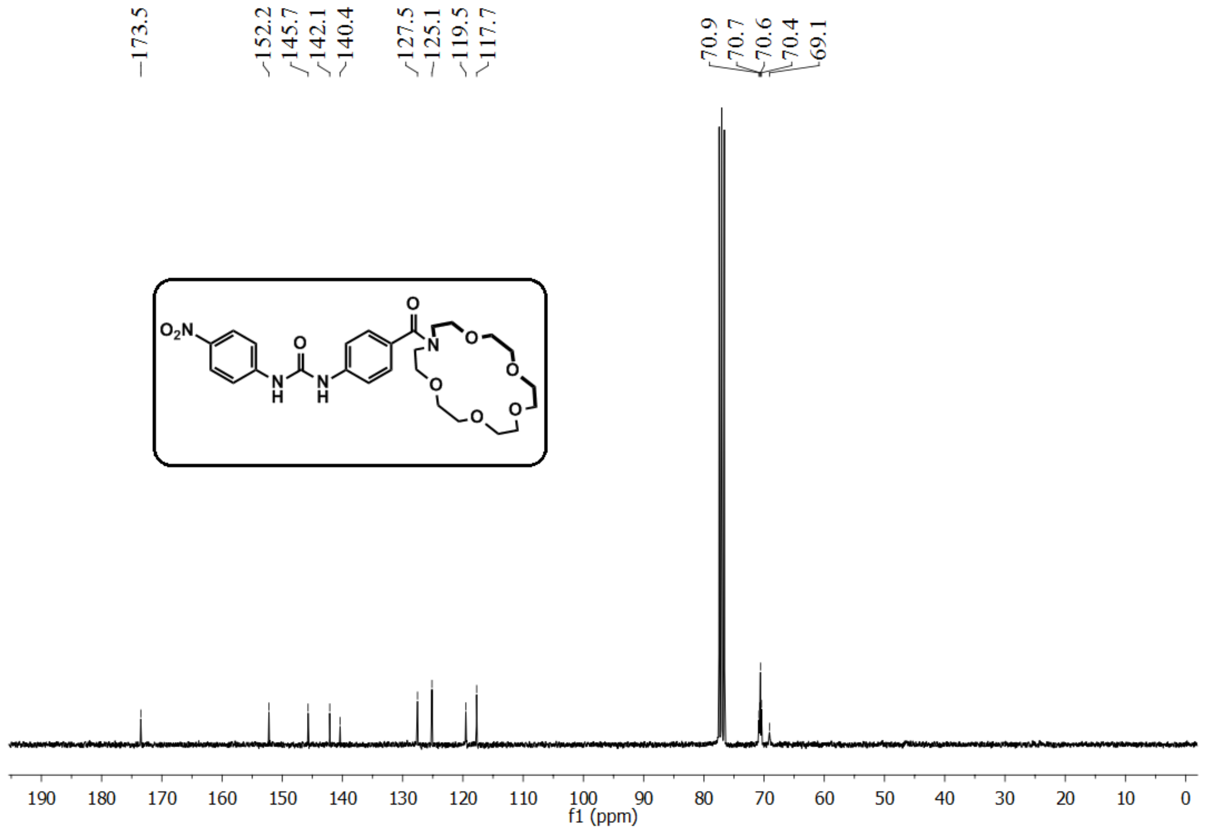

REFERENCES

[1] (a) APEX2, Bruker AXS Inc., Madison,Wisconsin, USA, **2013**; (b) SAINT, BrukerAXS Inc., Madison, Wisconsin, USA, **2013**; (c) SADABS, Bruker AXS Inc., Madison,Wisconsin, USA, **2012**.

[2] (a) Sheldrick, G.M. *ActaCryst.* **1990**, *A46*, 467-473; Sheldrick, G.M. *ActaCryst.*, **2008**, *A64*, 112-122.

[3] Wilson, A.J.C. International Tables for Crystallography. *vol. C*. Dordrecht: Kluwer, **1992**.

[4] Ziach, K.; Jurczak, J. Chiral Crystals from Dynamic Combinatorial Libraries of Achiral Macrocyclic Imines, *Cryst. Growth Des.* **2015**, 15, 4372-4376
